# Resolving the fundamentals of the *J*-integral concept by multi-method in situ nanoscale stress-strain mapping

**DOI:** 10.1038/s43246-025-00752-z

**Published:** 2025-02-22

**Authors:** Michael Meindlhumer, Markus Alfreider, Noel Sheshi, Anton Hohenwarter, Juraj Todt, Martin Rosenthal, Manfred Burghammer, Enrico Salvati, Jozef Keckes, Daniel Kiener

**Affiliations:** 1 Department of Materials Science, Montanuniversität Leoben, Leoben, Austria; 2 Polytechnic Department of Engineering and Architecture (DPIA), University of Udine, Udine, Italy; 3 ESRF – The European Synchrotron, Grenoble, France; 4 Present Address: Department of Chemistry, KU Leuven, Leuven, Belgium

**Keywords:** Nanoscale materials, Mechanical properties, Characterization and analytical techniques

## Abstract

The integrity of structural materials is oftentimes defined by their resistance against catastrophic failure through dissipative plastic processes at the crack tip, commonly quantified by the *J*-integral concept. However, to date the experimental stress and strain fields necessary to quantify the *J*-integral associated with local crack propagation in its original integral form were inaccessible. Here, we present a multi-method nanoscale strain- and stress-mapping surrounding a growing crack tip in two identical miniaturized fracture specimens made from a nanocrystalline FeCrMnNiCo high-entropy alloy. The respective samples were tested in situ in a scanning electron microscope and a synchrotron X-ray nanodiffraction setup, with detailed analyzes of loading states during elastic loading, crack tip blunting and general yielding, corroborated by a detailed elastic-plastic finite element model. This complementary in situ methodology uniquely enabled a detailed quantification of the *J*-integral along different integration paths from experimental nanoscale stress and strain fields. We find that conventional linear-elastic and elastic-plastic models, typically used to interpret fracture phenomena, have limited applicability at micron to nanoscale distances from propagating cracks. This for the first time unravels a limit to the path-independence of the *J*-integral, which has significant implications in the development and assessment of modern damage-tolerant materials and microstructures.

## Abstract (web summary)

The *J*-integral is used to quantify resistance to crack propagation. Here, a detailed quantification of the *J*-integral is demonstrated for a high entropy alloy, by measuring the nanoscale strain and stress fields around crack tips via in situ testing and X-ray nanodiffraction.

## Introduction

Comprehending and quantifying fracture characteristics constitute pivotal steps in mitigating structural component failure and enhancing the longevity of modern materials in various applications. To quantitatively assess the fracture resistance of (brittle) materials, linear-elastic fracture mechanics (LEFM) was developed over 100 years ago^1,2^. It is based on thermodynamic considerations linking the energy released upon crack extension to the energy required for creation of new fracture surfaces. The resulting stress *σ* and strain *ε* solutions around the crack tip contain mathematical singularities originating from a ${r}^{-0.5}$ term, where *r* is the radial distance from the crack tip. LEFM deals with these singularities by focusing on the elastically loaded region outside the plastic zone (PZ) in the closest crack tip vicinity and scaling the ${r}^{-0.5}$ behaviour with so-called stress intensity factors $K$. LEFM and its extension to small-scale yielding are applicable when the elastic stress field around the crack tip^1^, the so-called *K*-field, is dominant (in size) and crack tip plasticity is minimal. Consequently, it is restricted to brittle or semi-brittle materials or specimen geometries of unwieldy size, up to meters for very ductile materials.

When small-scale yielding conditions are not met, elastic-plastic fracture mechanics concepts, such as the crack-tip opening displacement^3^ and *J*-integral^4^ are necessitated. While the first is a purely geometric concept, the latter is based on energetic considerations of an isotropic Ramberg-Osgood^5^ type hardening material. Close to the crack tip, where the (e.g. von Mises) stress exceeds the material yield stress (hence, within the PZ), the stress and strain increase is linked to the *J*-integral, with a proportionality of ${\sigma \propto r}^{-1/n+1}$ and ${\varepsilon \propto r}^{-n/n+1}$, (*n* being the Ramberg and Osgood hardening parameter^5^), analogous to *K* and ${r}^{-0.5}$ in LEFM. While this so-called HRR-theory (after Hutchinson^6^, Rice and Rosengren^7^) also deals with singularities when reaching the crack tip and thus also neglects material decohesion, it is able to incorporate plastic deformation in front of the crack tip^6,7^. In linear-elastic materials^8^ ${J=K}^{2}\cdot \frac{1-{\nu }^{2}}{E}$, where *E* and *ν* are the elastic modulus and the Poisson’s ratio, respectively. The accurate mathematical definition of *J* is based on a positive contour integral around a 2D crack tip, which is path independent if specific simplified assumptions hold^4^. The subjects of integration include the non-linear strain energy density and the traction forces normal to the integration contour, which include the stress and strain field around the crack tip. Therefore, the only possibility to directly assess *J* as a function of varying contour paths^4^ is *combined local strain and stress field mapping* in the crack tip vicinity.

There have been several attempts to measure the individual elastic, plastic and/or total strains and stresses in front of a crack tip. Synchrotron or neutron diffraction have been proven vital to evaluate the elastic strain contribution (transforming it directly into stress) in front of cyclically^9–15^ and unidirectionally loaded^16–18^ cracks. Recently, cross-sectional X-ray nanodiffraction (CSnanoXRD) has been employed to investigate the multiaxial stress fields of multilayers during and after severe deformation^19,20^ or during crack growth^18^, resolving not only the 2D stress tensor perpendicular to the incident focused X-ray beam, but also the size and shape of the plastic zone^18^. To quantify crack growth^21^ and the total strain in front of the crack, tip digital image correlation (DIC) techniques are state-of-the-art^22^. Furthermore, X-ray diffraction-based elastic strain analysis and DIC total strain analysis were combined to explore stress and strain field evolution resulting from overload events during cyclic loading^12^. Another recent in situ method combines electron backscatter diffraction (EBSD) with strain tracking by DIC of a speckle pattern on the sample surface^23^. Though this approach provides superior total strain resolution by DIC in the scanning electron microscope (SEM) and adequate elastic strain resolution from the EBSD patterns, both at nearly the same volume depth, it is restricted to small deformation steps and single-crystalline or coarse-grained materials. In contrast, an in situ SEM-DIC based method proposed by Alfreider et al.^24^ utilizes pre-milled point feature tracking for the determination of total strain on the sample surface while also considering large plastic deformations during a *continuous straining experiment*. Combined, these advancements let to this study’s primary objective: the one-of-a-kind direct experimental evaluation of the *J*-integral during in situ loading of a fracture specimen. Such accomplishment uniquely enables the verification of established fracture mechanics concepts in a realistic experiment, their validity and boundary conditions, with all caveats and unexpected deformation characteristics that might occur in modern highly damage tolerant materials^25–28^.

High entropy alloys^29^ are increasingly investigated for their alleged chemical, thermal and mechanical stability^30,31^, which may enable new sustainable materials design^32^ based on promising mechanical properties of this new material class^33–35^. For example, a NbTaTiHf alloy exhibits superior room temperature fracture toughness by a dynamic competition of screw and edge dislocations at the crack tip^28^, while multiplicity of dislocation pathways and negative mixing enthalpy promote high ductility in MoNbTi^26^ and HfNbTiVAl_10_^27^, respectively. Especially the herein studied Cantor alloy (CoCrFeMnNi)^36^ has drawn significant attention due to its enormous fracture toughness of ~270 MPa m^½^ even at cryogenic temperatures^25^, while it retains its ductility and high yield strength in nanocrystalline state^24,37^. This renders it a perfect material for the objective of this work, the validity assessment of local *J*-integral analysis via simultaneous investigation of the *local stress* (elastic deformation) and *strain state* (total deformation) *in front of a crack tip*. Additionally, the high yield stress ${\sigma }_{{{{\rm{y}}}}}$ of nanocrystalline materials renders them desirable for responsible device design^38,39^, but synthesis of bulk nanocrystalline materials for conventional techniques and sample sizes is challenging. We overcome this challenge by utilizing micromechanical testing methods^40–45^.

**Fig. 1:**
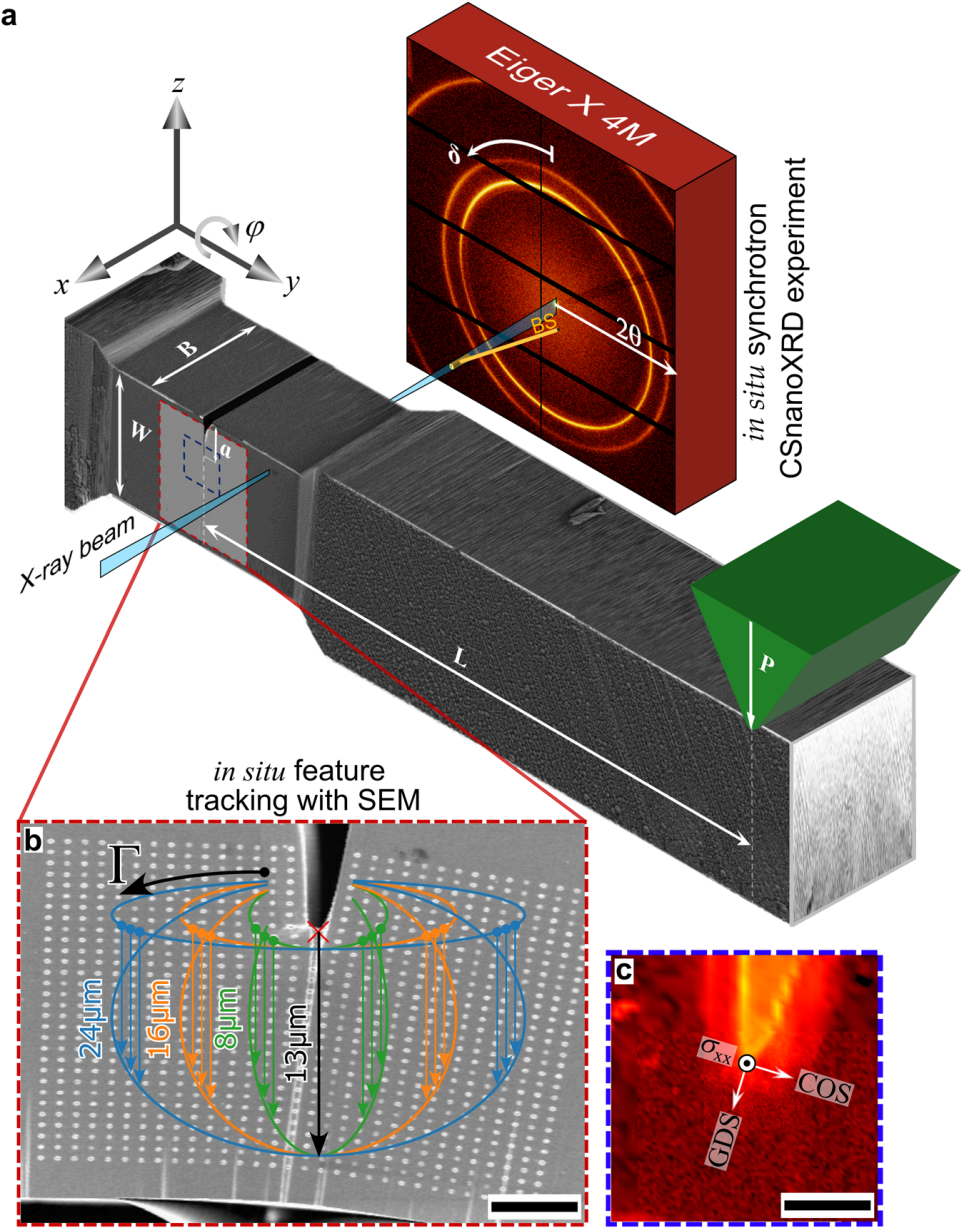
Schematic visualization of in situ synchrotron and in situ SEM experiments. Here, *L*, *B*, and *W* are the bending length, thickness and width of the cantilevers, respectively, while *a* is the crack length and BS indicates the beam stop (**a**). The cantilever deformed in SEM was continuously loaded, and a video recorded for tracking the spots to evaluate the total surface strains (**b**). Additionally, the elliptic lines in (**b**) show integration contours for *J*-integral evaluation, being small (8 µm), medium (16 µm) and wide (24 µm) ellipses at closest (1 µm) and maximum crack tip distance (13 µm) configurations, respectively. In (**c**) a detail of a SAXSM micrograph around the crack tip is shown with the coordinate system used to evaluate the relevant crack opening stress (COS) and growth direction stress (GDS) components parallel and perpendicular to the local crack tip coordinate system, respectively. The scale bars in (**b** and **c**) are 5 µm.

In this work, we conduct a thorough analysis of local stress and strain tensors by in situ CSnanoXRD (Fig. 1a) and in situ SEM-DIC (Fig. 1b) on two nearly identical microcantilevers with ${\left(a/W\right)}_{{{{\rm{CSnano}}}}X{{{\rm{xRD}}}}}=0.347\pm 0.005$ and ${\left(a/W\right)}_{{{{\rm{SEM}}}}}=0.351\pm 0.005$, respectively, while the bending lengths *L* was adapted to the differing widths *B* (5% difference between CSnanoXRD and SEM cantilevers) to apply a nominally equal stress intensity at the crack tip. The in situ SEM experiment involved continuous quasi-static deformation, while the in situ CSnanoXRD experiment was performed stepwise with an adapted bending length adjusting for similar nominal *K*_I_ at the crack tip, guided by preceding SEM load-displacement data. Crack growth was monitored in SEM by sequential unloading, which was further used for standardized global calculation of the *J-*integral, while small-angle X-ray scattering (Fig. 1a, c)^46^ and full width at half maximum (FWHM) microscopy^19^ were utilized to track crack growth and microstructural changes during the CSnanoXRD experiment, respectively. Additionally, the experimental data were complemented by a 3D elastic-plastic finite element (FE) model. Combining experimental stress and strain data enabled to calculate the *J-*integral as a function of the contour distance from the crack tip in three fundamental load regimes: during elastic loading (LS1), crack tip blunting (LS2) and general yielding (LS3). This unprecedented combination of two in situ nanoscale approaches is employed for the first time to directly assess an experimental *J*-integral (*J*_comb_) at the close crack tip vicinity, reflecting its original theoretic description^4^. The evaluation conducted with varying integration paths (Fig. 1b) indicates a strong path dependence of the *J*-integral within the plastically deformed region, in contrast to the original concept. Furthermore, it allowed novel insights into the defect dominated deformation and fracture behaviour of nanocrystalline high entropy alloys to guide future material design.

## Results

### Representative multi-method in situ data

Here only representative correlative in situ data will be presented from two near-identical cantilevers. Full details regarding mechanical tensile characteristics of the material as well as similarity of the experiments and implications on the here presented results will be given in Supplementary Notes 1 and 2, respectively.

**Fig. 2:**
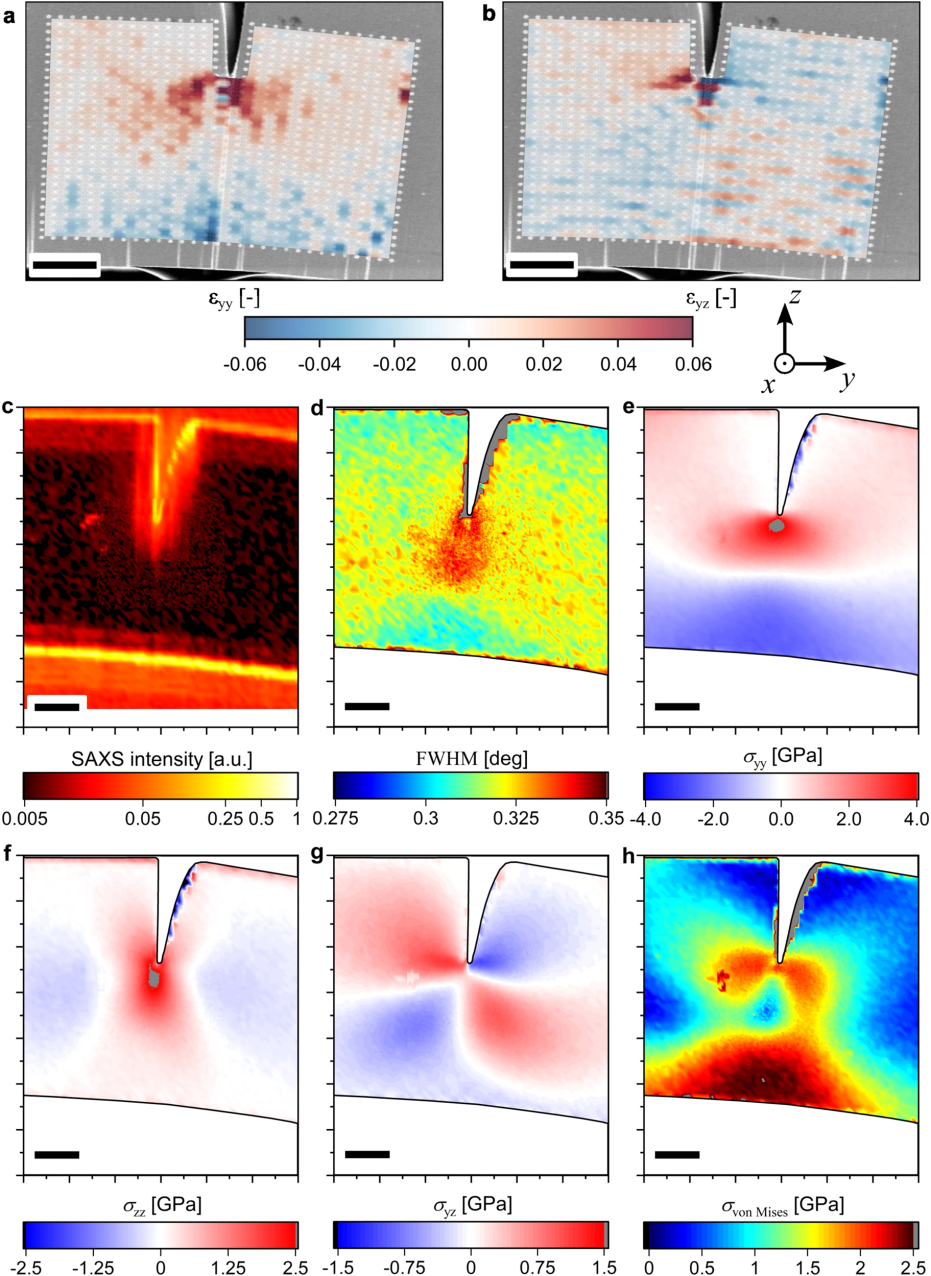
Representative in situ SEM and CSnanoXRD data at LS2. In situ SEM data (**a**, **b**) showing total normal *ε*_yy_-strains and shear *ε*_yz_-strains. The CSnanoXRD data (**c**–**h**) provides SAXS micrographs (**c**) used for quantitative crack growth evaluation, while average FWHM data (**d**) details qualitatively the microstructural evolution. Stress components *σ*_yy_, *σ*_zz_, and *σ*_yz_ are given in (**e**–**g**), while the *σ*_von Mises_ distribution calculated from the respective stress components is shown in (**h**). All scale bars correspond to 5 µm.

Figure 2a, b depict the normal *ε*_yy_ and shear *ε*_yz_ strain at LS2 as representative strain components, respectively. The normal strain depicts a butterfly-shape region in front of the crack tip as described by classical fracture theories^1,6–8^. Furthermore, a transitional gradient from high tensile to high compressive stresses as a result from the bending geometry of the specimen is evident. The shear strains show slight stochasticity in the form of unphysical compression/tensile undulations. However, the antisymmetric trend for shear strains in front of a crack tip is evident^1,6–8^. Detailed strain components (*ε*_yy_, *ε*_zz_, *ε*_yz_, and *ε*_xx_) for all loadsteps in analogy to the CSnanoXRD data as well as the full videos compiled thereof are provided in Supplementary Note 3 (Supplementary Fig. S4 and Movies S1–S4).

Again, the acquired microstructure and stress data obtained at LS2 in the in situ CSnanoXRD experiment are shown in Fig. 2c–h, while CSnanoXRD maps for all individual loadsteps are fully detailed in Supplementary Note 4. The small-angle X-ray scattering microscopy (SAXSM) micrograph in Fig. 2c shows that the crack length increased to 11.4 ± 0.5 µm with clearly detectable crack tip blunting. The advantage of SAXSM against current electron microscopy-based crack length detection is that each pixel in the 2D plot is averaged over the cantilever thickness, providing a volumetric crack length average representative for the whole specimen rather than only a surface crack length.

The averaged FWHM map at LS2 in Fig. 2d shows an increase in the area up to ~8 µm in front of the crack tip, indicating defect accumulation (e.g. dislocation emission, stacking faults) due to plastic deformation originating from applying mechanical load on the crack tip. However, compared to LS1, the FWHM is decreased in the immediate crack tip vicinity (compare Supplementary Fig. S7 and Supplementary Note 4). Conversely, a decrease of the FWHM is evident in the highly compressive region at the lower part of the specimen. This indicates recombination/removal of mobile defects introduced through the high pressure torsion (HPT) process^47–49^.

The *σ*_yy_, *σ*_zz_, and *σ*_yz_ stress distributions obtained at LS2 are presented in Fig. 2e–g, respectively. While the *σ*_yy_ stress distribution exhibits a superposition of the bending and crack tip stress fields (Fig. 2e), the *σ*_zz_ and *σ*_yz_ stress distributions are governed exclusively by the crack tip stress field (Fig. 2f, g, respectively), according to fracture mechanics theory^8^.

${\sigma }_{{{{\rm{von\; Mises}}}}}\left(y,z\right)$ at LS2 (Fig. 2h) exhibits a typical butterfly-like stress distribution ahead of the crack tip. The maximum of ${\sigma }_{{{{\rm{von\; Mises}}}}}\left(y,z\right)$ was found to be ~2.04 GPa, close to the reported yield stress between ~2 GPa^37^ and 2.35 GPa^24^ for this specific nanocrystalline high entropy alloy (HEA). However, at LS2, the maximum ${\sigma }_{{{{\rm{von\; Mises}}}}}\left(y,z\right)$ value of ~2.51 GPa was found at the lower cantilever half, where the stress state is close to uniaxial compression (*cf*. Fig. 2e–g). Analysis of ${\sigma }_{{{{\rm{von\; Mises}}}}}\left(y,z\right)$ magnitudes thus indicates that the yield stress is reached both in front of the crack tip as well as at the bottom of the cantilever.

### Individual deformation regimes

**Fig. 3:**
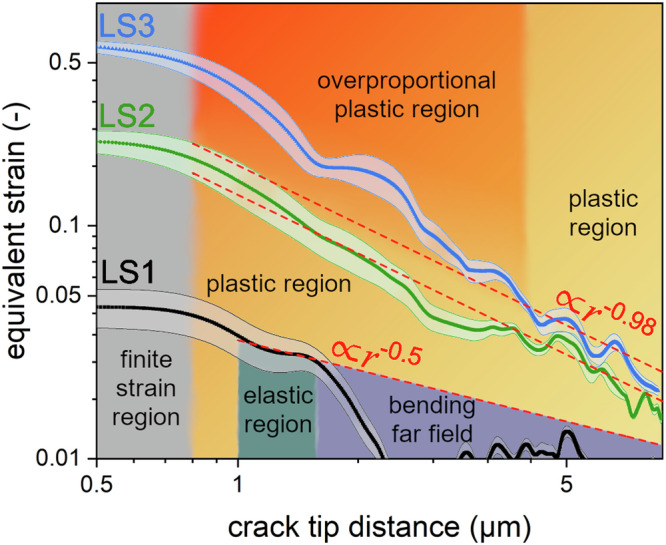
Evolution of the equivalent crack tip strains. Double logarithmic plot of average equivalent strain in the highly deformed regions (±30°−60°) in front of the crack tip for each loading step. The transparent bound depicts the respective standard errors for each crack tip distance, while dashed lines act as guides for the eye and indicate the strain dependency necessary for valid *K*- (*r*^−0.5^) or HRR- (*r*^−0.98^) descriptions, respectively.

**Fig. 4:**
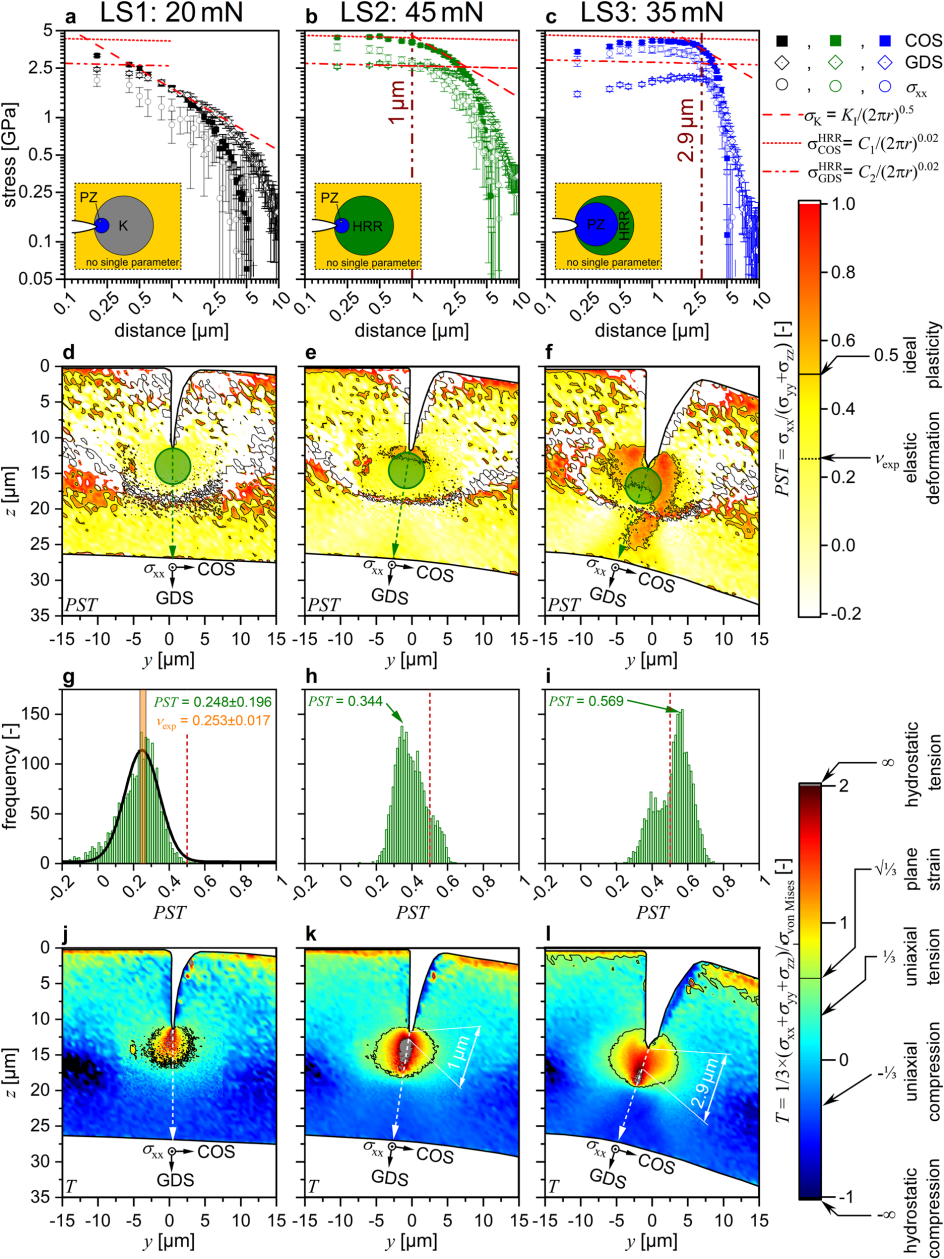
Crack tip stress characteristics upon loading. The crack opening stresses (COS), growth direction stresses (GDS) and *σ*_xx_ are shown for LS1, LS2, and LS3 in (**a**–**c**), respectively. The PST value introduced in Eq. 2 to verify the evaluated crack tip stresses is presented in (**d**–**f**), respectively, where the black contour line marks PST = 0.5, which can be attributed to full plastic deformation. Additionally, circles with a diameter of 5 µm in front of the crack tip represent the area from which values were taken for the statistical analysis shown in (**g**–**i**). Additionally, Poisson’s ratio *υ*_exp_ = 0.253 ± 0.017^24^ for this HEA is indicated in (**g**). Finally, in (**j**–**l**) the triaxiality ratio, being the relation between the hydrostatic portion of the stress tensor and the von-Mises stress representing the strain energy of distortion, is shown. The black contour line in (**j**–**l**) corresponds to $T=1/\sqrt{3}\approx 0.577$, representing a plane strain state. The error bars in (**a**–**c**) depict the standard deviation of the stress results.

**Fig. 5:**
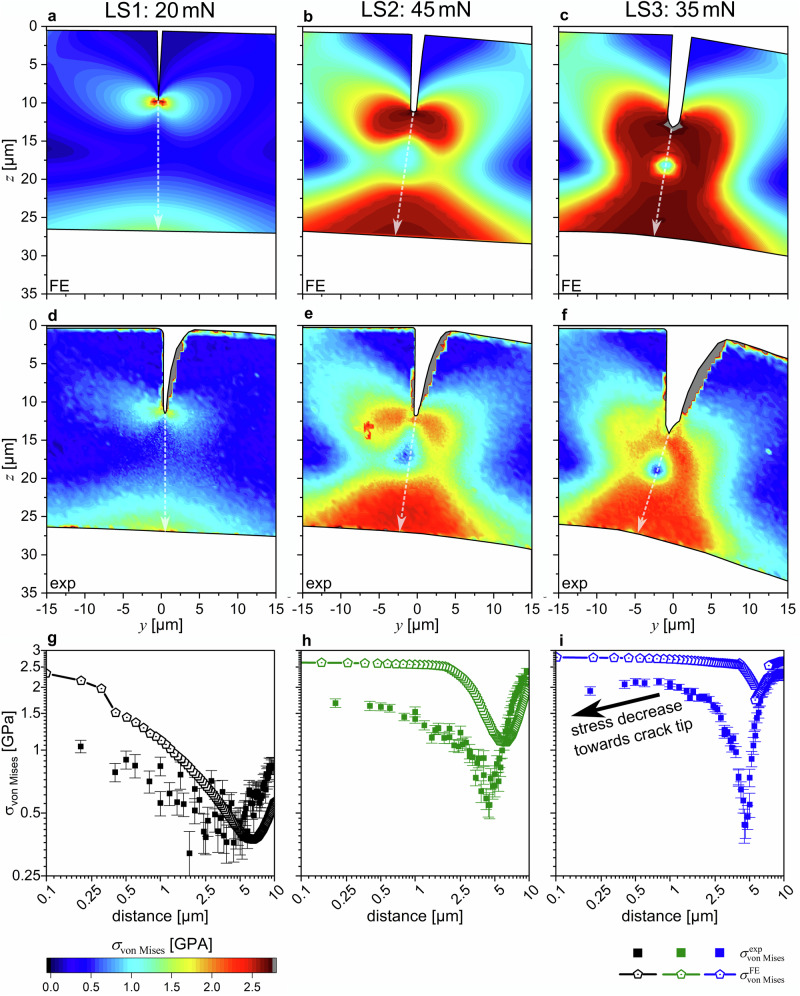
Comparison between FE-model and the experimental data. Modeled and experimental *σ*_von Mises_ distributions are shown in for LS1, LS2, and LS3 in (**a**–**c**) as well as (**d**–**f**), respectively. The evaluated crack tip von Mises stresses are presented in (**d**–**f**), respectively, where the full and open symbols indicate the experimental and modeled data. The error bars in (**g**–**i**) depict the standard deviation of the experimental stress results.

Figure 3 shows the equivalent crack tip strains in the highly deformed regions (±30–60 deg) gathered from the in situ SEM experiment for all loadsteps, while Fig. 4 sums up relevant characteristics for the three fundamental regimes: elastic loading (LS1, first column), transition to plastic deformation (LS2, second column) and general yielding (LS3, third column), respectively. Thereby, the first row depicts the stresses in front of the crack tip, the second row depicts maps of the plane-strain test plane strain test (PST) (*cf*. Methods) and the near crack tip region (green circle with a diameter of 5 µm) from which the respective PST distributions in the third row are taken. PST can be correlated with the Poisson’s ratio *ν* = 0.253 ± 0.017^24^ of the present material. The fourth row details individual maps of stress-triaxiality *T*. Again, full details of the CSnanoXRD stress characteristics are given in Supplementary Note 4. Additionally, FE modelling was performed to corroborate the experimental data and Fig. 5 shows the comparison between the experimental and modelled *σ*_von Mises_ distributions. Full details regarding the results from the FE model are given in Supplementary Note 5.

At LS1, Fig. 3 shows that the maximum crack tip strains rise to ~0.05, already beyond the elastic limit in tension experiments^24^, suggesting finite plastic deformation at the crack tip. After a limited region with a *r*^−0.5^ decrease, which suggests the transition to a linear elastic *K*-field, a considerably steeper decrease of the strain is evident at ~1.5 µm crack tip distance due to the bending gradient dominating the deformation.

The stress distributions are mainly controlled by the elastic (*K*-) field (Fig. 4a). Deviations occur very close to (<200 nm) and far from (>2.5 µm) the crack, indicating the presence of PZ and bending stress gradient, respectively. The PST ratio (Fig. 4d), used to determine the stress state (Eq. 2, Methods), increases slightly towards the notch and aligns well with reported Poisson’s ratios of *υ*_exp_ of 0.253 ± 0.017^24^ and 0.25 ± 0.1^50^ of the FeCrMnNiCo HEA (Fig. 4g). This confirms that the cantilever is mostly under plane-strain conditions near the notch, conforming to elastic fracture mechanics theory^8^.

Similar to the PST ratio, the stress triaxiality ratio *T* (Eq. 3) provides insight into the nature of the stress concentrations in front of the notch. Generally, a high tensile stress triaxiality favors crack growth by void formation and coalescence over plastic shear deformation, i.e. shear lip formation^8,51^.

Generally, at LS1, the comparison between the experimental data and the FE-model yields excellent agreement outside the immediate crack tip vicinity (see Supplementary Fig. S14, Fig. 5a and d). The lower experimental von Mises stress magnitudes directly in front of the crack tip (Fig. 5g) may be related to the (i) finite notch radius obtained by the focused ion beam cantilever preparation, (ii) the finite X-ray gauge volume and (iii) the potentially slightly exaggerated experimental *σ*_xx_ magnitudes.

After loading to 45 mN (LS2), the strain in front of the crack tip significantly increases, in overall agreement with the HRR model (*r*^−0.98^, for *n* = 50, see Supplementary Note 1)^5,24^, but again deviating near the crack tip indicating a region of finite deformation. Stress values around the notch suggest the crack tip vicinity (<1 µm) being governed mainly by plastic deformation, aligning with HRR theory^6,7^ (Fig. 4b). The PST ratio in LS2 rises towards the crack tip, indicating ideal plastic deformation (Fig. 4e), which is confirmed by the frequency distribution of the PST ratio (Fig. 4h).

Additionally, the stress triaxiality *T* indicates higher hydrostatic tensile stress components near the crack in a circular zone extending ~5 µm (*T* > $1/\sqrt{3}$), consistent with plastic deformation and potential void formation (Fig. 4k). However, up to 1 µm from the crack tip *T* amounts below 2, in good agreement with the range of the HRR field identified in Fig. 4b, indicating plastic deformation and likely onset of void formation in front of the crack tip.

Also, at LS2, the comparison between the experimental data and the FE-model yields very good agreement outside the immediate crack tip vicinity (Supplementary Fig. S4b and e, Fig. 5b and e). Compared to LS1, the deviations of the experimental and modeled *σ*_von Mises_ magnitudes increase, while the overall trend of increasing *σ*_von Mises_ magnitudes towards the crack tip remains similar for both experiment and model.

At LS3, the strains near the crack tip increase further, reaching about 0.5 and deviating substantially from the theoretical slope of *r*^−^^0.98^ ^5–7^ up to a crack tip distance of ~4 µm (Fig. 3). The observed stress values (Fig. 4c) are drastically reduced and differ significantly from each other (COS > *σ*_xx_ > GDS), favoring plastic deformation over crack growth in front of the crack tip (*cf*. Fig. 4l). The distinct zones of plastic, elastic, and bending-dominated regions blend together, showing continuous transitions (Fig. 4c). The PST ratio increases further at LS3 (Fig. 4f), which indicates complete plastic deformation^52^ and suggests even some major pore formation due to the average exceeding the 0.5 threshold (Fig. 4i) from constant incompressible volume arguments^53^, in agreement with the pores found by the post-experiment SEM analysis (Supplementary Note 2). Additionally, *T* decreases near the crack tip (Fig. 4l), substantiating plastic deformation rather than crack growth.

At LS3, the FE-model yields further increase of the *σ*_von Mises_ stress magnitudes as can be drawn from Fig. 5c. The differences are for the first time clearly not only restricted to the immediate crack tip area (Fig. 5c and f). Furthermore, the effect of strain softening is highlighted in the experimental data, where in contrast to the model, *σ*_von Mises_ decreases with decreasing distance from the crack tip (Fig. 5i). It is worth to remind that the FEM model employed the Ramberg-Osgood hardening fit to the experimental tensile data (Supplementary Note 1) as a constitutive law, which can not account for the strain softening—this explains such a mismatch in this region.

### *J*-integral calculations

**Fig. 6:**
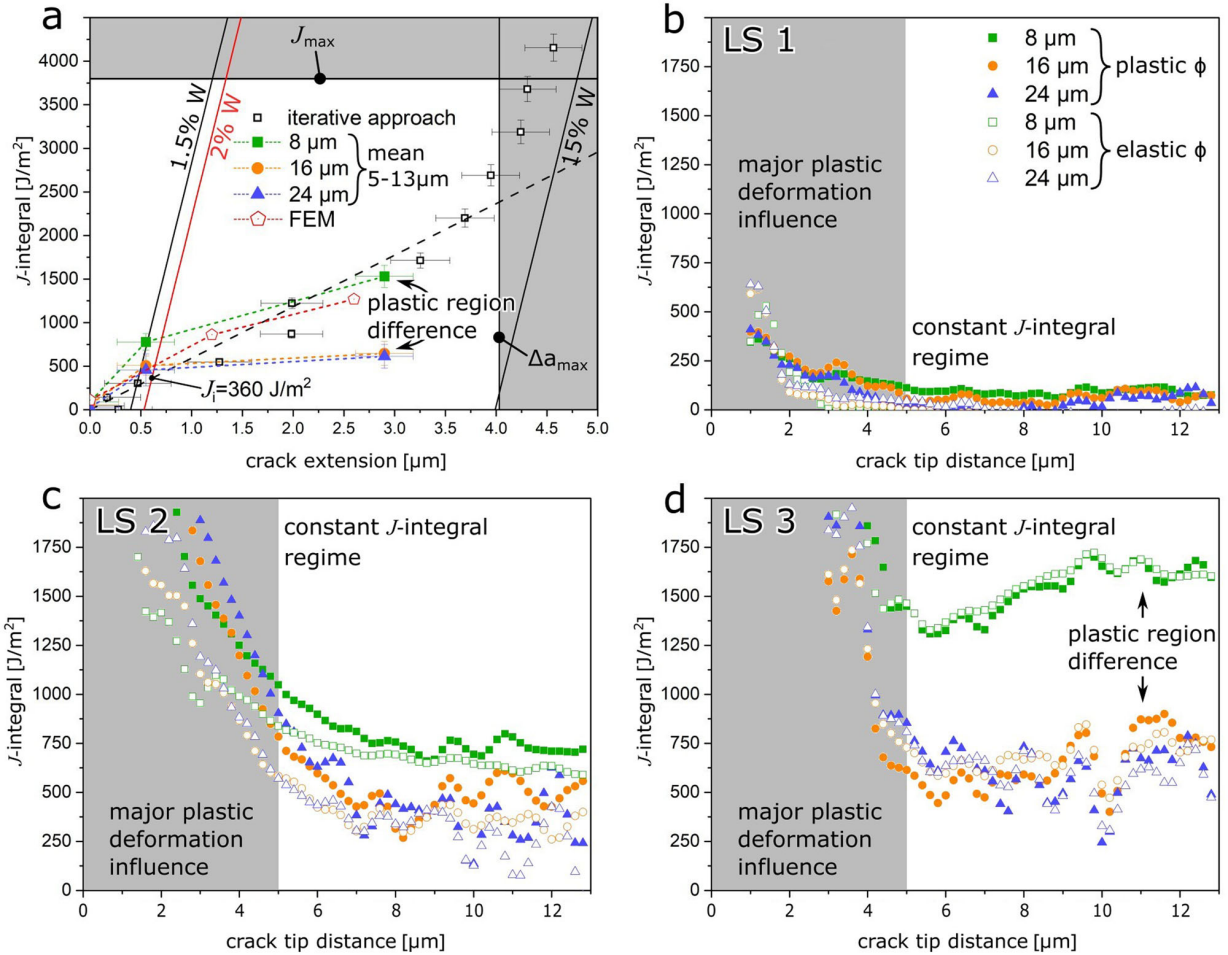
*J*-integral data obtained from the iterative and combined approaches. *J*-integral data from the iterative approach in analogy to ASTM 1820 (open black squares) in conjunction with the contour *J*-integral evaluation for LS1, LS2, and LS3 averaged from 5 to 13 µm crack tip distance as well as data from FE simulations (red pentagons). **a** The individual symbols correspond to small (8 µm: green squares), medium (16 µm: orange circles) and wide (24 µm: blue triangles) integration paths, respectively. Contour *J*-integral data as a function of distance between the crack tip and the elliptic contour are shown using plastic (filled symbols) and elastic (open symbols) strain energy density evaluation for (**b**) LS1, (**c**) LS2 and (**d**) LS3, respectively. The figure legend in (**b**) is applicable for (**c**) and (**d**) as well. The error bars in (**a**) depict the standard deviation of the *J*-integral values in the averaged region for the combined approach and uncertainty propagations from the uncorrelated geometric and mechanical input quantities for the iterative method.

The incrementally calculated *J*-integral *J*_iter_ (see Supplementary Note 7) from experimental load-displacement data is given in Fig. 6a with open black squares, exhibiting a roughly linear relationship with crack extension up to 3.5 µm, after which the slope increases significantly. This increase in slope is rather non-physical in the general *J*-integral framework and indicates a transition from crack extension to general plastic deformation^54^.

The analytical *J*-integral values enumerated from the combined SEM-DIC strain data and the CSnanoXRD stress data, *J*_comb_ (Eq. 4), are shown for each loadstep in Fig. 6b–d, respectively. The data are depicted for each ellipsoidal contour width (8 µm: green squares, 16 µm; orange circles, 24 µm; purple triangles) and different strain energy density calculation (plastic strain: filled symbols, elastic stress: open symbols) with respect to contour distance from the crack tip as schematically shown in Fig. 1b. For the mainly elastic case at LS1 the *J*-integral data slightly increases for contours very close to the crack tip, transitioning to a constant regime between 3–4 µm crack tip distance. In this case the elastically calculated data are lower than the plastically calculated data and even lead to unphysical negative *J*-integral values (minimum −57 ± 25 J ∙ m^−2^ for the 8 µm contour). However, considering the significant scatter and small absolute values a general agreement between plastic and elastic evaluation can still be stated. Furthermore, all contour widths agree amongst themselves, suggesting validity of the calculation and a path independence of the *J*-integral given a sufficiently large contour around the crack tip. For evaluation purposes the average plastically calculated values at a path-independence threshold of 5 µm crack tip distance are summarized in Table 1. LS2 (Fig. 6c) shows a strong decrease of *J*-integral with contour distance and a higher stable value for the 8 µm width compared to the 16 µm and 24 µm contours (Fig. 6c). However, purely elastic and plastic strain energy density calculations depict an overall agreement regarding the data trends. The maximum discrepancy of the average *J*-integral in the 5–13 µm range between the evaluation schemes is 131 J ∙ m^−^^2^, with the elastic evaluation generally lower for all contours. LS3 showed over proportionally high *J*-integral values up to 4000 J ∙ m^−2^ for the closest crack tip contours (between 0.5 and 2.8 µm, not shown due to scaling), which decrease rapidly with increasing contour - crack tip distance, followed by a nearly stable *J*-integral regime within a high scatter bound in the 5–13 µm range (Fig. 6d). The smallest contour width (8 µm: green squares) exhibits a minimum of *J*_comb_ ≈ 1300 J ∙ m^−^^2^ around 6 µm crack tip distance, with a slight increase towards a stable *J*-integral plateau with an average of 1530 ± 126 J ∙ m^−^^2^. In comparison, the 16 µm and 24 µm contours average at 645 ± 138 J ∙ m^−2^ and 612 ± 136 J ∙ m^−2^, respectively. This suggests a strong path dependence of the *J*-integral within the highly plastically deformed region around the crack tip. General agreement between elastic and plastic evaluation schemes is again evident, but the trend for a lower elastic evaluation is not apparent anymore. Contrarily to LS1 and LS2, the average elastically evaluated 8 µm contour width *J*-integral is even slightly higher than the plastically evaluated one, being 1554 ± 114 J ∙ m^−^^2^. While this difference is negligible in comparison to the scatter, it provides another validity check for the evaluation scheme, as both calculations follow a completely different route but end up at almost equal results.

**Table 1:** Summary of *K* and *J* and experimental data

			LS1				LS2				LS3			
SEM			iter		geom		iter				iter			
	*K*	MPa m^0.5^	5.6 ± 0.2		5.0 ± 0.1		11.0 ± 0.6				19.4 ± 1.0			
	*J*	J ∙ m^−2^	142 ± 9		114 ± 5		548 ± 31				1713 ± 87			
	*r*_p_	µm	0.3 ± 0.0		0.2 ± 0.0		1.2 ± 0.1				3.6 ± 0.2			
CSnanoXRD			COS		geom		COS				COS			
	*K*	MPa m^0.5^	4.3 ± 0.1		4.5 ± 0.2		11.5 ± 0.3				15.3 ± 0.5			
	*J*	J ∙ m^−2^	91 ± 5		92 ± 15		604 ± 16				1069 ± 35			
	*r*_p_	µm	0.2 ± 0.0		0.2 ± 0.0		1.3 ± 0.1				2.2 ± 0.1			
	*r*_p_ (T ≥ 2)	µm	–				1.0				2.9			
FE-model			Stress field		Average		Stress field		Average		Stress field		Average	
	*K*_*I*_	MPa m^0.5^	4.5		4.9		13.8		13.7		19.4		16.7	
	*K*_*II*_	MPa m^0.5^	0.3		–		0.8		–		1.5		–	
	*J*	J ∙ m^−2^	94		111		866		860		1715		1268	
	*r*_p_	µm	0.2		0.3		1.8		1.8		3.6		2.7	
Combined analysis			8 µm	16 µm		24 µm	8 µm	16 µm		24 µm	8 µm	16 µm		24 µm
	*K*	MPa m^0.5^	–	–		2.2 ± 4.7	–	–		10.0 ± 4.1	–	–		11.6 ± 2.6
	*J*	J ∙ m^−2^	91 ± 15	61 ± 23		22 ± 47	777 ± 94	507 ± 109		456 ± 186	1530 ± 126	645 ± 138		612 ± 136
	*r*_p_	µm	–	–		0.0 ± 0.1	–	–		1.0 ± 0.4	–	–		1.3 ± 0.3

Values obtained from the SEM, CSnanoXRD and modelled cantilevers individually, as well as the combined values for the three contours (8, 16, and 24 µm) according to the evaluation scheme of Supplementary Note 7 and the Methods Section.

For comparison, the average plastically evaluated *J*-integral data of all three loadsteps are depicted in Fig. 6a for each contour width, respectively. The crack extension data was taken from the CSnanoXRD-SAXSM images (*cf*. Figs. 1c and 2c, Supplementary Note 4). The intermediate (16 µm: orange circles) and largest (24 µm: purple triangles) contour widths are in excellent agreement, suggesting actual path independence of the *J*-integral along these contours, while the smallest contour (8 µm: green squares) leads to higher values already at LS2, and more than double the *J*-integral value in the fully plastic case of LS3. In comparison to the incrementally calculated values, all contour *J*-integral values are lower, with only the smallest contour calculation remaining in closer proximity.

## Discussion

In contrast to prior approaches^11,12,18,55^, the significant advantage of the present work is that for the first time stress and strain results retrieved from the two similarly deformed cantilevers of the same material were combined to evaluate the local *J*-integral along contours in front of the crack tip, which will be discussed in detail hereafter. Full details regarding the agreement between the individual cantilever bending experiments are given in Supplementary Note 2. The following discussion will be divided into two parts, first the transition from linear elastic theory to general yielding will be laid out, while second the validity and the breakdown regimes of the *J*-integral description will be considered.

To facilitate discussion, various approaches for *K-* or *J-*evaluation schemes will be compared, considering mode I loading (Supplementary Note 7), which is appropriate given the K_II_/K_I_ ratios evaluated from the FE-model range between 0.058 and 0.073 (Table 1). To circumvent confusion, subscripts will be used to denote the combined stress-strain map contour approach (comb), the iterative sequential unloading approach (iter), the classical linear elastic calculation utilizing load data and specimen geometry (geom), and fitting of the linear COS behaviour in front of the crack tip (COS), respectively. Additionally, the PZ sizes after the respective *K* values as well as measured from the triaxiality maps (*T* > 2, Fig. 4k,l) straight in front of the crack tip^8^. For comparison, all results discussed are summarized in Table 1. After loading the CSnanoXRD cantilever to ~20 mN (LS1), formation of the so-called linear elastic *K*-field around the crack tip is evident, indicated by the negative 0.5 slope in Fig. 4a, surrounding a PZ, extending less than 500 nm. Therefore, no crack extension should be present, and the PZ is likely to contain only a minor amount of plasticity. Utilizing the calculated *J*_comb_ = 22 J ∙ m^−^^2^ (largest contour, plastic evaluation scheme) for LS1 within the HRR framework would lead to COS trends following the dotted line (Fig. 4a), which does not agree with the measured stresses and provides additional evidence for predominantly elastic loading. Reversing the argument and using the COS data within the linear elastic *K*-framework yields *K*_COS_ = 4.3 ± 0.1 MPa·m^0.5^. This is in astonishing agreement with simple *K*-evaluation using the load and geometry data (see Supplementary Note 7)^56^ during the CSnanoXRD experiment at *K*_geom_ = 4.5 ± 0.2 MPa·m^0.5^. Similarly, using the corresponding load/geometry data of the SEM cantilever, yields *K*_geom_ = 5.0 ± 0.1 MPa m^0.5^, which is slightly higher than for the CSnanoXRD specimen. This discrepancy between the geometric calculations is a result of slight differences in the cantilever geometries. The iterative calculation based on the load-displacement data yields *K* = 5.6 ± 0.2 MPa m^0.5^, which is even higher and suggests that some minor amount of plastic deformation is already incorporated into the specimen.

However, this difference clearly highlights the main drawback of the iterative approach: it is not capable of distinguishing between plasticity at the crack tip or elsewhere, e.g. the indenter tip–cantilever contact or the beam base, and can therefore always lead to overestimation based on experimental conditions as detailed in^57,58^ and summarized in Supplementary Note 7. The difference between the individual *J*_comb_ evaluations (largest contour 22 J ∙ m^−^^2^, smallest contour 91 J ∙ m^−2^) is likely a result of the slight discrepancies between local spots on the two different specimens, as well as positioning uncertainties between the stress and strain fields, respectively. However, in comparison to the *J* magnitudes during further loadsteps (up to 1500 J ∙ m^−2^), these variations seem minor. As the values are in good agreement with the *K* estimates and the calculated PZ radii *r*_p_ (Table 1) are considerably small in comparison to the overall specimen geometry (max. 0.3 µm), it can be stated that linear elasticity is the governing behaviour during LS1.

After loading to 45 mN the stress slope follows the HRR model and the difference between COS and GDS agrees with this concept (Fig. 4b). In detail, the evaluated *σ*_yy_ and *σ*_zz_ distributions calculated from *J*_comb, LS2_ reach 62% of the stress magnitude of the plane strain assumption^59^. A linear regression between tabulated plane stress and plane strain constants^59^ would yield at least ~36% plane strain state in the sample. This contrasts the *σ*_xx_ (Supplementary Note 4), PST (Fig. 4e, h) and the *T* (Fig. 4k) data, which all support major plane strain state for large parts of the sample. Possible reasons for these differences may be identified in some degree of mechanical settling, where a certain force (around 10–15% at LS2) is diminished at a given static displacement (Supplementary Note 2). However, independent of the actual percentage of the 2D plane strain assumptions, the results favourably agree with the HRR model in the PZ and depict an evident transition from the linear-elastic to an elastic-plastic case. Similarly, the equivalent strain decreases with an *r*^−0.98^ slope at crack tip distances larger than 1.5 µm (Fig. 3), further supporting the HRR-type deformation case. Given the fact that LS2 agrees favourably with rather simplified theoretical predictions underlines the fact that the nanocrystalline HEA can indeed be considered to undergo quasi-ideal isotropic deformation up to this loading stage, following a Ramberg-Osgood type hardening law (Fig. 4b).

Further loading increases the strain accumulated in front of the crack tip (Fig. 3, Supplementary Note 3), while simultaneously the stress significantly diminishes (Fig. 4c, Supplementary Note 4). Especially the triaxiality (Fig. 4l) and the normal stress distributions in front of the crack tip (Fig. 4c) underline that the crack tip singularity HRR-field calculated from *J*_comb, LS3_ = 612 J ∙ m^−^^2^ is not valid anymore, since both COS and GDS do not reach the values calculated from the HRR model. While this stress reduction effect might point to void formation at the crack tip and individual large voids coalescent with the crack surface were found by ex situ SEM analysis, the lack of an increased signal in the SAXSM data ahead of the crack (*cf*. Fig. 2c and Supplementary Note 4) suggests only limited increase in free surfaces, i.e., voids. Though the reduction of the individual stress components towards the crack tip (Fig. 4c) may be related to crack tip blunting and is also well recreated by the FE-model (Supplementary Fig. S14f). Since at LS3 the FE model follows Ramberg-Osgood hardening, further increase of *σ*_von Mises_ towards the crack tip is observed (Fig. 5i). This is in stark contrast to the experimental data, where a decrease of *σ*_von Mises_ towards the crack tip at LS3 was evaluated (Fig. 5i). Thus, in agreement with the micromechanical data of the exact same HEA^24^ (Supplementary Note 1) the observed decrease of *σ*_von Mises_ might rather be resultant of the unconventional true strain-softening. Conversely, true strain softening^47,60,61^ is regularly observed in nanocrystalline metals processed by severe plastic deformation and was also found for this material^24^ (Supplementary Note 1). According to literature, strain softening is related to (i) easier dislocation nucleation due to local residual stress fields of remaining dislocation cores at grain boundaries^62^, (ii) a very large amount and area fraction of high-angle grain boundaries acting as dislocation sinks^47^ or to (iii) slight grain growth^63^. Here, the latter two possibilities concur with the observed decrease of the FWHM with ongoing deformation (Fig. 2d and Supplementary Note 4), further strengthening the argument for true strain-softening. The immediate consequence is that the *J*_comb, LS3_ calculated along the innermost path with a diameter of 8 µm settles at a higher *J* value of 1760 J ∙ m^−2^ and thus shows a path dependency even outside the closest crack tip vicinity. Additionally. excess plastic deformation is indicated by the rapidly increasing *J*_iter_ evaluated from sequential unloading (Fig. 6a).

Finally, *J*_comb_ settles only outside the crack tip-induced stress/strain fields (compare Figs. 2–4 with Fig. 5), yet yields comparable results for *J* as in LS2. Given that the experimental stress and strain data suggest general yielding at LS3, it is obvious that *J*_comb_ becomes path-dependent, as the stress-strain fields do not fulfil the requirements for the *J*-integral approach, with a minimum contour of 16 µm width at a crack tip distance of 4 µm. The resulting implications of validity and breakdown of the *J*-integral description in fracture mechanics will be discussed next.

Computing the *J*-integral from theoretical elastic and elastic-plastic stress and strain models (i.e., the *K*- and the HRR-fields, respectively) should generally yield a *J*_comb_ value independent of the integration path radius *r*. To date, only finite element simulations have been used to investigate the near crack tip vicinity and study any potential breakdown of path-independence of the *J*-integral^64,65^. However, these simulations show a decrease in *J* towards the crack tip, opposite to the experimentally observed increase presented here (*cf*. Fig. 6b–d). Possible experimental influences leading to such a behaviour would include (i) an imprecise positioning of the notch tips in the CSnanoXRD or SEM experiment, (ii) inadequate elastic constants for the stress calculation and (iii) deviations of the strain due to different strain/stress states at the surface compared to the bulk. However, the stress data shown in Figs. 2 and 4 and the supporting information fits very well to the elastic (${COS}\propto {r}^{-0.5}$) and elastic-plastic (${COS}\propto {r}^{-0.02}$) models established in literature^6–8^ for LS1 and LS2, respectively. This suggests general validity of the results and therefore only negligible errors regarding (i) crack tip position, while (ii) different elastic constants would shift the stress state. However, the bending stress component far off the notch in LS1 is in good agreement with geometrical considerations, while the σ_yy_ far off the notch is in excellent agreement with the elastic-plastic FE model for both LS1 and LS2, indicating rather adequate elastic constants (Supplementary Notes 4 and 7). Finally, the difference between plane-strain and plane-stress state cases in the *K* and HRR crack tip models only results in different pre-factors (different offsets in the double logarithmic plots), but analogous exponents (similar slopes in double logarithmic plots), which would lead to a constant shift in *J*-integral, again independent of crack tip distance.

Therefore, the only reasonable explanation for such an increase in *J*_comb_ towards the crack tip is a true strain deviating from the models (*ε* not proportional to ${r}^{-0.5}$ or ${r}^{-0.98}$ for linear elastic and elastic-plastic loading, respectively (Fig. 3)). To regard this trend in a rigorous mathematical context, we argue that both terms within the *J*-integral (Eq. 4) are proportional to stress-strain products, so estimating a circular contour $(\varphi =[{{\mathrm{0,360}}}^{\circ}\, ])$ around a crack tip in a Ramberg-Osgood type hardening material (HRR field), yields a proportionality as:

1 $$J\propto {\int }_{\varGamma }\sigma \cdot \varepsilon \cdot {r}\cdot{d}\varphi \propto {\int }_{\varGamma }{r}^{-\frac{1}{n+1}}\cdot {r}^{-\frac{n}{n+1}}\cdot {r}\cdot{d}\varphi \propto {\int }_{\varGamma }\frac{1}{r}\cdot r\cdot d\varphi$$

Therefore, no dependence with crack tip distance *r* should be present. However, if the strain was to increase with a disproportionally larger magnitude towards the crack tip, this estimation would not hold anymore, and an increase of *J* in regions of higher strain will be resultant.

This behaviour is verified by the strong discrepancy in the different contour data in Fig. 6a and d, where in the case of the narrowest contour (8 µm) part of the integration will always include over-proportional increased strain, analogous to Fig. 3, where the slope of LS3 (in the 4 µm crack tip vicinity) is evidently higher than the ${r}^{-0.98}$ proportionality. Furthermore, as shown in Fig. 4c, at LS3 neither *K* nor HRR crack tip stress fields are well represented by the stress data, suggesting a case of general yielding^1,6–8^. Thus, in this configuration the *J*-integral formulation is not valid once the integration contour is within the highly plastically deformed region around the crack tip. Additionally, the decreasing triaxiality towards the crack tip indicates that the stress state favours plastic deformation over void formation and fracture. The reason for this behaviour lies in the initial material properties of the nanocrystalline HEA alloy, which is saturated with defects before loading due to the HPT processing. This leads to defect removal during loading and indicate a true-strain softening material, supported by the FWHM microscopy data evaluated from the CSnanoXRD experiment (Supplementary Note 4) and the uniaxial stress-strain data^24^, where the true stress decreases at further (true) strain accumulation. Strain softening is regularly found in ultra-fine grained and nanocrystalline materials^47,60,61^ and related to (i) easier dislocation nucleation due to local residual stress fields of remaining dislocation cores at grain boundaries^62^, a very large area of high-angle grain boundaries acting as dislocation sinks^47^ or (iii) slight grain growth^63^. While for the first possibility, strains of 2^nd^ order would rise the FWHM, the latter two possibilities concur with a decrease of the FWHM with ongoing deformation (Fig. 2d and Supplementary Note 4). In this case, further accumulation of plasticity would lead to defect annihilation at the crack tip, subsequent softening and thus an over-proportional increase in strain magnitude upon loading.

In comparison to the common iterative approach often conducted in micromechanical testing (Fig. 6a,^57,66^) it is evident that both the *J*-integral magnitude as well as the crack extension are considerably overestimated in the later part of the experiment (LS3). This can be attributed to plasticity, which is not solely governed by the crack tip, e.g. contains also deformation at the cantilever base, the compressive fibre at the ligament, or the wedge tip-cantilever contact. All of these factors can influence the stiffness and therefore the crack extension measurement as well as the load-displacement response, which directly relates to the calculated *J*_iter_. However, considering LS2 where the HRR field description still holds (Fig. 4b) and the *J*-integral acts as valid crack tip loading parameter, the agreement with the iterative approach is astounding (Table 1). However, this agreement is restricted to identifying the onset of crack growth, while taking the same load-displacement data as for the iterative approach yields significantly overestimated crack growth and *J*-integral values by the iterative approach compared to the combined data (Fig. 6a). Consequently, the apparent restriction in the present case is a sufficient crack tip integration distance of ~5 µm at a lateral expansion of 16 µm (Fig. 6c and Table 1), but in general, this depends on the material, specimen size and external loading parameters.

Therefore, the comparatively simple iterative approach can still be used to determine a conservative scalar fracture characteristic for the onset of crack extension, even for non-conventional systems, such as highly plastically deforming or even strain softening materials alike the nanocrystalline HEA studied here. Nevertheless, the iterative approach fails completely to describe the aspects of plastic failure in the HEA, which includes pore formation and coalescence with the crack tip (Supplementary Note 2) and the effects of strain softening (Supplementary Note 1) resulting in path dependence of the *J*-integral. Subsequently, the iterative *J*-integral is overestimated by at least a factor of 3, while crack growth is exaggerated up to 50% (Fig. 6). In following, if a detailed investigation on the crack growth resistance is the aim of any study, it is evident that the iterative approach does not deliver reliable results and more elaborate experimental methods, such as introduced herein, are necessary for any rigorous assessment. Additionally, with ongoing miniaturization of modern materials systems, individual mechanically relevant constituents may not extend to the critical dimensions necessary for a valid *J*-integral description as shown in this study, while testing conditions probably introduce plasticity elsewhere in the sample. Furthermore, the here presented results hold true not only for the case of the nanocrystalline HEA presented here for the broader case of nanocrystalline materials (with the capability for plastic deformation), most of which show softening during deformation^47,60,61^. Finally, the findings of this study hold even further truth in the case of modern structural (and functional) materials or technologically relevant heterogeneous devices, which may show distinctive mechanical behaviour not covered by the Ramberg-Osgood law^5^, such as secondary phases^33^, dislocation multiplicity^26^ or dynamic competition of screw and edge dislocations^28^.

Given the experimental progress demonstrated in this study, future possibilities of combined stress and strain evaluation in front of crack tips include technologically highly relevant semi-ductile materials such as high-strength steels^67–69^ or Ti alloys^70,71^ which can exhibit brittle fracture after certain amount of plastic deformation. The unique possibilities of the combined in situ SEM and CSnanoXRD approach will further enhance understanding of the partitioning of stresses and strains and their association with fracture in ductile or semi-ductile materials, even across interfaces or in gradient materials. Further experimental improvements will be enabled by much higher scan rates during the in situ CSnanoXRD experiment at 4^th^ generation synchrotron radiation facilities^72,73^ and the application of multi-layer Laue lenses (MLLs)^74,75^. While the former allows for faster data generation and thus more investigated loadsteps, the latter enables focussing the X-ray beam below 100 nm in diameter, thereby further enhancing the spatial resolution of the X-ray experiment^74^. While the correlated SEM experiments enabled the strain determination in the present investigation, the caveat of having similar and quasi-isotropic, but still two individual, rather than one unique specimen, remains. However, the feature-based strain mapping as presented herein is not limited to SEM imaging. Thus, using single experiments and advanced image formation capabilities (SAXS and FWHM microscopy) during CSnanoXRD experiments will remove any potential doubts regarding differences between stress and strain data in the future.

## Conclusions

This work presents the first experimental evaluation of the elastic-plastic fracture parameter *J*_comb_ in front of a crack tip with previously unseen deep sub-micron resolution. The unique methodology developed here allowed for the separate analysis of strain and stress in a nanocrystalline FeCrMnNiCo HEA alloy. The experimental results detail the transition from linear elastic loading via elastic-plastic deformation to general yielding, in agreement with respective theoretical concepts. Thereby, we demonstrate that even in the case of a valid HRR field the path independence of the *J*-integral is not given in a rather large crack tip vicinity of several microns, especially for the strain-softening nature of the nanocrystalline material studied herein. In the case of general yielding, the *J*-integral obtained by the sequential unloading method significantly deviates from the one obtained from actual stress and strain fields, which is related to true strain softening of the nanocrystalline HEA. In turn, the *J*-integral becomes also path dependent, even outside the crack tip vicinity. Consequently, the *J*-integral is no longer a valid description of the crack growth resistance beyond the onset of fracture in state-of-the-art elastic-plastic materials both when deviating from a simplistic Ramberg-Osgood hardening behaviour and at the microscale. These findings underline the importance of refined fracture mechanical concepts and experimental analysis schemes considering crack growth resistance for assessing the damage tolerance of advanced materials on a local scale.

## Materials and methods

### Material preparation

The material used in this study is an equiatomic five-component CoCrFeMnNi HEA, commonly known as the Cantor alloy^36,37^, which was processed by HPT under the same technical parameters (pressure, strain-rate and temperature) as described by Schuh et al.^37^ to achieve a nanocrystalline microstructure with a grain size of 50 nm. A wedge-shaped specimen was prepared from the HPT disk by grinding and careful polishing to a final thickness of ~40 µm leading to a specimen orientation with the crack propagation direction in the radial direction and the crack plane normal pointing in the tangential direction.

### Femto-second laser and focused ion beam sample preparation

An Auriga laser^76^ system (Carl Zeiss AG, Oberkochen, Germany), combining focused ion beam (FIB) milling and femtosecond laser ablation (Origami 10 XP, Onefive GmbH, Regensdorf, Switzerland) was used to fabricate the notched freestanding cantilevers of *W* × *B* = 26.2–26.5 µm × 28–29 µm, with *L* chosen in such way that a similar nominal stress intensity is applied for both cantilevers. The initial notch depth was ~9 µm, to obtain single defect-controlled specimens with a/W ~ 0.3 as common in small-scale fracture investigations. Full details about the specimen fabrication routine can be found in Supplementary Note 6.

### Scanning electron microscopy imaging

Cantilevers for in situ SEM and CSnanoXRD experiments were imaged prior and after the testing in a field emission SEM (ZEISS LEO1525, Carl Zeiss AG, Oberkochen, Germany) using the in-lens secondary electron detector at an acceleration voltage of 5 kV and an aperture size of 30 µm.

### In situ SEM-DIC experiment

A cantilever specimen was tested in situ in a field emission SEM (DSM982, Carl Zeiss AG, Oberkochen, Germany) utilizing an UNAT-SEM 1 microindentation device (ASMEC GmbH, Dresden, Germany) equipped with a conductive diamond wedge (Synton MDP, Nidau, Switzerland). The cantilever bending experiment was conducted in displacement-controlled mode with a loading rate of 50 nm/s and sequential unloading steps at every for every 2 µm displacement. To assure a quasi-continuous acquisition of images, the SEM was adjusted to a scan speed requiring 660 ms per frame, which provided a good trade-off between image quality in terms of signal-to-noise and temporal resolution^24^. The iterative *J*-integral is calculated as presented in ref. ^57^. As the evaluation of a proper crack growth initiation toughness at the microscale cannot be directly evaluated using macroscopic standards (e.g. ASTM1820^77^), the 2%*W* (ligament width) translation criterion, proposed by Pippan et al. ^58^, was used. This is shown in Fig. 6a, where blunting lines with slopes of 2*σ*_y_ = 4710 MPa^24^ are drawn for 1.5%, 2%, and 15% *W* offsets, respectively. In macroscopic experiments, the data between the 1.5% and 15% *W* offsets would count as valid for a fitting procedure, whereas the intersection of the fitted line with the 2% W line indicates the crack growth initiation toughness. Furthermore, additional invalidity criteria for *J*-integral testing are the maximum *J*-integral capacity ${J}_{\max }=(W-{a}_{0})\cdot {\sigma }_{{{{\rm{y}}}}}/10=3796\,{{{\rm{J}}}}\cdot {{{{\rm{m}}}}}^{-2}$ and maximum crack extension capacity ${\varDelta a}_{\max }=0.25\cdot \left(W-{a}_{0}\right)=4.03\,{{{\rm{\mu m}}}}$. Using these criteria as shown in Fig. 6a with a linear fit of the data results in a conditional crack growth initiation toughness *J*_i_ = 360 J ∙ m^−2^. The determination of the 2D surface Green-Lagrange strain tensor by point feature tracking is conducted on quasi-continuous SEM images during the experiment, as described in ref. ^24^. Additionally, the strain parallel to the crack tip direction *ε*_xx_ was estimated based on volumetric invariance of the individual quadrilaterals to obtain a quasi-3D representation of the surface strain state. The measured displacement fields are processed using a novel smoothing algorithm based on total variational regularization with a regularization strength of 10^−^^2^ (*cf*. Supplementary Note 8 and Supplementary Fig. S18). To assess the stochastic scatter of this methodology, the strain field in crack opening direction *ε*_yy_ was evaluated from the first to the second frame of the SEM-DIC experiment, which is still out of contact of the indenter. All 3311 evaluation points of this frame were plotted as histogram and used to determine a purely noise dominated normal distribution of *ε*_yy_. The mean and standard deviation of this distribution were calculated as −3.1 × 10^−5^ ± 2.9 × 10^−^^4^, showcasing the very good agreement with the unstrained condition, i.e. *ε*_yy_ = 0 and a statistic estimate on the average strain value errors of approx. 3 × 10^−^^4^.

### In situ CSnanoXRD experiment

The CSnanoXRD experiments^78,79^ (Fig. 1a, c) were performed at beamline ID13 of the European Synchrotron (ESRF) in Grenoble, France^74^ using a dedicated indenter setup developed for in situ indentation experiments^80^. Compound refractive lenses^81^ were used to focus the X-ray beam with a photon energy of 15.2 keV to a spot size of ~150 nm in diameter and a focal depth of ~50 µm. After aligning the sample perpendicular to the incident X-ray beam (*cf*. supporting information), the cantilever was incrementally loaded to 22, 45, and 36 mN (*cf*. Supplementary Fig. S1), denoted as loadsteps LS1, LS2, and LS3, respectively, and three areas of interest, sized 30 × 35 µm², 14 × 10 µm² and 6 × 5 µm² were characterized in detail by mesh scanning the sample along the *y-* and *z-* direction in 500, 200, and 100 nm steps, respectively, for each of the three loadsteps. Note that the extent of the X-ray gauge volume is equal to the cantilever width B along the *x*-direction. During the in situ CSnanoXRD experiment 8933 2D diffractograms per loadstep (LS1–LS3) were recorded using an Eiger X 4 M (hybrid photon counting) HPC detector. Additionally, before and after the experiment, further denoted as LS0 and LS4, respectively, the largest areas of interest of 30 × 35 µm² size were scanned along the *y- and z-* direction with a decreased step size of 200 nm, resulting in 26576 recorded 2D diffractograms each. The, along the *x*-direction volume-averaged, 2D diffraction signal was recorded by a Dectris Eiger X 4 M HPC detector at each measurement position using an acquisition time of 50 ms at a sample-to-detector distance of 132.47 mm.

The scattered signal around the beam stop, i.e., small-angle X-ray scattering (SAXS) (Fig. 1c) originates primarily from electron density variations, such as alternation of materials, presence of grain boundaries, interfaces, cracks, precipitates and pores with sizes of ~$\,\lambda /\theta$ where $\lambda$ represents the X-ray wavelength and $\theta$ is the Bragg angle^46,82^. Using so-called SAXS microscopy (SAXSM^46^), micrographs primarily sensitive to the intercrystalline defect density were collated to quantitatively evaluate the volumetric crack length averaged over the cantilever thickness *B* for the individual loadsteps. The arithmetically averaged direction-dependent FWHM^19^ were collated to a micrograph for each load step, yielding a vital qualitative parameter to determine the defect accumulation around the crack in the in situ experiment.

The unstressed lattice constant of the face-centered cubic (fcc) HEA was determined from diffraction data near the surface of the cantilever before loading (LS0), considering the stress-free out-of-plane (*z*) orientation and found to be ${a}_{0}=0.35928$ nm, according with literature^50^. The evaluation of the 3D stress distributions (*i.e*., the full stress tensor) was performed using the approach from refs. ^18,83^. X-ray elastic constants were adopted from literature^84^. The evaluation of the 3D stress tensor was supported by using the diffracted intensities from the 111 and 200 Debye-Scherrer rings, as they have highly different X-ray elastic constants, a consequence of the high crystallographic anisotropy represented by a Zener ratio of *Z* = 4.2^84,85^. Therefore, the individual ring’s changes $2\theta \left(\delta \right)$ pursuant to the stress tensor are significantly different (*cf*. supporting information), which in turn allows to retrieve ${\sigma }_{{{{\rm{xx}}}}}$. Further details on evaluation and experimental constraints can be found in Supplementary Note 9.

### Calculation of stress indicators for data interpretation

As a verification for the ${\sigma }_{{{{\rm{xx}}}}}\left(y,z\right)$ values obtained, a test for the plane strain condition was adopted as follows

2 $${PST}=\,\frac{{\sigma }_{{{{\rm{xx}}}}}\left(y,z\right)}{{\sigma }_{{{{\rm{yy}}}}}\left(y,z\right)+{\sigma }_{{{{\rm{zz}}}}}\left(y,z\right)},$$

where the PST is the ratio between the normal stress component along the incident beam, ${\sigma }_{{{{\rm{xx}}}}}\left(y,z\right)$ divided by the sum of the normal stress components (${\sigma }_{{{{\rm{yy}}}}}\left(y,z\right)$, ${\sigma }_{{{{\rm{zz}}}}}\left(y,z\right)$) perpendicular to the incident X-ray beam (*cf*. Fig. 1). For purely elastic loading in front of the crack tip, the PST value should coincide with the Poisson’s ratio of the Cantor alloy, which is *υ*_exp_ = 0.253 ± 0.017 as experimentally obtained in^24^. During further loading and concomitant plasticity in front of the crack tip, the PST value should increase towards 0.5, which represents incompressible volume conserving deformation, full plastification of the material^52,53^, while values above 0.5 indicate a deviation from conserved volume, e.g. void formation^86^.

The triaxiality ratio $T\left(y,z\right)$^51^ was calculated by dividing the hydrostatic component of the stress tensor ${\sigma }_{{{{\rm{H}}}}}\left(y,z\right)$ by the von Mises stress ${\sigma }_{{{{\rm{von\; Mises}}}}}\left(y,z\right)$

3 $$T\left(y,z\right)=\,\frac{{\sigma }_{{{{\rm{H}}}}}\left(y,z\right)}{{\sigma }_{{{{\rm{von\; Mises}}}}}\left(y,z\right)},$$

where ${\sigma }_{{{{\rm{H}}}}}\left(y,z\right)=\frac{1}{3}\left({\sigma }_{{{{\rm{xx}}}}}\left(y,z\right)+{\sigma }_{{{{\rm{yy}}}}}\left(y,z\right)+{\sigma }_{{{{\rm{zz}}}}}\left(y,z\right)\right)$ is the 1^st^ invariant of the stress tensor.

### Finite element modelling

A 3D elasto-plastic Finite Element (FE) model was employed to simulate the indentation process of the pre-notched cantilever using the commercial software COMSOL Multiphysics®. The material constitutive law was modelled using a Ramberg-Osgood hardening law (Eq. S1.1) as discussed in Supplementary Note 1. The adopted plasticity formulation accounts for small plastic strains, with the yield function determined by the Von Mises stress criterion. Given the large deformation expected by this test, geometry non-linearities were accounted by a preceding cohesive zone model using the *J*_i_ of 360 J ∙ m^−2^ as the strain energy release rate. The mechanical model exploited second-order serendipity hexahedral elements with 20 nodes, having quadratic shape functions with the mesh consisting of 60216 elements

The values of ${J}_{{{{\rm{avg}}}}}$, were obtained using the dedicated algorithm available in COMSOL Multiphysics. This function computes the 2D *J*-integral at multiple cross-sections along the specimen’s thickness, generating a continuous profile of the *J*-integral. By averaging this profile across the sample’s thickness, it is then possible to obtain ${J}_{{{{\rm{avg}}}}}$. Full details of the FE-models parameters and the process are given in Supplementary Note 10.

### Calculation of strain energy density and *J*-integral

Obtaining the full local stress state from CSnanoXRD experiments as well as the full local strain state by SEM-DIC experiments enables determination of *J*-integral values based on the theoretical contour integral definition^4^, as:

4 $$J={\int }_{\varGamma }{\phi \; dy}-\vec{T}\frac{\partial \vec{u}}{\partial z}{ds}$$

where, $\phi ={\int }_{0}^{{\varepsilon }_{{{{\rm{eq}}}}}}\underline{\sigma }\,{d}\underline{\varepsilon }$ is the strain energy density, $\vec{T}=\underline{\sigma }\vec{n}$ is the surface traction vector, and $\vec{u}$ is the deformation vector field, while $\underline{\sigma }$ and $\underline{\varepsilon }$ represent the stress and strain tensors, respectively. The integral is calculated along the contour *Γ*, as shown in Fig. 1b from the lefthand-side crack flank to the righthand-side crack flank, in accordance with common literature^4^. To investigate the path-independence of the *J*-integral from the experimental data, various elliptical contours were evaluated, with the origin of each curve constant at 3 µm behind the crack tip and varying maximum eclipse points of the contour in 100 nm steps from 1 µm crack tip distance to a maximum of 13 µm crack tip distance. Furthermore, three different contour widths were chosen to study the influence of the contour path through a majority of the PZ (small) or mainly through an elastically loaded regime (wide) as depicted in Fig. 1b.

Strictly speaking, the *J*-integral evaluation is considered valid only for linear and non-linear elastic material behaviour. This means that the total work that was put into a certain volume should be considered reversible and therefore adds to the stored strain energy density. To address this assumption the strain-energy density $\phi$ was calculated following two different approaches. On the one hand, ${\phi }_{{{{\rm{elastic}}}}}$ was calculated using purely linear-elastic assumptions and only the CSnanoXRD data as:

5 $${\phi }_{{{{\rm{elastic}}}}}=\frac{1}{2}{\underline{\sigma }}\,{\underline{\underline{S}}}\, {\underline{\sigma }}^{T}$$

with $\underline{\underline{S}}$ being the isotropic compliance tensor. On the other hand, ${\phi }_{{{{\rm{plastic}}}}}$ was calculated by numerical integration of the true stress-strain data ${\sigma }_{{{{\rm{uniaxial}}}}}$, as previously determined on micro tensile experiments^24^, up to an equivalent total strain ${\varepsilon }_{{{{\rm{eq}}}}}$ as ${\phi }_{{{{\rm{plastic}}}}}={\int }_{0}^{{\varepsilon }_{{{{\rm{eq}}}}}}{\sigma }_{{{{\rm{uniaxial}}}}}\cdot d\varepsilon$, where ${\varepsilon }_{{{{\rm{eq}}}}}$ is calculated as the von-Mises equivalent strain from the SEM-DIC strain data. This enables a comparison between linear-elastic and non-linear elastic assumptions in the standard *J*-integral framework. A detailed analysis of the possible error sources during the integration of the experimental data is given in Supplementary Note 11.

## Supplementary information

Supplementary Information

Description of Additional Supplementary Files

Movie S1

Movie S2

Movie S3

Movie S4

## Data Availability

The data that support the findings of this study are available from the corresponding authors upon reasonable request.

## Code availability

The codes that support the findings of this study are available from the corresponding authors upon reasonable request.
