# Supplementary material for: Resolving the fundamentals of the J-integral concept by multi-method in situ nanoscale stress-strain mapping
Source: Commun Mater. 2025 Feb 22;6(1):35. doi: 10.1038/s43246-025-00752-z (PMC11846709; doi:10.1038/s43246-025-00752-z)
Supplement: Supplementary file 1 — Supplementary Information [file 43246_2025_752_MOESM1_ESM.pdf]

Resolving the Fundamentals of the *J*-integral Concept by Multi-Method *in situ* Nanoscale Stress-Strain Mapping

Michael Meindlhumer[1]†*, Markus Alfreider[1]†*, Noel Sheshi[2], Anton Hohenwarter[1], Juraj Todt[1], Martin Rosenthal[3]‡, Manfred Burghammer[3], Enrico Salvati[2], Jozef Keckes[1], Daniel Kiener[1]

[1]Department of Materials Science, Montanuniversität Leoben, Franz-Josef Straße 18, 8700 Leoben, Austria

[2]Polytechnic Department of Engineering and Architecture (DPIA), University of Udine, via delle Scienze 206, 33100 Udine, Italy

[3]ESRF – The European Synchrotron, Avenue de Martyrs 71, 38013 Grenoble, France

‡current address: KU Leuven, Department of Chemistry, Celestijnenlaan 200f, 3001 Leuven, Belgium

*corresponding authors, e-mail: michael.meindlhumer@unileoben.ac.at, markus.alfreider@unileoben.ac.at

†These authors contributed equally

This PDF file includes:

Supplementary Note 1: Microstructure and micromechanical properties of the nanocrystalline FeCrMnNiCo alloy 3
Figure S1: Micromechanical data of the HPT deformed Cantor alloy. 4
Supplementary Note 2: Comparability of the *in situ* CSnanoXRD and SEM experiments 5
Figure S2. Similarity of the deformed cantilevers. 5
Supplementary Note 3: Detailed strain data of individual load steps 8
Figure S3. Strain data with a total variation regularization parameter of λ=0.01 9
Supplementary Note 4: Detailed CSnanoXRD microstructure and stress analyses 10
Figure S4. Experimentally obtained azimuthal intensity distributions. 10
Figure S5. Experimentally obtained CSnanoXRD data for LS0. 12
Figure S6. Frequency distributions of the stress tensor before loading 13
Figure S7. Experimentally obtained CSnanoXRD data for LS1. 16
Figure S8. σ_{yy} stress component at y=-15 µm 17
Figure S9. Crack tip stresses in linear coordinates. 17
Figure S10. Experimentally obtained CSnanoXRD data for LS2. 20
Figure S11. Experimentally obtained CSnanoXRD data for LS3. 22
Figure S12. Experimentally obtained CSnanoXRD data for LS4. 24
Supplementary Note 5: Detailed FE analysis 25
Figure S13. FE data obtained for LS1 26
Figure S14. Comparison of experimental and modelled σ_{yy} distributions. 27
Figure S15. FE data obtained for LS2 29
Figure S16. FE data obtained for LS3 31

Supplementary Note 6: Details about the cantilever fabrication 33
Figure S17. SEM images of FIB-prepared cantilevers 33
Table S1. Geometries of the cantilevers deformed *in situ* in the SEM and during the synchrotron CSnanoXRD experiment.. 34
Supplementary Note 7: Evaluation of the iterative *J*-integral (J_{iter}).. 35
Supplementary Note 8: SEM - digital image correlation (SEM-DIC) strain analysis.. 36
Figure S18. Comparison of different smoothing algorithms 37
Figure S19. Raw strain data 38
Figure S20. Strain data with a total variation regularization parameter of λ=0.02 38
Supplementary Note 9: CSnanoXRD experiment 39
Figure S21. 2θ-δ plots in polar coordinates. 41
Supplementary Note 10: Detailed FE-analysis 42
Figure S22. Representation of the 3D model employed for the FE simulation. 42
Figure S23. Overview of the structured mesh employed in the FE model. 43
Figure S24. Crack bisector planes and crack growth obtained by the cohesive zone model. 44
Supplementary Note 11: J_{comb} integration parameter analysis.. 45
Figure S25. Integration step size analysis. 45
References.. 46
Movie S1. 50
Movie S2. 50
Movie S3. 50
Movie S4. 50

Supplementary Text
Figs. S1 to S23
Tables S1
Movies S1 to S4

Other Supplementary Materials for this manuscript include the following:
Movies S1 to S4

Supplementary Note 1: Microstructure and micromechanical properties of the nanocrystalline FeCrMnNiCo alloy

Microstructure

The microstructure of the nanocrystalline FeCrMnNiCo high-entropy alloy (HEA) processed by high pressure torsion (HPT) using the same process parameters was investigated several times by transmission electron microscopy[1–5]. The works consistently showed that the HEA exhibits rather equiaxed grains with a grain size in the order of 50 nm after processing by HPT. The presence of rather equiaxed grains together with the absence of a pronounced crystallographic texture (discussed in Suppl. Note 4) supports an isotropic deformation behavior.

Micromechanical Properties of the HEA

Numerous indentation experiments allow to assess the yield strength σ_y of the nanocrystalline HEA. Exemplary, Schuh *et al.* [1] measured a hardness of 520 $HV_{0.5}$, which suggests a yield strength of at least 1.7 GPa, while a single tensile experiment of the HPT-processed HEA revealed a yield strength ~2 GPa.

Additional nanoindentation tests yielded a hardness ranging from 6 to 6.5 GPa[3,4] after HPT-processing, which can be converted into a yield strength ranging from 2.0-2.2 GPa. However, conventional micro- and nanoindentation use self-similar pyramidal indenter tips, which induce constant strain independent of the indentation depth. To get better insight into the micromechanical behavior spherical nanoindentation and microtensile tests were carried out and published by the authors[6]. The main findings of this study are reiterated here to give additional insight into the micromechanical properties of the nanocrystalline HEA.

For comparison with theoretical assumptions as well as for FE-simulations it is necessary to simplify the mechanical response in the shape of a Ramberg-Osgood equation as:

$$\varepsilon = \frac{\sigma}{E} + 0.002\left(\frac{\sigma}{\sigma_y}\right)^n \qquad \text{(S1.1)}$$

where the representative total strain ε is a function of the occuring representative stressσ, the elastic modulus E, the yield onset σ_y and a hardening exponent n.

It is essential to mention that the presented stress-strain data in Fig. S1a, represent analytical true stress-strain values to the best of the authors abilities and within experimental constraints. Special focus was laid on the determination of true-stress values by incorporating the width reduction in the necked region (measurement W in Figs. S1b,c).

The blue data points depict data from the previous publication[6] whereas the open symbols depict data using manual geometry measurements of the same specimen (blue data, #1) as well as an additional specimen (red data, #2). While the strain values are not completely in agreement between the different measurement techniques (manual measurements underestimate the local strain accumulation), it is evident that all data shows a decrease of true stress values with increasing strain as oftentimes observed in nanocrystalline materials[7].

Furthermore, stress strain data from multiple individual spherical nanoindentation experiments (conducted after the methodology in[8]) are depicted in Fig. S1d. In contrast to tensile experiments, spherical nanoindentation experiments prescribe a majorly compressive stress state on the plastically deformed volume (Fig. S1e), which does not allow for localization through necking. Rather, the possibility for pileup formation during plastic deformation could lead to an increased load and thus an overestimation of local flow stress. As even the nanoindentation data show a softening upon deformation (inset Fig. S1d) it can be assumed that the material shows true softening upon deformation, either through easier dislocation nucleation due to local residual stress fields of remaining dislocation cores at grain boundaries[9] or through slight grain growth[10].

For theoretical considerations within the main manuscript the tensile data from[6] was used with resulting Ramberg-Osgood values of E = 205GPa, σ_y = 2355 MPa and n=50.

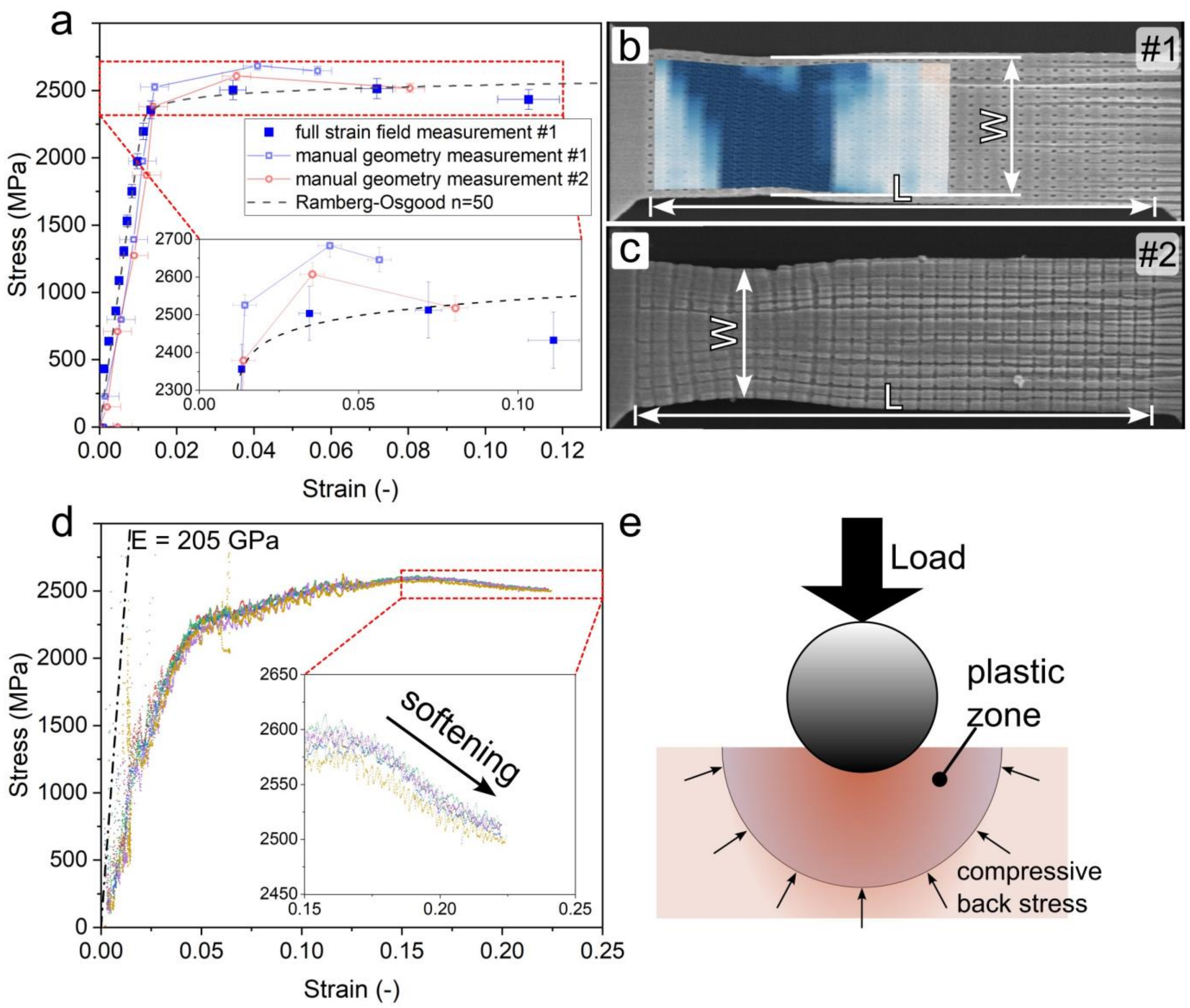

Figure S1: Micromechanical data of the HPT deformed Cantor alloy.

(a) True stress-strain curves of two individual microtensile specimens (#1,#2) evaluated manually and using the point feature tracking DIC algorithm as in the preset work (b,c) The last frames of specimens #1 and #2 before failure, respectively. (d) Spherical nanoindentation data on the same material, showing softening and (e) a schematic of the plastically deformed zone during spherical nanoindentation and the resulting compressive backstress.

Supplementary Note 2: Comparability of the *in situ* CSnanoXRD and SEM experiments

Load-displacement curves

The load-displacement curves obtained from the *in situ* SEM (Fig. 1a,b) and CSnanoXRD (Fig. 1a,c) experiments are summarized in Fig. S2a. While loading was performed continuously for the *in situ* SEM experiment, with sequential unloading steps to evaluate the iterative *J*-integral (*cf*. Methods), the *in situ* CSnanoXRD experiment was carried out stepwise and was interrupted for up to 70 min at the positions LS0-LS4 to acquire the CSnanoXRD maps, which lead to slight load relaxation at these positions. However, as can be seen in Fig. S1, the load-displacement curves overlap and match nearly perfectly up to LS2, which is a good indication for the similarity of the two *in situ* experiments.

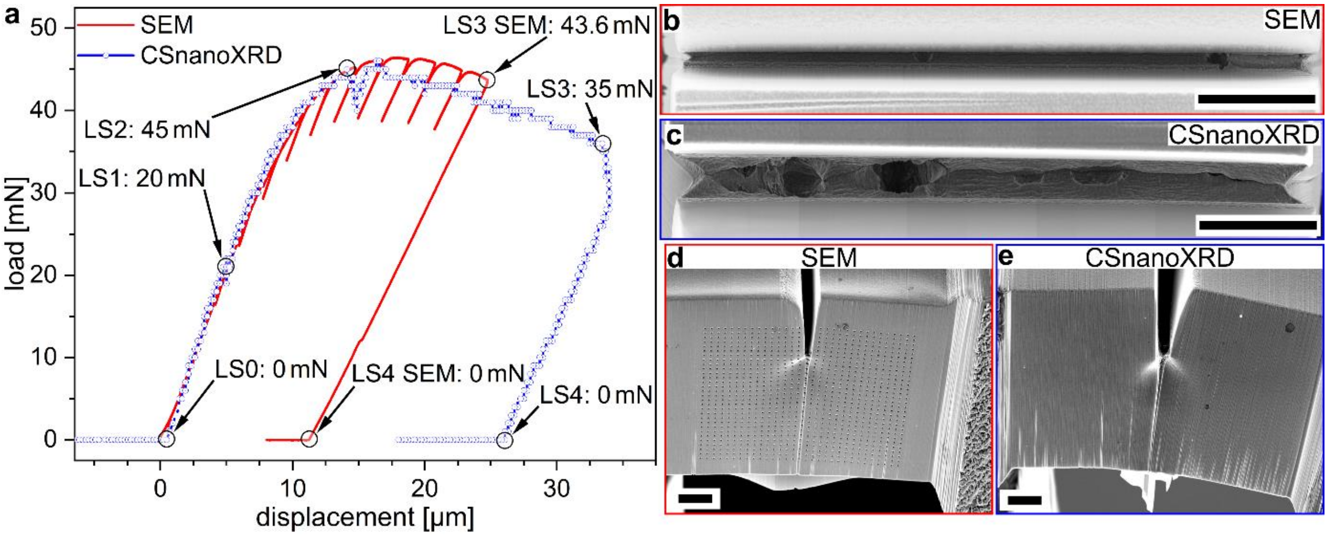

Figure S2. Similarity of the deformed cantilevers.

Load-displacement curves for the cantilevers deformed *in situ* in the SEM (red line) and during the synchrotron experiment (pointed blue line) are presented in (a). Post deformation images of the crack surface of the cantilevers deformed in SEM (b,d) and during the CSnanoXRD experiment (c,e) show the similarity of both experiments. The crack front with beginning and extensive pore formation during loading is shown in (b) and (c), respectively, while at the front face of the cantilevers a constriction implying plastic deformation in front of the crack tip was evident (arrows in (d) and (e)). All scale bars are equivalent to 5 µm.

SEM analysis of crack surfaces

SEM images of the crack front and the surface of the deformed cantilevers are presented in Fig. S2. Both samples deformed similarly in a ductile manner, as demonstrated by the formation of pores at the crack front (Fig. S2b,c). Furthermore, the evident Poisson's contraction on the outside surface of both cantilevers is equally pronounced and suggests the presence of a plane-stress state at the surface, as well as equal and isotropic deformation in both specimens (Fig. S2d,e). With the now established analogous deformation in both specimens we can combine the individual localized stress and strain information to evaluate the *J*-integral in its original form.

Similarity of the experimental setups and measurement conditions

First, in order to ensure equal stress and strain fields at the investigated loadsteps, the similarity of the samples and the applied bending stress has to be ensured. Here, given the widths *W* and thicknesses *B* of the samples, the lengths *L* were chosen in such way, that equal nominal stress intensities of ~5 $MPa \cdot m^{0.5}$ were applied at the notch tips of both cantilevers in the elastic regime (~20 mN, *cf.* Suppl. Note 6). The strong agreement between the load-displacement curves in Fig. S2a underlines similar external deformation was applied to the specimens during the CSnanoXRD and SEM experiments, respectively. The only major discrepancy is evident at LS3, where the CSnanoXRD cantilever was deformed further than the SEM cantilever. However, given the similar appearance of the crack surface after both experiments showing both a pore formation and coalescence with the crack tip as well as comparable plastic contraction at the surface, it is reasonable to assume that the stress and strain states at LS3 SEM and LS3 CSnanoXRD are comparable. This is furthermore supported by the low hardening (and even slight softening) of the nanocrystalline HEA, which leads to a rather stable flow plateau in uniaxial deformation[1,6], the stress levels at the SEM LS3 should be similar to CSnanoXRD LS3.

One further difference between SEM and CSnanoXRD experiments is that loading in SEM is sequentially interrupted by unloading events, but otherwise continuous, while loading during the CSnanoXRD experiment has to be stopped at a fixed displacement to scan the area of interest. Consequently, in the CSnanoXRD experiment a slight load drop is present due to thermal drift and relaxation of the setup, which reduces the measured stress magnitudes marginally. However, the loading rates between the fixed scan positions have been kept equal to the continuous loading rates during the *in situ* SEM experiments, to counteract any variations in deformation characteristics with varying strain rate sensitivity[4]. While the slight relaxation during CSnanoXRD scans is a minor drawback, it is clearly outweighed by the benefit of the obtained high-resolution multiaxial stress data. Here, the methodology was even enhanced further by (i) visualizing the crack growth using SAXSM (Fig. 1c, Fig. 2c), (ii) enlightening the shear band formation in the volume by FWHM microscopy (Fig. 2d, Supplementary Note 3) and (iii) obtaining a complete 3D stress tensor.

Up to now, *in situ* CSnanoXRD experiments on clamped cantilever fabricated from a CrN-Cr multilayer thin film[11] and indentation on various thin films[12–15] did not allow for the quantification of σ_{xx} due to (i) a strong crystallographic texture accompanied by a rather large crystallite size in the investigated materials and (ii) in case of Cr, only one DS ring was present on the detector in the same experimental geometry as chosen herein, which prohibits the evaluation of σ_{xx}, since in such case every change of σ_{xx} can be also interpreted as a change of a_0.

The experimental σ_{xx} distributions were further rigorously validated by the introduced *PST* ratio (Fig. 4d-f), which yielded ~0.25 during elastic loading close to the crack tip, in excellent agreement with the experimentally derived Poisson's ratio of 0.253 for the same material[6]. During crack tip blunting the *PST* value rose towards 0.5, which suggests ideal plastic deformation[16], further supporting the validity of the experimental σ_{xx} distributions. Furthermore, the σ_{xx} and *PST* distributions (Fig. 4d-f) support the presence of a plane strain state in front of the crack tip, in agreement with Narasimhan and Rosakis[17], which revealed plane strain in the vicinity of the crack tip with decreasing σ_{xx} with increasing distance from the crack tip.

However, the clearest advantage of this works is that, in contrast to prior publications[11,18–20], the stress and strain results retrieved from the two similarly deformed cantilevers of the same material were combined to evaluate the local *J*-integral along contours in front of the crack tip.

Supplementary Note 3: Detailed strain data of individual load steps

The detailed strain data will be explained based on a total variational regularization of λ=0.01 and are depicted in Fig S3, with the respective rows denoting the individual load steps and the columns denoting the individual strain components ε_{yy}, ε_{zz}, ε_{yz} and ε_{xx}, respectively.

<u>Elastic loading (LS1, 22 mN)</u>

LS1 (22 mN, Fig. S3, first row) is still within the mostly elastic regime as evidenced from the load-displacement curves (Fig. S2a) and does not exhibit any pronounced 'butterfly type' shape, but only a very locally increased strain directly in front of the notch with an average opening strain in the 1 × 1 μm^2 crack tip vicinity of ε_{yy} = 0.019 (maximum ε_{yy} = 0.048). Considering the micro-tensile data[6], this would be right at the onset of yielding and suggests that already some small amount of local plastic deformation could have occurred at the crack tip. Further away, the transition from tension to compression regimes, resulting from the global bending load, is already evident with a neutral fiber (ε_{yy}=0) between 3.3-6.5 µm ahead of the crack tip.

<u>Transition from elastic to plastic loading (LS2, 45 mN)</u>

Upon loading to a peak load of 45 mN at LS2 (Fig. S3, second row) plastic deformation sets in, with an average tip opening strain of ε_{yy} = 0.15 (maximum ε_{yy} = 0.27) in a similar 1 × 1 μm^2 crack tip vicinity. Furthermore, two pronounced areas of locally increased strain are evident, extending from the crack tip under approximately 49° (towards base) and 29° (towards tip), respectively. The average strain within the highly strained tensile region (Fig. S3, region I) is ε_{yy} = 0.048 (maximum ε_{yy} = 0.058), while the average strain in the compressive bottom region (Fig. S3, region II) yields ε_{yy} = -0.049 (minimum ε_{yy} = -0.058). The equal but opposite magnitudes within these regions suggests a strong influence of the bending gradient very close to the crack tip (~3 µm distance), which inhibits the extension of the tensile strain field originating from the crack tip singularity. Considering these ε_{yy} data as a qualitative approximation for the shape of the plastic zone suggests that the external bending load on the specimen indeed leads to a major amount of mode I loading condition, since it would otherwise not display this 'butterfly type' shape, but rather a more confined region straight in front of the crack tip[21]. The current shape deviates from the idealized symmetric plastic zone as a result of the slight asymmetry of loading geometry, since the base of the cantilever remains considerably more rigid in comparison to the cantilever tip. The resulting constraint leads to an increase in plastic deformation on the base-facing side of the cantilever. Furthermore, the neutral fiber is pushed away from the crack tip to a distance between 5.6-7.4 µm as a result of the increased deformation around the crack tip.

<u>Plastically dominated crack extension (LS3, 43.6 mN)</u>

The last frame before unloading (SEM LS3, 43.6 mN, Fig. S2, third row) depicts a significant increase of the strain magnitude within the previously observed high deformation regions with, ε_{yy} = 0.332 (maximum ε_{yy} = 0.655) in the 1 × 1 μm^2 crack tip vicinity, as well as ε_{yy} = 0.102 (maximum ε_{yy} = 0.145) in the tensile region III (Fig. S3) and ε_{yy} = -0.058 (minimum ε_{yy} = -0.073) in the compressive region IV (Fig. S4). While the spatial extension of the plastically deformed region around the crack tip in *z*-direction is inhibited by the compression region in the lower part of the cantilever, the higher tensile strains at the crack tip as well as within the 'butterfly-type' region clearly indicate strong local plastic deformation as a result of the present crack tip. The comparatively lower strains within the compressive region are only marginally increased compared to LS2 (Fig S3), which suggests only minor plastic deformation therein. The strongly increased suppression by the bending strain gradient of local crack tip plasticity extending in *z*-direction, is also evidenced by the fact that the neutral fiber is approximately at the same position in front of the crack tip (5.0-8.5 µm) alike the previous peak load step (LS2, Fig. S3, second row).

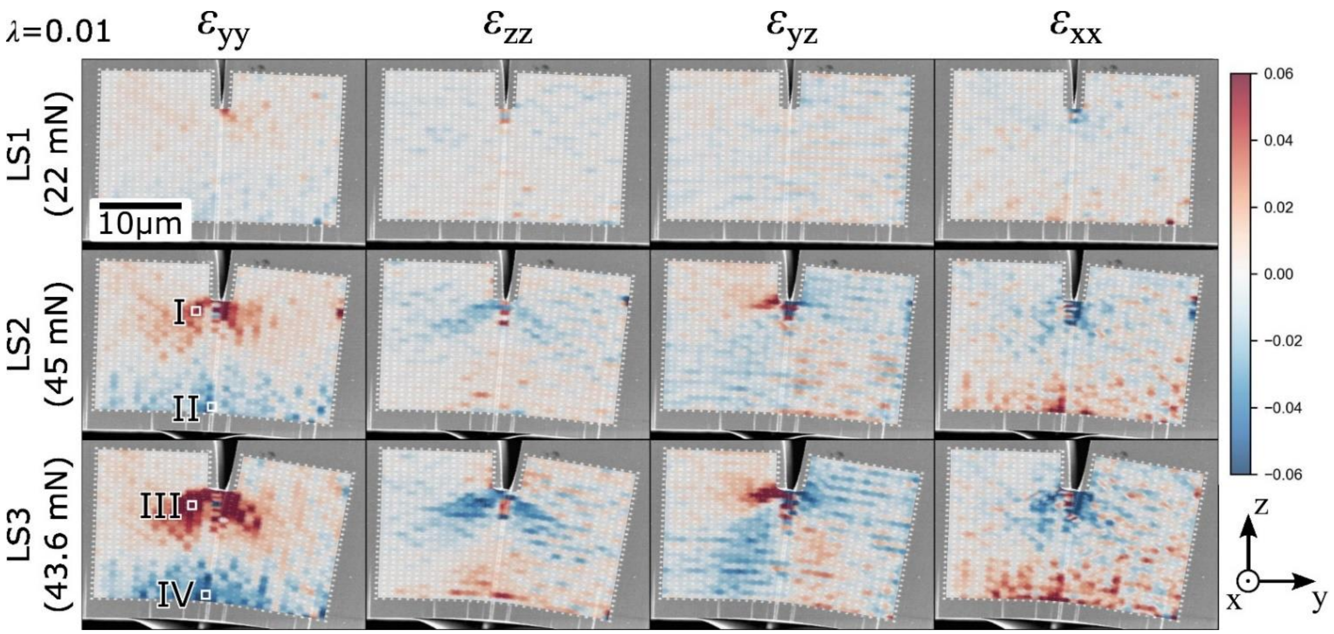

Figure S3. Strain data with a total variation regularization parameter of $\boldsymbol{\lambda}$**=0.01**

Strain data (normal strains ε_{yy}, ε_{zz}, ε_{xx} and shear strains ε_{yz}, respectively) for all loadsteps with a total variation regularization parameter λ=0.01. The micron bar is applicable for all subfigures and the regions I-IV denote positions which are described in more detail. This regularization parameter was chosen for all strain data used for the *J*-integral calculations in the manuscript.

Supplementary Note 4: Detailed CSnanoXRD microstructure and stress analyses

Qualitative CSnanoXRD phase analysis (not presented here) revealed a single phase fcc HEA, in agreement with Schuh *et al.*[1] and additionally indicated by the 2D diffractogram in Fig. S4a which exhibits exclusively Debye-Scherrer rings consistent with a fcc crystal structure. Within the X-ray phase analysis' resolution limits, no phase transformations were found throughout the *in situ* synchrotron experiment.

Qualitative texture analysis was carried out on the 111 and 200 DS rings of the HEA and is presented in Fig. S4b and S4c, respectively. The intensities are rather evenly distributed (Fig. S4a), with slight azimuthal maxima of the 111 DS at δ=±90 deg. These findings are in agreement with Skrotzki *et al.*[22,23], where a very weak brass-type shear texture was found.

A slight azimuthal dependency of the FWHM ranging from 0.362 to 0.271 deg between δ=0 and 90 deg size was revealed by the peak broadening analysis, respectively. This azimuthal variation of the FWHM corresponds to a coherently diffracting domains (SCDD) ranging from 13 to 17.5 nm showing a slight ellipticity of the grains as expected by a material processed by HPT.

Altogether, the microstructural data obtained by TEM[1–5], the weak crystallographic texture (Fig. S4a-c) and the rather equiaxed grain morphology (Fig. S4d) all suggest that it is possible to treat the material as quasi-isotropic.

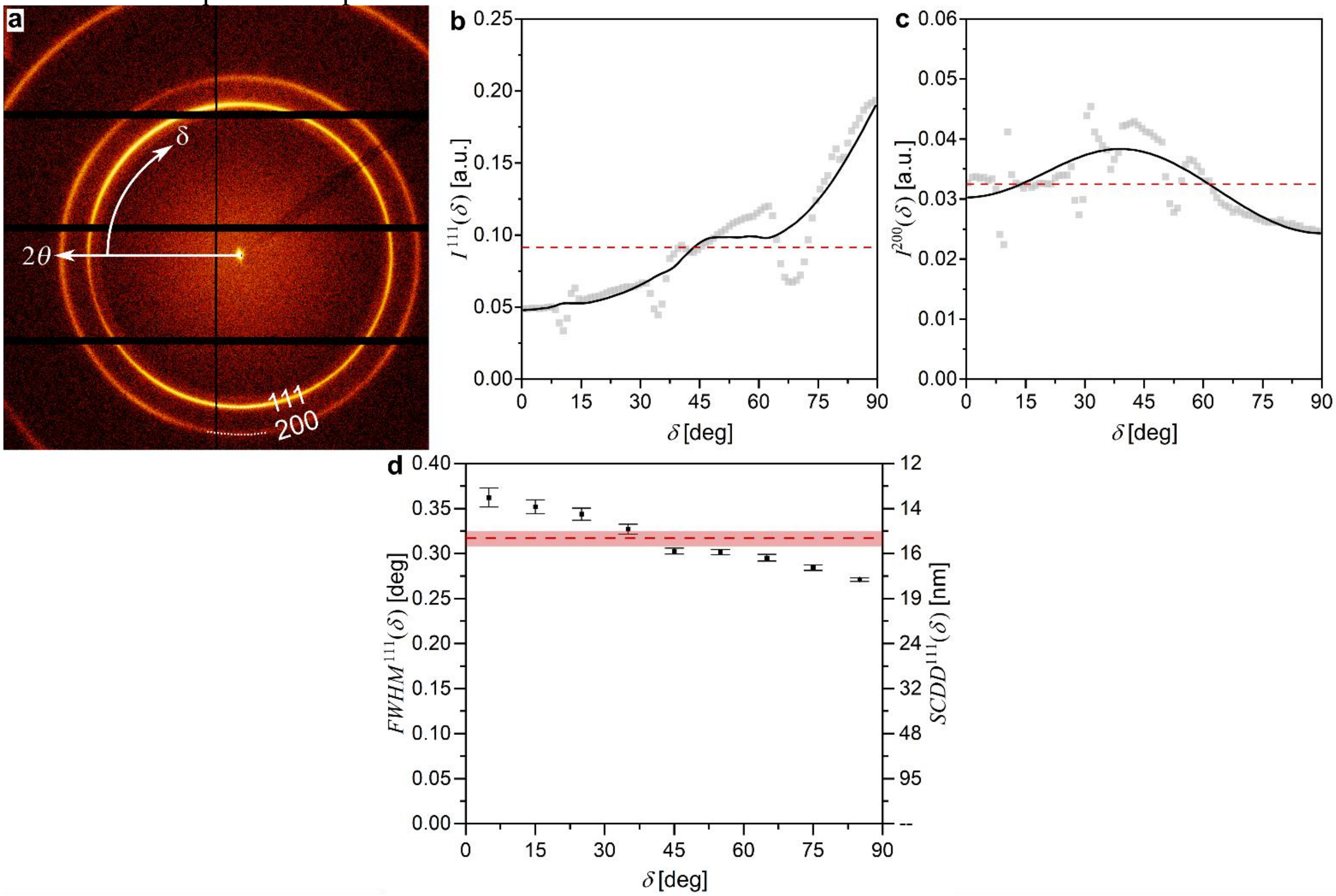

Figure S4. Experimentally obtained azimuthal intensity distributions.

In (a), a representative detector image displaying exclusively DS of the fcc HEA crystal structure are shown. Additionally, the azimuthal intensity distributions are presented for the 111 and 200 Debye-Scherrer rings in (b) and (c), respectively. The experimental data are shown in grey boxes, while the averaged profiles are shown in solid black lines. Additionally, the average intensity is presented as a dashed red line. Furthermore, in (d) the azimuthal dependency of the FWHM is presented. In (d), the

dashed red line represents the averaged FWHM presented in Fig. S4b, while the box indicates its standard deviation.

<u>Initial data before loading</u>

As detailed above, small angle X-ray scattering microscopy (SAXSM)[11,24–26] is sensitive to all sorts of electron density variations. Using the data gathered by SAXSM, before loading (LS0) a crack length of 10.2±0.2 µm was determined (Fig. S5a). Furthermore, an average FWHM of 0.317±0.008 deg was evaluated from the region of interest (*i.e.* LS0: 0mN, Fig. S5b), which is proportional to a size of coherently diffracting domains of 15.0±0.5 nm, a reasonable lower bound for the grain size of ~45 nm evaluated by transmission electron microscopy[1]. Because significant variations of the grain size are only expected during annealing[27,28] or cyclic loading of nanostructured materials prepared by high pressure torsion[29], further variations in the FWHM distributions can thus be correlated mostly to large concentrations and gradients of strains of 1st, 2nd and 3rd order as a consequence of the deformation within the X-ray gauge volume.

Before loading (LS0) minor stress concentrations averaging around -0.10±0.12, -0.01±0.10 and -0.00±0.05 GPa were evaluated for σ_{yy}, σ_{zz} and σ_{yz} components and are presented in Fig. S5c, S5d and S5e, respectively. Additionally, σ_{xx}, σ_{xy} and σ_{xz} distributions with minor magnitudes of ~0.0±0.39, -0.07±0.10 and -0.06±0.14 GPa, respectively, were evaluated and are shown in Fig. S5f, S5g and S5h, respectively. Additionally, the von-Mises stress $\sigma_{\text{von Mises}}$ (Fig. S5i) shows no significant stress concentrations.

The nonzero stress values can be related to minor uncertainties when determining the unstrained lattice parameter a_0, which are of a similar magnitude as the error value estimates retrieved from the overdetermined equation system (Eq. S9.1. These were calculated as ~±0.08 and ~±0.03 GPa for normal σ_{yy}, σ_{zz} and shear stress σ_{yz} components, respectively. The evaluated stresses include fitting error estimates of 0.24 GPa for σ_{xx} and 0.06 GPa for σ_{xy} and σ_{xz} (Eq. S9.2-S9.4). Please note that the σ_{xx} and σ_{zz} distributions are slightly broadened due to a possible a_0 gradient as discussed above. For clarity, all distributions of the evaluated stresses before loading are presented in Fig. S6. Furthermore, the uncertainties of the evaluated stress values are given by using Gaussian propagation of uncorrelated uncertainties[30] including the errors of the fitting process and the variability of the stresses before loading.

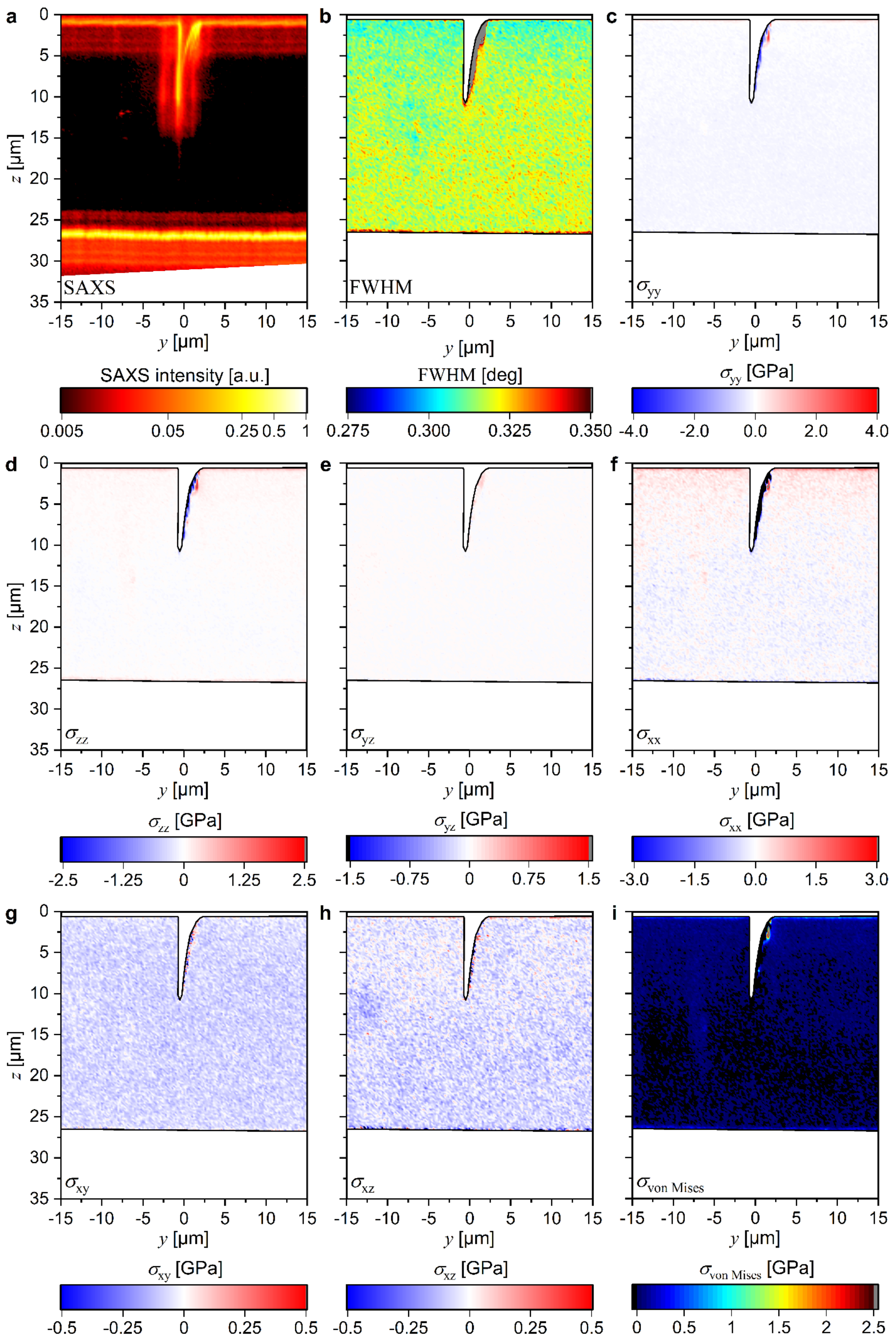

Figure S5. Experimentally obtained CSnanoXRD data for LS0.

In (a), the SAXS micrograph is presented, while in (b) the averaged FWHM is shown. In (c-h) all stress components before loading are presented, while in (i) the von Mises stress calculated from the former is shown.

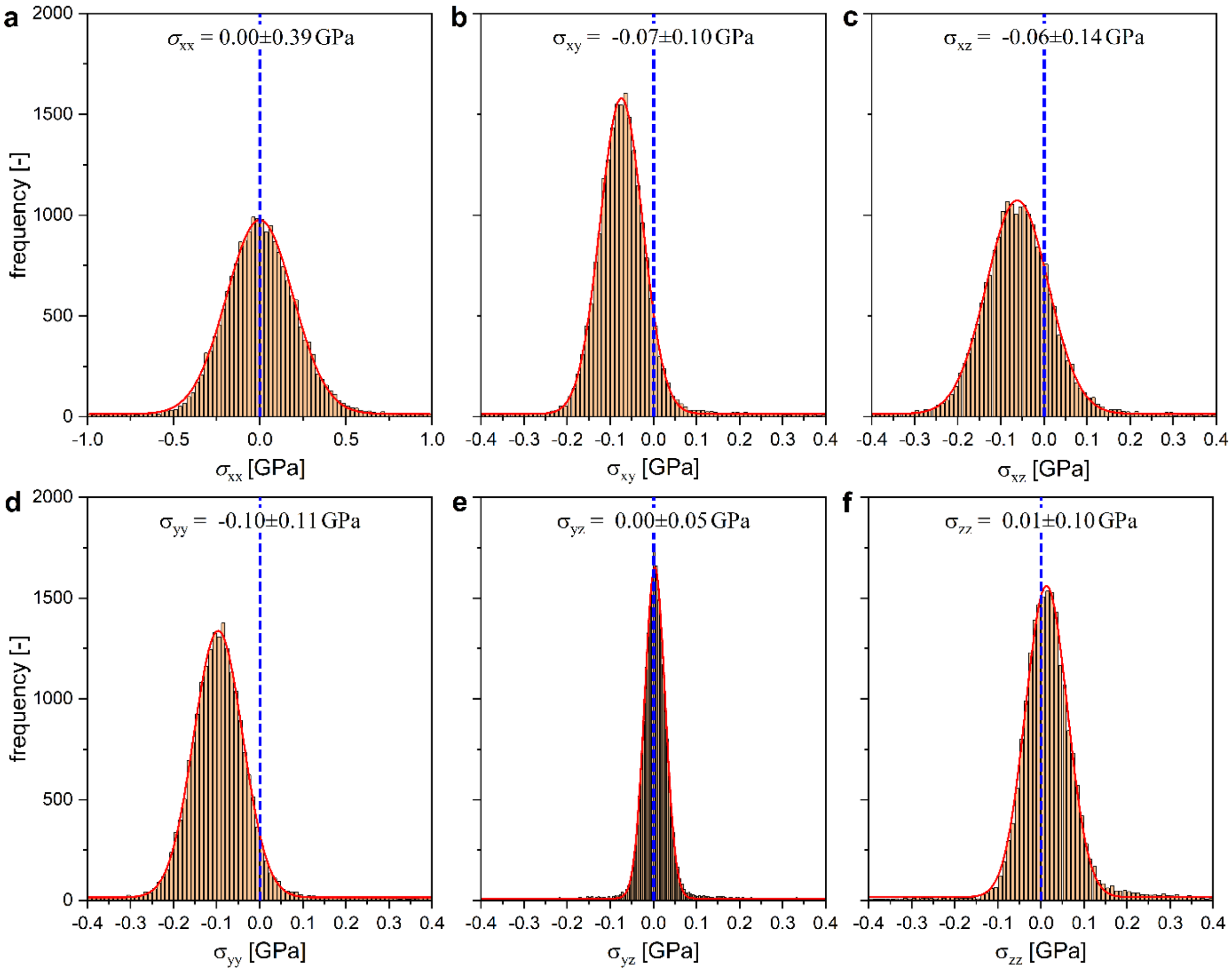

Figure S6. Frequency distributions of the stress tensor before loading

Frequency distributions of σ_{xx}, σ_{xy}, σ_{xz}, σ_{yy}, σ_{yz} and σ_{zz} before loading (LS0: 0 mN) are shown in (a), (b), (c), (d), (e) and (f), respectively. All stress components exhibit a Gaussian distribution with a correlation coefficient R^2>0.997. The nonzero σ_{xy} and σ_{xz} components reveal the limitations of the detector calibration (precise beam centre position). The average σ_{yy} of ~-0.10±0.11 GPa is an indication for remaining residual stress in the HPT-prepared material. Please note, that the unstrained lattice parameter a_0 was defined such that σ_{zz} equals ~0 at the surfaces.

Elastic loading (LS1, 22 mN)

After loading to 22 mN (LS1) the crack length increased to 11.0±0.5 µm, while no change in the lateral extension of the crack was recorded (Fig. S7a). Thus, the SAXSM data suggests a crack extension of 0.7 ± 0.28 µm, which is likely not conventional crack extension but opening of previously detached surfaces, which were held together by slight compressive stresses, due to the FIB processing and were only evident in the SAXS signal after effectively opening them upon elastic loading. Therefore, the SAXSM crack tip position at LS1 was considered as the effective initial crack tip for further evaluation.

At LS1, a gradual increase in the FWHM towards the notch was detected (Fig. S7b), which can be related mainly to the high gradients of stresses of 1st order within the X-ray gauge volumes when scanning close to the notch (Fig. S7c-S7i).

The σ_{yy} component far off the notch at y=-15 µm mostly exhibits a bending stress distribution ranging up to +0.59 GPa and -1.0 GPa (Fig. S8), slightly shifted from the ±0.84 GPa expected from geometrical considerations. Directly at the notch (y≈0 µm) σ_{yy} rises to ~3.69 GPa (Fig. S7c). Generally, the σ_{yy} stress distribution is governed by overlapping stress fields originating from the bending deformation and the stress introduced by the (nearly elastically) loaded notch. Similarly, σ_{zz} and σ_{xx} rise to ~2.47 GPa (Fig. S7d) and 2.49 GPa (Fig. S7f) directly at the notch tip, respectively, yielding a maximum stress difference of ~1.22 GPa between highest and lowest normal stresses. In addition to the global coordinates, the normal stresses are shown parallel and perpendicular to the crack growth direction in Fig. S9a and are thus called crack opening stress (*COS*), growth direction stress (*GDS*) and σ_{xx} (Fig. 1). In Fig. S9a, the stress evolution at the crack tip can be seen in detail, confirming the above stated normal stress maxima. Despite the comparably low rotation of the beam during deformation and the little difference between *COS* and *GDS* from σ_{yy} and σ_{zz} evaluated in the global *x*-*y*-*z* coordinate system, respectively, crack tip stresses are depicted more accurately in the local coordinate system.

Furthermore, the in-plane shear stress σ_{yz} (Fig S7e) is antisymmetric along the crack growth direction. It follows the elastic contributions introduced by the loaded crack tip. In detail, the shear stress is zero directly in front of the crack and at angles of ±60 deg with respect to the *z*-axis[31], while the minimum and maximum shear stresses are measured perpendicular to the crack tip, reaching ~-0.76 GPa and 0.84 GPa, respectively. The slight asymmetry in magnitude between the maximum and minimum shear stress of ~0.04 GPa is introduced via the cantilever bending geometry[11,32]. Additionally, the σ_{xy} component is only raised in a small zone in front of the crack tip approximately where the deviation from the $r^{-0.5 1/2}$-behaviour of the normal stresses is present, while there is no apparent variation of σ_{xz} during elastic loading (Fig. S7g and S7h, respectively).

The $\sigma_{\text{von Mises}}$ distribution is presented in Fig. S7i, respectively, and exhibits a typical butterfly-like shape. Furthermore, the maximum of $\sigma_{\text{von Mises}}$ is ~1.60 GPa, significantly below the yield stress of the nanocrystalline HEA of ~2 and 2.35 GPa evaluated by macro-[1] and microtensile[6] experiments, respectively, indicating that the criterion for bulk flow is not met in LS1.

In Fig. 4a, it is evident that the stress distributions are mostly governed by the elastic (*K*-) field represented by the $r^{-0.5}$-behaviour of the stress components, exhibiting similar magnitudes in a region between 0.5 and 1.5 µm in front of the crack tip. Only at distances below 200 nm and above 2.5 µm, the stresses deviate from the linear elastic behaviour, indicating the onset of the PZ in front of the notch and the bending stress gradient, respectively.

In order to determine the stress state in front of the crack tip (and to verify the reliability of the evaluated σ_{xx} values), the *PST* ratio introduced in Eq. 2 is shown for LS1 in Fig. 4d. Between the neutral fibre and the crack tip, *PST* ratio yields ~0.25 and is slightly increasing towards the notch. Additionally, the frequency distribution of the *PST* ratio was evaluated as 0.248±0.196 (Fig. 4g). Although, the distribution is rather broad given the high uncertainty of the evaluated σ_{xx} stress values (supporting information), the mean value is in good agreement to Poisson's ratios υ_{exp} of 0.253±0.017 and 0.25±0.1 as evaluated by uniaxial microtensile tests[6] and resonance peak measurements on bulk specimens of the

same material[33], respectively. Therefore, it is established that the cantilever is mainly under plane-strain conditions in front of the notch, according to elastic fracture mechanics theory[31].

Similar to the *PST* ratio, the stress triaxiality ratio T (Eq. 2) gives insight into the nature of the stress concentrations in front of the notch. Generally, a high tensile stress triaxiality favours crack growth by void formation and coalescence over plastic shear deformation, *i.e.* shear lip formation. [31,34]. T is presented in Fig. 4j, where far off the notch, T is $\sim \pm 1/3$ above and below the neutral fibre, respectively, corresponding to uniaxial bending (*i.e.* uniaxial tension and compression above and below the neutral fibre). Towards the crack tip T increases significantly (*cf*. Fig. 4j) and reaches values above 2 (note that $2/3$ and ∞ represent equibiaxial and equiaxial hydrostatic tension, respectively [31]).

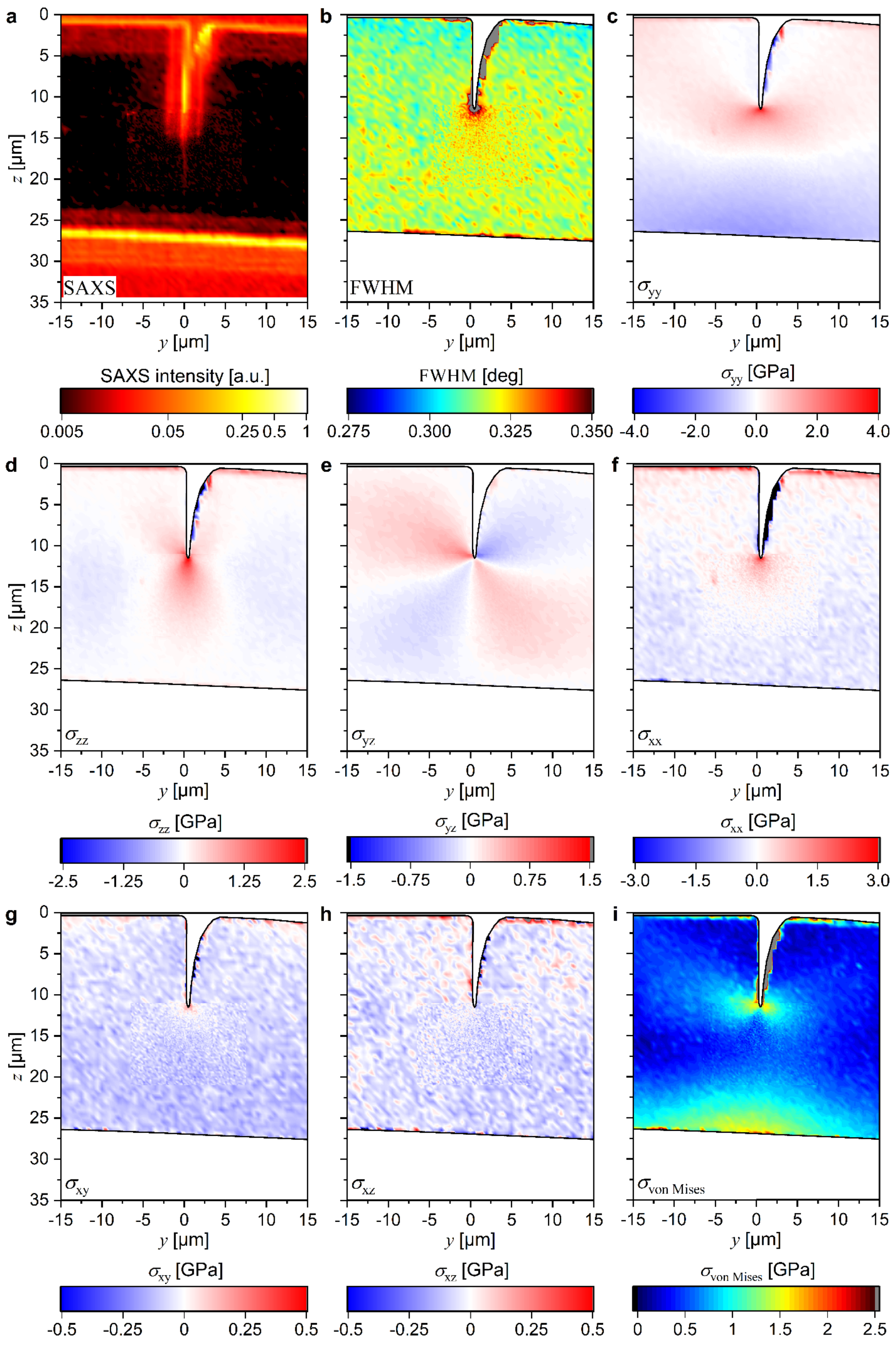

Figure S7. Experimentally obtained CSnanoXRD data for LS1.

In (a), the SAXS micrograph is presented, while in (b) the averaged FWHM are shown. In (c-h) all stress components before loading are presented, while in (i) the von Mises stress calculated from the former is shown.

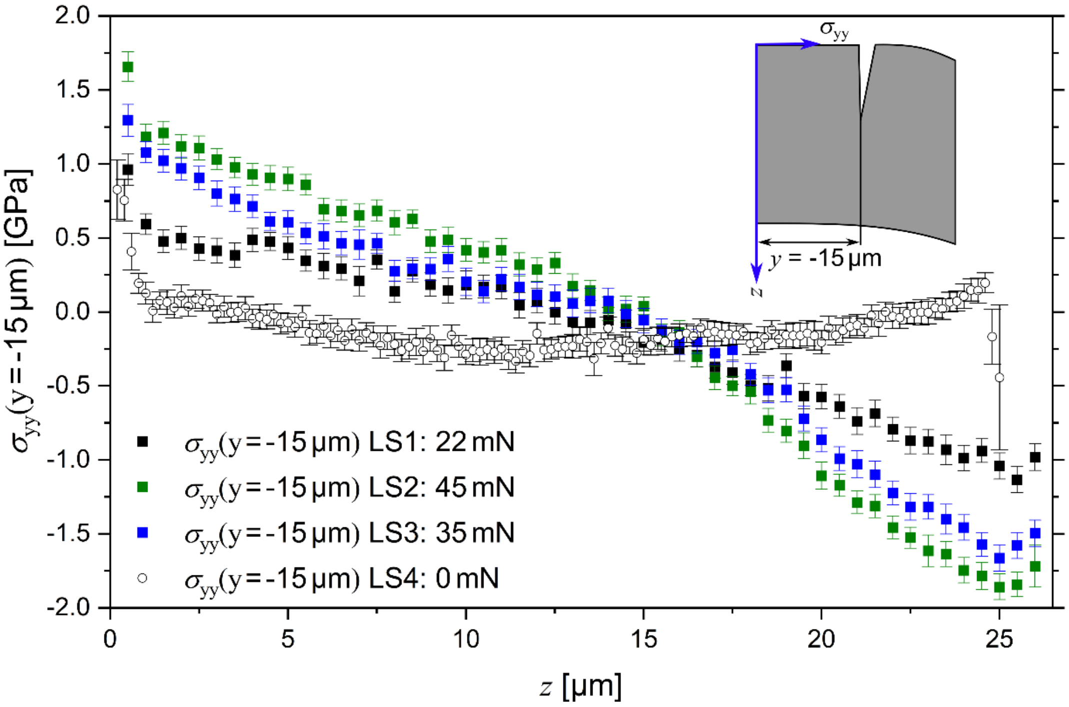

Figure S8. σ_{yy} stress component at *y*=-15 µm

σ_{yy} stress profiles at the edge of the investigated area (*y*=-15 µm) for LS1, LS2, LS3 and LS4. While at LS1 (black squares) the stress gradient is approximately linear, at LS2 and LS3, a bilinear stress gradient was evaluated, indicating the extension of the crack tip stress field throughout the whole measurement area. The error bars depict the standard deviation of the stress results.

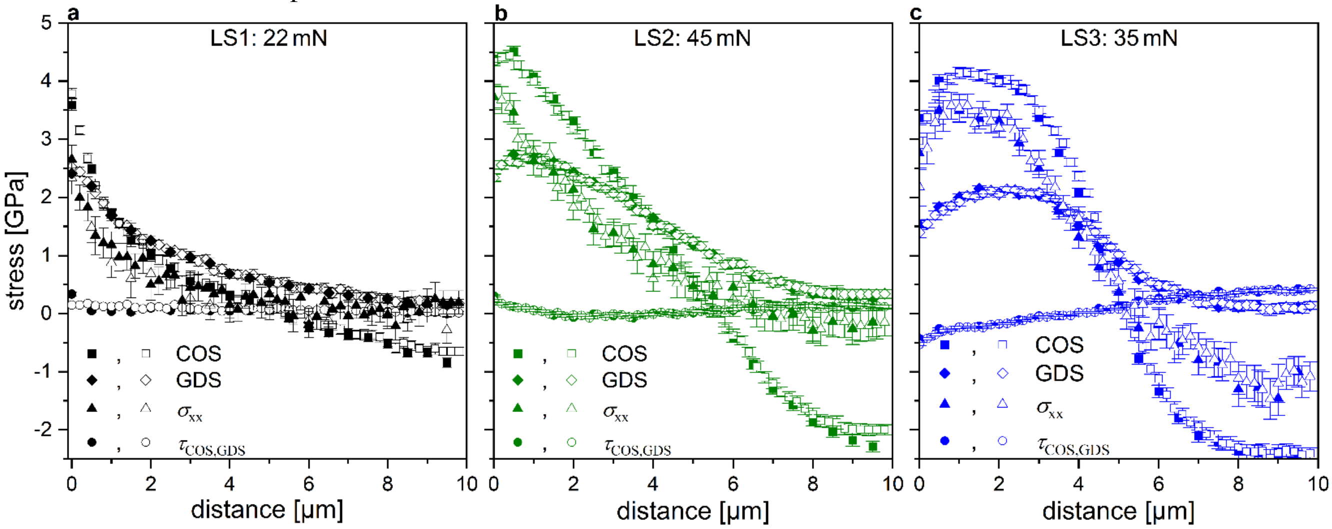

Figure S9. Crack tip stresses in linear coordinates.

Crack opening stress (COS), growth direction stress (GDS) and σ_{xx} aligned to the relative coordinate systems presented in Fig. 1c and the attributed shear stress $\tau_{COS,GDS}$ are presented for LS1, LS2 and LS3 in (a), (b) and (c), respectively. The shear stress is close to 0 for LS1 (a) and LS2 (b) indicating the crack-governed stress state, while at LS3 (c) a gradual increase of the shear stress is observed. Please note that the filled symbols represent the data recorded with 500 nm step size, while the empty symbols represent the data acquired with a step size of 200 nm. The error bars depict the standard deviation of the stress results.

Transition from elastic to plastic loading (LS2, 45 mN)

After loading to 45 mN (LS2) the crack length increased to 11.4±0.5 µm, with clearly detectable crack tip blunting, expressed by a rounded crack tip (Fig. S10a). Additionally, the FWHM increased in the region up to ~8 µm in front of the crack tip (Fig. S10b), which corresponds also to the region of high stress triaxiality (Fig. 4k) and is an indicator for defect accumulation (*e.g.* dislocations, stacking faults) as carriers of plastic deformation originating from the crack tip. Conversely, a decrease of the FWHM is evident in the highly compressive region at the lower part of the specimen. This may indicate a recombination/removal of mobile defects introduced through the HPT process[7,35,36].

After loading to 45 mN (LS2), the bending stress gradient of σ_{yy} exhibits magnitudes up to 1.21 GPa and -1.84 GPa far off the notch at *y*=-15 µm (Fig. S8), while at *y*=~0 µm in front of the crack tip and at the lower end of the cantilever σ_{yy} surpasses 4.35 GPa and ~-2.5 GPa (Figs. S9b, S10c), respectively. The different tensile and compressive bending stress magnitudes at *y*=-15 µm clearly reflect the larger extension of the stress fields introduced by the crack tip. The drastic stress increase is also reflected by the σ_{xx} and σ_{zz} components where the peak values in front of the crack tip reach levels of 3.73 and 2.34 GPa, respectively (Figs. S9b, S10d, S10f), with no apparent change of the overall shape of the stress distributions.

Similarly to the normal stresses, the antisymmetric σ_{yz} distributions intensified with further loading (Fig. S8e), reaching maximum values of ~-1.05 and 1.16 GPa, where the difference between minimum and maximum values of ~0.1 GPa may be again attributed to shear force[32]. Butterfly-shaped tensile σ_{xy} distributions are found at LS2 (Fig. S10g), while the slightly enhanced σ_{xz} distributions appear to be antisymmetric to the projected crack path with a comparably high intrinsic scattering (Fig. S10h). Here, the tensile σ_{xy} magnitudes (Fig. S10g) look similar compared to $\sigma_{\text{von Mises}}$ distributions (Fig. S10i), which suggests that σ_{xy} may be an indicator for the plastic zone.

$\sigma_{\text{von Mises}}$ at LS2 is presented in Fig. S10i hand exhibits a typical butterfly-like stress distribution. In front of the crack tip, maximum of $\sigma_{\text{von Mises}}$ was found to be at ~2.04 GPa, close to the yield stress of ~2 GPa[1] and 2.35 GPa[6] evaluated from nanocrystalline HEA. However, at LS2, the maximum $\sigma_{\text{von Mises}}(y,z)$ value of ~2.51 GPa, was evaluated from the lower half of the cantilever, where the stress state is close to uniaxial compression (Fig. S10i). The analysis of $\sigma_{\text{von Mises}}$ magnitudes thus indicates that the yield stress is reached both in front of the crack tip and also at the bottom of the cantilever.

At LS2, *COS*, *GDS* and σ_{xx} surpasses 4.35, 2.34 and 3.73 GPa in front of the crack tip respectively (Fig. 5b). Directly around the notch up to ~1 µm distance, the *COS* and the *GDS* show overall agreement with the elastic-plastic concept, *i.e.* $r^{-0.02}$. This suggests that the crack tip stress field closely follows the theoretical HRR description and is therefore governed mainly by the plastic deformation at this stage of the experiment. The linear-elastic (*K*-dominated) zone would be given by a stable $r^{-0.5}$-behaviour of *COS*, *GDS* and σ_{xx}, respectively. This trend, and therefore a *K*-dominated regime is not clearly discernible anymore, as the slope within the double-logarithmic graph shows a continuous change, rather than a constant discrete region (Fig. 4b). This suggests that the crack tip stress field closely follows the theoretical HRR description and is therefore governed mainly by the plastic deformation at this stage of the experiment.

Contrary to LS1, where the *PST* is ~0.25 and close to Poisson's ratio, in LS2 the *PST* increases from the neutral fibre towards the crack tip (Fig. 4e). The frequency distribution of the *PST* ratio given in Fig. 5h, shows that the mean value is raised to ~0.344 and maximum values are approaching 0.5 indicating ideal plastic deformation.

Furthermore, at LS2, far away from the notch *T* is around $\pm 1/3$ above and below the neutral fibre, respectively, while between the notch and the neutral fibre, in an almost circular zone of ~5 µm in diameter, T is larger than $1/\sqrt{3}$ indicating an increase towards higher hydrostatic tensile stress components, with the highest *T*-values along the projected crack path (Fig. 4k). However, up to 1 µm from the crack tip, *T* is smaller than 2, which is in good agreement with the range of the HRR field

identified in Fig. 4b, which acknowledges the plastic deformation and likely onset of void formation in front of the crack as evidenced in Fig. S2.

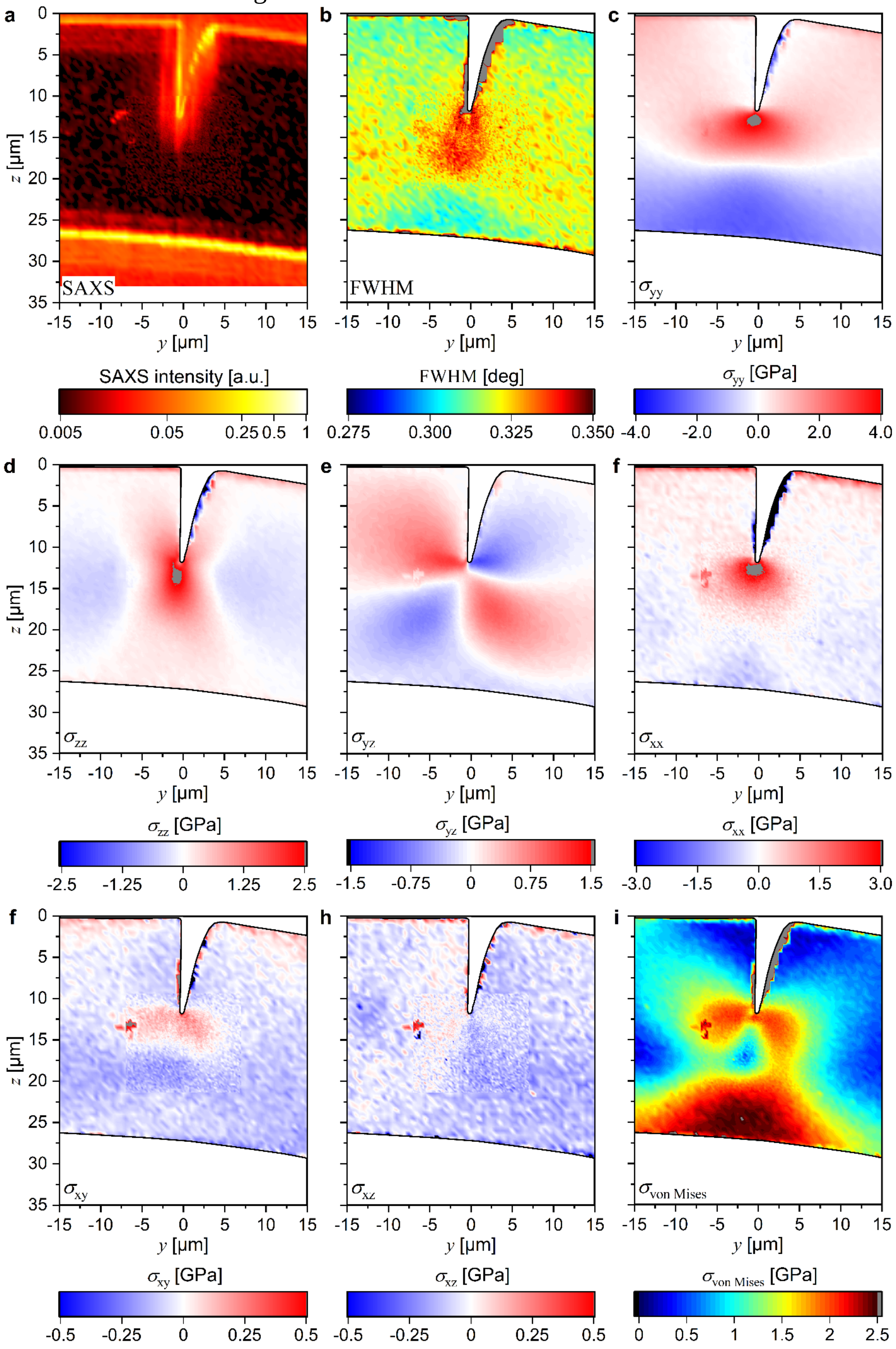

Figure S10. Experimentally obtained CSnanoXRD data for LS2.

In (a), the SAXS micrograph is presented, while in (b) the averaged FWHM is shown. In (c-h) all stress components before loading are presented, while in (i) the von Mises stress calculated from the former is shown.

Plastically dominated crack extension (LS3, 35 mN)

After advancing to the maximum deformation (LS3 at 35 mN load) significant crack growth to 14.0±0.5 µm could be extracted from the SAXSM micrograph (Fig. S11a), together with a pronounced sharpening of the crack tip. After loading to the maximum deformation (LS3: 35 mN), the FWHM is highly increased in a region at ~8 µm distance from the crack tip (Fig. S11b), which also corresponds well to a region of high stress triaxiality (Fig. 4l). Furthermore, in regions traversed by the crack, the FWHM evidently decreased below its initial value (Fig. S11b). This suggests local annihilation of mobile defects (*e.g.* dislocations) upon unloading[37] or even slight grain growth, in analogy to the microstructural grain growth observed in cyclic deformation of such severely deformed materials[29,38]. There the continuous accumulation of dislocations at grain boundaries leads to increased mobility and mechanical driving force for grain boundary migration. Given the change of stress magnitudes and triaxiality the case of a passing crack could be interpreted as a single localized load cyclic in close proximity to the crack path. Additionally, formation of a shear band is evident by a well-defined path of decreased and increased FWHM in the tensile and compressive stressed cantilever, respectively (indicated by arrows in Fig. S11b). This shear band counteracts the FWHM reduction observed at highest load (LS2: Fig. S10b) and suggests an increased dislocation activity in the lower (compressive) region of the specimen.

At LS3, the σ_{yy} and σ_{zz} magnitudes are significantly reduced compared to LS2 (Fig. S10), while σ_{xx} magnitudes increase further (Fig. S11). In detail, the bending stress gradient of σ_{yy} exhibits magnitudes up to +1.08 and -1.66 GPa at y=-15 µm (Fig. S8), clearly reflecting the large extension of the crack tip stress fields. At y=~0 µm significantly reduced σ_{yy} magnitudes of 3.38 GPa and ~-2.45 GPa were evaluated in front of the crack tip and at the lower end of the cantilever (Figs. S9c, 11c and 11d), respectively. The maximum *COS* and σ_{xx} reach 4.14 GPa and 3.53 GPa, respectively, at ~1 µm distance from the crack tip, while the maximum *GDS* of 2.14 GPa is measured at 2.2 µm distance from the crack tip (Fig. S7c). Due to the drastic reduction of the GDS from the crack tip over a distance of ~2 µm, the difference between *COS* and *GDS* is above 2 GPa. Since *COS* and *GDS* are also principal stresses, their difference in magnitude leads to high shear contributions and therefore favorably triggers plastic deformation instead of crack growth in front of the crack tip.

Furthermore, rhombic patterns of undulating increased and diminished σ_{yy}, σ_{zz} and σ_{yz} are observed in the compressive region of the cantilever (Fig. S11c,d,f), respectively. This can be attributed to multiple perpendicular shear bands forming to accommodate the deformation within the compressively stressed lower part of the cantilever. Here, the most prominent feature is a major shear band, which significantly reduces σ_{zz} close to zero stress on the right side of the specimen (Fig. S11d) and decreases σ_{yy} (Fig. S11c) along the same path where the FWHM distributions indicate shear band formation (Fig. S11b).

The σ_{yz} distributions remains antisymmetric along the projected crack path at LS3, with maximum values reaching ~-1.05 GPa and 1.24 GPa located perpendicular to the crack tip at y=-5.5 and 1.5 µm, respectively, and z~20 µm (Fig. S11e). Since the maximum geometrically induced shear stress is only ~0.08 GPa[32], the additional difference in shear stress may be induced by the shear band (Fig. S11).

In addition to the butterfly-shaped positive σ_{xy} distributions in front of the crack tip, the positive shear components extend all the way to the lower part of the cantilever at LS3 (Fig. S11g), further verifying the formation of a major shear band stretching from the crack tip to the cantilevers bottom surface (Fig. S11). The antisymmetric σ_{xz} distributions are stretched and shifted along the projected crack path (Fig. S11h) with slightly increased positive shear contributions towards the compressive cantilever side.

Finally, the $\sigma_{\mathrm{von\ Mises}}$ (Fig. S11i) stress distribution at LS3 is similar compared to LS2, however, the stress concentrations originating from the crack-tip and from bending start to overlap. They are linked by the shear band, which is also indicated by the stress concentrations, the FWHM distributions and the *PST* and *T* ratios (Figs. S11b, Fig. 4f,l). After loading to LS3, the maximum *COS* and σ_{xx} reach 4.14 GPa and 3.53 GPa, respectively, at ~1 µm distance from the crack tip, while the maximum *GDS* of 2.14 GPa is measured at 2.2 µm distance from the crack tip (*cf.* Fig. 4c). Due to the drastic reduction of the *GDS* between the crack tip and a distance of ~2 µm, the difference between *COS* and *GDS* is above 2 GPa. Since *COS* and *GDS* are also principal stresses, their difference in magnitude leads to high shear contributions and therefore favours plastic deformation over crack growth in front of the crack tip (*cf.* Fig. 4l). Generally, the plastic, elastic and bending-dominated zones are not distinctly discernible anymore (*cf.* Fig. 4c) since the transitions are continuous, rather than discrete.

At LS3, the *PST* increases further (Fig. 4f). Between the initial notch and the actual crack tip at LS3, the highest *PST* ratios are observed, which is attributed to remaining σ_{xx} magnitudes, while σ_{yy} and σ_{zz} are diminished as a consequence of the newly formed (crack) surfaces (Fig. S11). The frequency distribution of the *PST* ratio given in Fig. 4i, shows that the mean value is raised to ~0.569, which indicates complete plastic deformation[16] and suggests even some major pore formation due to the average exceeding the 0.5 threshold from constant incompressible volume arguments[39] as evident from post mortem images (Fig. 2b,c).

Additionally, at LS3, *T* is reduced in front of the crack tip (Fig. 4l) compared to LS2 (Fig. 4k). The highest *T* values are obtained outside the plastic zone (Fig. 4l) at ~2.9 µm distance from the crack tip. The decrease of *T* towards the crack tip is a further indication for plastic deformation instead of rupture/crack growth in front of the crack tip.

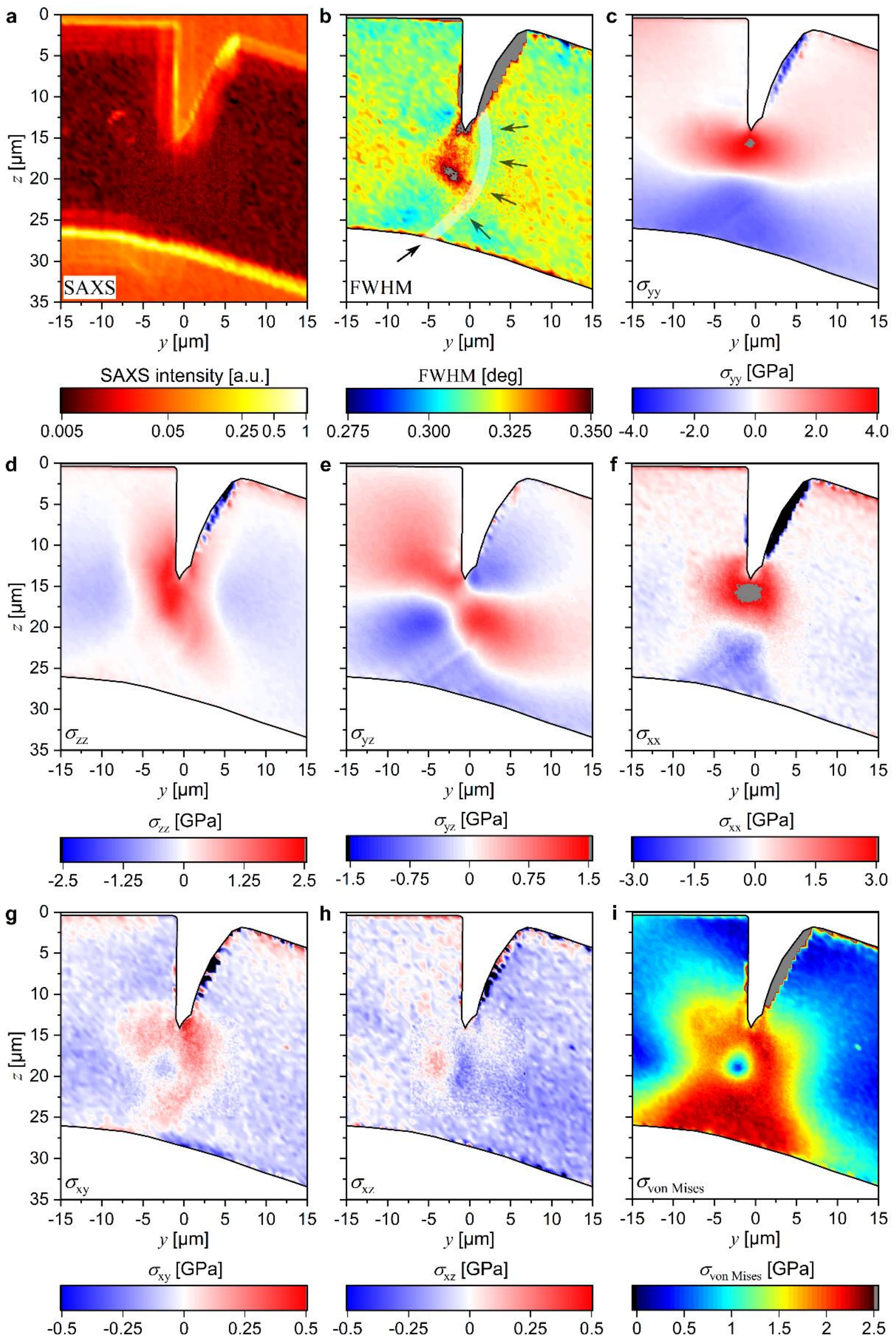

Figure S11. Experimentally obtained CSnanoXRD data for LS3.

In (a), the SAXS micrograph is presented, while in (b) the averaged FWHM is shown. In (c-h) all stress components before loading are presented, while in (i) the von Mises stress calculated from the former is shown.

Unloaded state after deformation

After unloading (LS4: 0mN), crack closure was detected to some extent, yielding a crack length of 12.8±0.2 μm in LS4 (Fig. S12a). In the highly deformed areas in front of the crack tip and at the bottom surface the FWHM decreased to values below the initial FWHM (Fig. S12b). This can again be attributed to two intertwined effects. Firstly, deformation will introduce additional mobile defects (*e.g.* dislocations, vacancies) which can annihilate with the large quantity of initial defects (mostly at grain boundaries)[40,41] introduced during the HPT process[42,43]. Secondly, this deformation and subsequent load reversal can be considered in analogy to a single cycle of plastic deformation, which is known to promote grain boundary migration[29,38], and might therefore lead to a slight increase in average grain size.

Furthermore, all stress distributions display residual stresses as a consequence of crack growth and plastic deformation (Figs. S12c-h). Crack closing during unloading leads to compressive σ_{yy} and σ_{zz} in front of the crack tip, while in the adjacent less-deformed region tensile stresses are preserved (Figs. S12c,d). Residual tensile stress σ_{yy} was evaluated at the bottom surface, while inside the material compressive stress originated as a result from the bending gradient (Fig. S12c). In case of σ_{zz} a crack-tip shielding residual compressive locus is evident, surrounded by an annular zone of tensile stress (Fig. S12d). Similarly, σ_{yy} the bottom of the cantilever remains in a tensile stressed state. The rather complicated σ_{yz} stress distribution presented in Fig. S10e shows mainly the rotation of the principal stress tensor[32].

Residual σ_{xx} stress distributions are presented in Fig S12f and display tensile residual stress around the crack tip as well as a zone of compressive stress extending ~5 μm halfway from the crack tip towards the adjacent surface. This compressively stressed zone is fully surrounded by a tensile stressed region. The σ_{xy} and σ_{xz} stress distributions after unloading presented in Figs. S12g and h, respectively, are rather unchanged in comparison to LS3 (Figs. S11g and h, respectively). Still, the tensile σ_{xy} distribution display the irreversible shear band and plastic zone around the crack tip very well (Fig. S12g).

Consequently, $\sigma_{\text{von Mises}}$ at LS4 after unloading (Fig. S12i) shows the stored elastic energy as a result of residual stress accumulation.

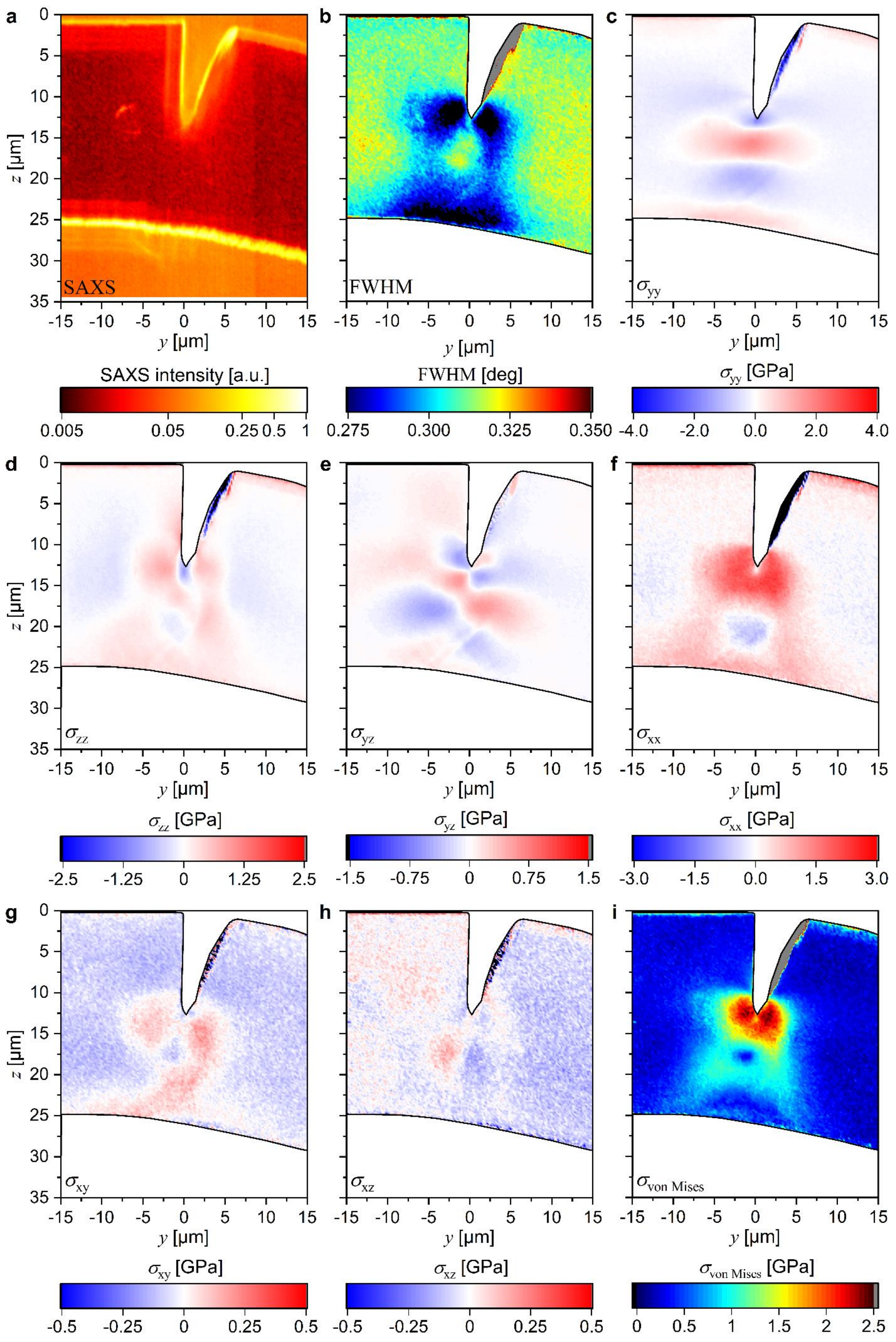

Figure S12. Experimentally obtained CSnanoXRD data for LS4.

In (a), the SAXS micrograph is presented, while in (b) the averaged FWHM is shown. In (c-h) all stress components before loading are presented, while in (i) the von Mises stress calculated from the former is shown.

Supplementary Note 5: Detailed FE analysis

In order to get further understanding of the stress accumulation in front of the crack tip, the experimental results were complemented by FE analysis. A detailed description of how the model was set up and how crack growth was accounted for is given in Suppl. Note 10. In following, analysis of the stresses is shown for LS1, LS2 and LS3.

Elastic loading (LS1, 20 mN)

The overall appearance of the modelled stress distribution at LS1 (Fig. S13) is nearly identical to experimental data presented in Fig. S7. In detail, the σ_{yy} component far off the notch at y=-15 µm mostly exhibits a bending stress distribution ranging between +0.6 and -0.99 GPa (Fig. S13a), in excellent agreement with the experimentally determined stress gradient (Fig. S8). Directly at the notch (y≈0 µm) σ_{yy} rises to ~5.25 GPa (Fig. S13a). Generally, the σ_{yy} stress distribution is governed by overlapping stress fields originating from the bending deformation and the stress introduced by the (nearly elastically) loaded notch. Similarly, σ_{zz} and σ_{xx} rise to ~3.41 GPa (Fig. S13b) and 3.12 GPa (Fig. S13d) directly at the notch tip, respectively, yielding a maximum stress difference of ~2.13 GPa between highest and lowest normal stresses. Furthermore, the in-plane shear stress σ_{yz} (Fig S13c) is antisymmetric along the crack growth direction. It follows the elastic contributions introduced by the loaded crack tip. In detail, the shear stress is zero directly in front of the crack and at angles of ±60 deg with respect to the z-axis[31], while the minimum and maximum shear stresses are measured perpendicular to the crack tip, reaching ~-1.39 GPa and 1.53 GPa, respectively. The slight asymmetry in magnitude between the maximum and minimum shear stress is introduced via the cantilever bending geometry[11,32]. Generally, the calculated stress magnitudes directly at the crack tip are higher compared to the experimentally observed stresses during elastic loading, which can be related (i) to the finite cross-section of the X-ray beam (Suppl. Note 9), which partly averages across the high stress gradients present at the crack tip, (ii) to the imperfect notch fabricated by FIB milling (Suppl. Note 6) and (iii) to local plasticity already present at the crack tip in the experiment.

The $\sigma_{\mathrm{von\ Mises}}$ distribution is presented in Fig. S13e, respectively, and exhibits a typical butterfly-like shape similar to the experimentally obtained $\sigma_{\mathrm{von\ Mises}}$ (Fig. S7i), while maximum $\sigma_{\mathrm{von\ Mises}}$ obtained by the FE-model is above 2.5 GPa in a very small region close to the crack tip.

Also for the calculated stress distributions, the stress triaxiality ratio T (Eq. 2) gives insight into the nature of the stress concentrations in front of the notch. T is presented in Fig. S13f, where far off the notch, T is $\sim \pm 1/3$ above and below the neutral fibre, respectively, corresponding to uniaxial bending, *i.e.* uniaxial tension and compression above and below the neutral fibre, respectively. Towards the crack tip T increases significantly (*cf.* Fig. 13f) and reaches values above 2 directly at the crack tip(note that $2/3$ and ∞ represent equibiaxial and equiaxial hydrostatic tension, respectively[31]).

The PST ratio (Eq. 2) obtained for the modelled stress distributions is shown for LS1 in Fig. S13g. Between the neutral fibre and the crack tip, PST ratio ranges between 018 and 0.4 and is slightly increasing towards the notch. Additionally, the frequency distribution of the PST ratio was evaluated at 0.18 (Fig. 4g). The mean value is slightly lower compared to the Poisson's ratios υ_{exp} of 0.253±0.017 and 0.25±0.1 as evaluated by uniaxial microtensile tests[6] and resonance peak measurements on bulk specimens of the same material[33], respectively. However, a slightly lower plane strain constraint was also evaluated by Narasimhan and Rosakis when applying 3D FE modelling at an elastically loaded crack tip.

Generally, at LS1, the direct comparison between the experimental data and the FE-model yields excellent agreement outside the immediate crack tip vicinity (Fig. S14a and d). The lower experimental stress magnitudes directly in front of the crack tip may be related to the finite notch radius obtained by the focused ion beam cantilever preparation and the finite X-ray gauge volume.

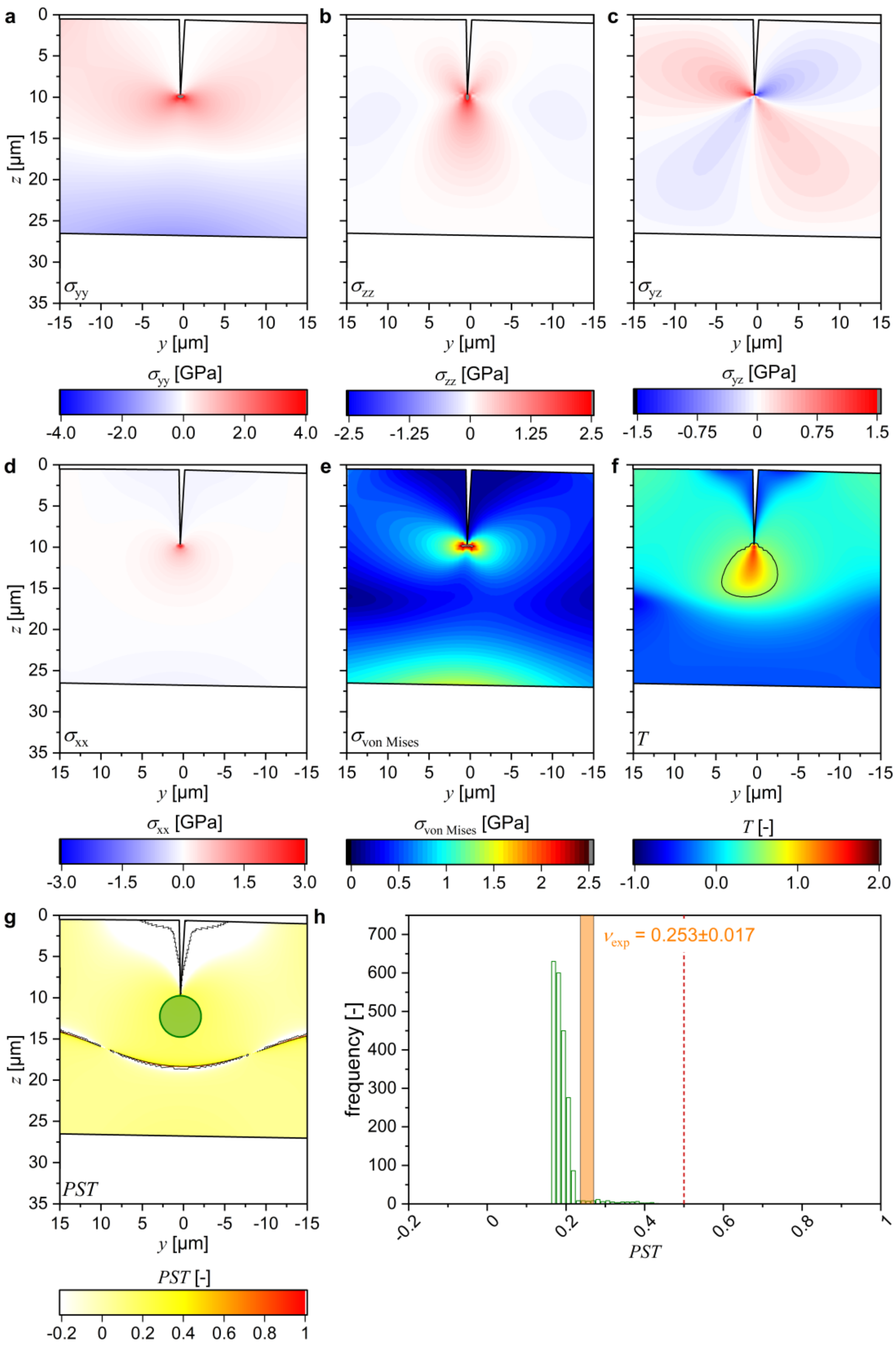

Figure S13. FE data obtained for LS1.

In (a), (b), (c) and (d) the individual stress components σ_{yy}, σ_{zz}, σ_{yz} and σ_{xx} are presented, respectively, while in (e) the von Mises stress calculated from the former is shown. In (f) the triaxiality ratio, being the relation between the hydrostatic portion of the stress tensor and the von-Mises stress representing the strain energy of distortion (Eq. 3), is shown. The black contour line in (f) corresponds to $T =$

$1/\sqrt{3} \approx 0.577$, representing a plane strain state. The *PST* value introduced in Eq. 2 to verify the evaluated crack tip stresses is presented in (g), where the black contour line marks PST = 0.5, which can be attributed to full plastic deformation. Finally, a circle with a diameter of 5 µm in front of the crack tip represents the area from which values were taken for the statistical analysis shown in (h). Additionally, Poisson's ratio υ_{exp} =0.253±0.01724 for this HEA is indicated in (h).

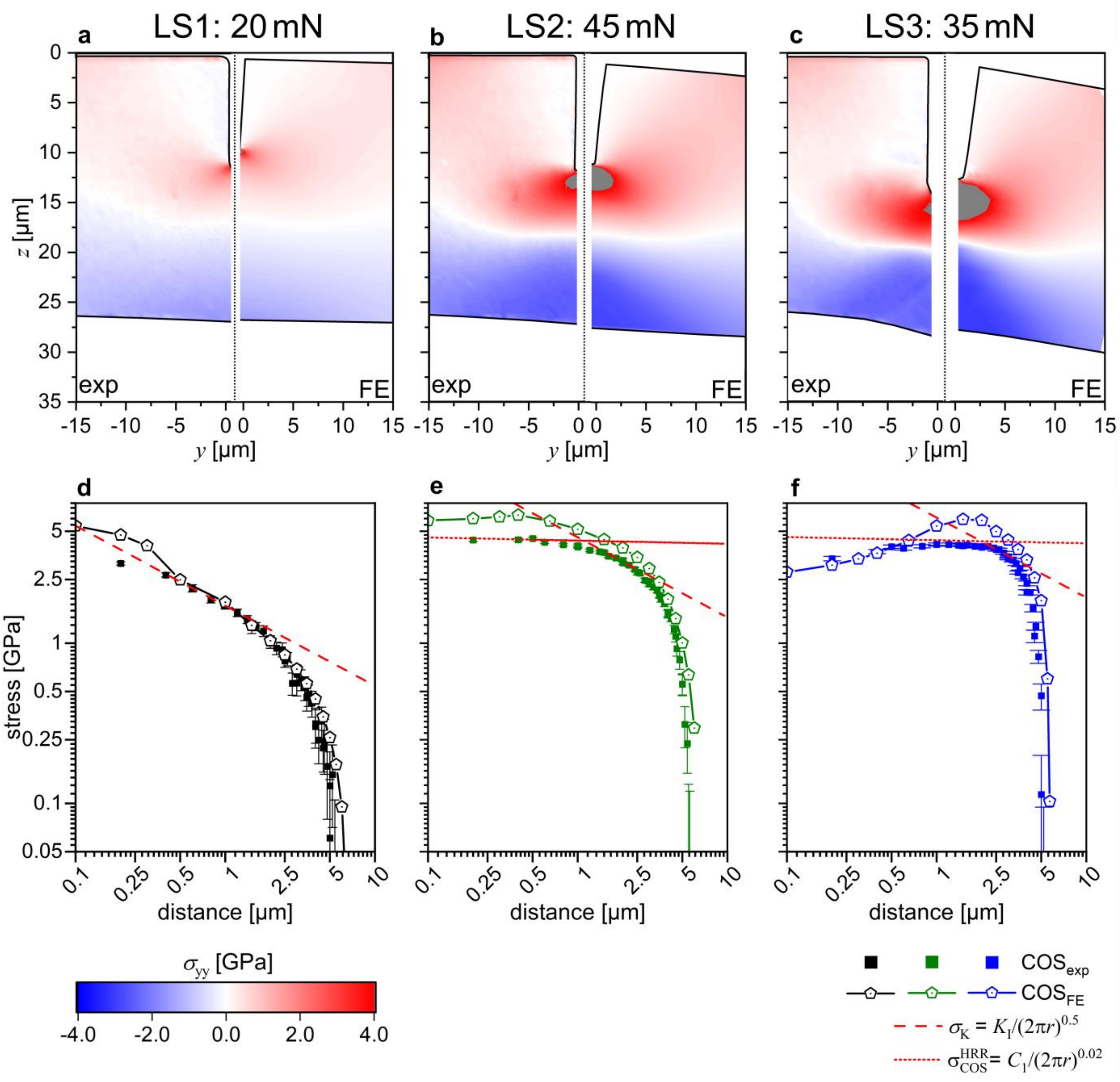

Figure S14. Comparison of experimental and modelled σ_{yy} distributions.

Experimental and modeled σ_{yy} distributions are shown on the left and right-hand side for LS1, LS2 and LS3 in (a), (b) and (c), respectively. The evaluated crack tip stresses are presented in (d), (e) and (f), respectively, where the full and open symbols indicate the experimental and modelled data.

Transition from elastic to plastic loading (LS2, 45 mN)

Also at LS2, the agreement between the model and experimental data is striking, as can be drawn from the comparison of Figs. S10 and S15. The bending stress gradient of σ_{yy} exhibits magnitudes up to 1.38 GPa and -2.41 GPa far off the notch at y=-15 µm (Fig. S15a), slightly larger than the experimentally obtained values (Fig. S8). The different tensile and compressive bending stress magnitudes at y=-15 µm clearly reflect the larger extension of the stress fields introduced by the crack tip.

At y=~0 µm in front of the crack tip and at the lower end of the cantilever σ_{yy} surpasses 6.80 GPa and ~-2.91 GPa (Figs. S15a), respectively, with the maximum tensile stress shifted 0.7 µm from the crack tip. While the tensile stress magnitude is clearly exaggerated by the model, the compressive stress at the bottom of the cantilever is only slightly enlarged compared to the -2.5 GPa found in the experiment. The drastic stress increase is also reflected by the σ_{xx} and σ_{zz} components where the peak values in front of the crack tip reach levels of 4.90 and 4.82 GPa, respectively (Figs. S15b, S15d), with no apparent change of the overall shape of the stress distributions. Note that the difference in normal stress is ~2 GPa, close to the yield stress of the nanocrystalline HEA.

Similarly to the normal stresses, the antisymmetric σ_{yz} distributions intensified with further loading (Fig. S14c), reaching maximum values of ~-1.52 and 1.52 GPa.

$\sigma_{\text{von Mises}}$ obtained from the modelled stress components at LS2 is presented in Fig. S15e and exhibits a typical butterfly-like stress distribution. In front of the crack tip, maximum of $\sigma_{\text{von Mises}}$ was found to be at ~2.75 GPa, clearly above to the experimentally determined yield stress of the nanocrystalline HEA ranging between ~2 GPa[1] and 2.35 GPa[6]. Contrary to the experiment at LS2, a $\sigma_{\text{von Mises}}(y, z)$ value of ~2.61 GPa lower then the stress in front of the crack tip was evaluated from the lower half of the cantilever, where the stress state is close to uniaxial compression (Fig. S15e). However, also in the model the analysis of $\sigma_{\text{von Mises}}$ magnitudes thus indicates that the yield stress is reached both in front of the crack tip and also at the bottom of the cantilever.

At LS2, far away from the notch T is around $\pm 1/3$ above and below the neutral fibre, respectively, while between the notch and the neutral fibre, in an almost circular zone of ~5 µm in diameter, T is larger than $1/\sqrt{3}$ indicating an increase towards higher hydrostatic tensile stress components, with the highest T-values along the projected crack path (Fig. S15f). Also here some difference from the experimental data was found, since the highest T values evaluated from the modeled data are found directly in front of the crack tip, while for the experimental data, the highest T was found at a distance of at least 1 µm from the crack tip (Fig. 4k).

Contrary to LS1, where the *PST* is close to Poisson's ratio, in LS2 the *PST* increases from the neutral fibre towards the crack tip (Fig. S15g) with values close to the crack tip reaching 0.5. The frequency distribution of the *PST* ratio given in Fig. S14h shows that besides the mean value at ~0.18 a significant portion of the values are higher and the maximum values are approaching 0.5 indicating ideal plastic deformation in front of the crack tip.

Also at LS2, the direct comparison between the experimental data and the FE-model yields very good agreement outside the immediate crack tip vicinity (Fig. S14b and e), with similar sizes of the plastic zone, while the deviations of the stress magnitudes increase compared to LS1.

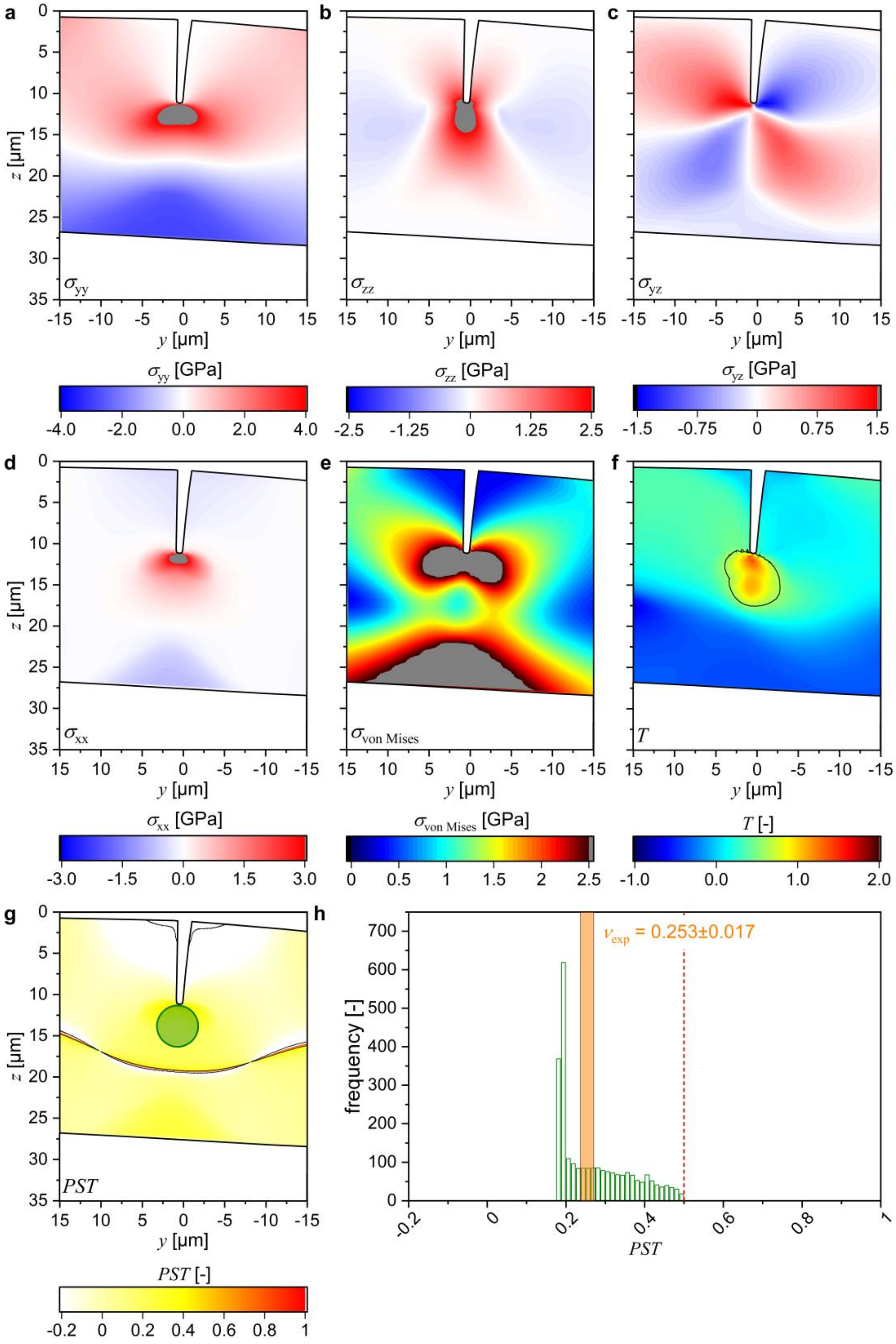

Figure S15. FE data obtained for LS2.

In (a), (b), (c) and (d) the individual stress components σ_{yy}, σ_{zz}, σ_{yz} and σ_{xx} are presented, respectively, while in (e) the von Mises stress calculated from the former is shown. In (f) the triaxiality ratio, being the relation between the hydrostatic portion of the stress tensor and the von-Mises stress representing the strain energy of distortion (Eq. 3), is shown. The black contour line in (f) corresponds to $T =$

$1/\sqrt{3} \approx 0.577$, representing a plane strain state. The *PST* value introduced in Eq. 2 to verify the evaluated crack tip stresses is presented in (g), where the black contour line marks PST = 0.5, which can be attributed to full plastic deformation. Finally, a circle with a diameter of 5 µm in front of the crack tip represents the area from which values were taken for the statistical analysis shown in (h). Additionally, Poisson's ratio υ_{exp} =0.253±0.01724 for this HEA is indicated in (h).

Plastically dominated crack extension (LS3, 43 mN)

At LS3, modeled stress magnitudes (Fig. S16) are slighty increased compared to LS2 (Fig. S15), in strong contrast to the experimentally obtained stress values (Fig. S11). In detail, the modeled bending stress gradient of σ_{yy} exhibits magnitudes up to +1.50 and -2.32 GPa at y=-15 µm (Fig. S16a), clearly larger than the experimentally obtained stress values (Fig. S8). At y=~0 µm, σ_{yy} magnitudes of 7.06 GPa and ~-3.00 GPa were evaluated in front of the crack tip and at the lower end of the cantilever (Figs. S16a), respectively. The maximum tensile stress obtained by the model was found at a distance of ~2.5 µm from the crack tip, which is comparable to distance of the maximum COS at LS3 (Fig. 4c), highlighting at least the qualitative agreement between model and experiment. The maximum σ_{zz} and σ_{xx} reach 4.67 GPa and 5.33 GPa, respectively, at ~2.5 µm distance from the crack tip (Fig. S15b and d).

The σ_{yz} distributions remains antisymmetric along the projected crack path at LS3, with maximum values reaching ~-1.51 GPa and 1.66 GPa located perpendicular to the crack tip, respectively. Finally, the $\sigma_{\text{von Mises}}$ (Fig. S15f) stress distribution at LS3 is similar compared to LS2, however, the stress concentrations originating from the crack-tip and from bending overlap completely.

Additionally, at LS3, *T* obtained from the modeled data is reduced in front of the crack tip (Fig. S16f) compared to LS2 (Fig. S15f). The highest *T* values are obtained halfway between the crack tip and the neutral fibre.

At LS3, the *PST* obtained from the modeled stress distributions increases further (Fig. S16h). Additionally, a channel of raised PST reaches from the crack tip to the bottom of the cantilever, similar to the experimental data presented in Fig. 4f. The frequency distribution of the *PST* ratio given in Fig. 4i, shows that the mean value is raised to ~0.50, which indicates complete plastic deformation[16].

At LS3, in the direct comparison, the FE-model yields further increase of the σ_{yy} stress magnitudes as can be drawn from Fig. 5c. The differences are for the first time clearly not only restricted to the immediate crack tip area (Fig. S14c and 14f). While the crack tip can be modeled properly, due to the apparent decrease of triaxiality (Fig. S16f), at higher distances, the stresses are significantly overestimated. Furthermore, the effect of crack growth is highlighted in the experimental data (in contrast to the model), a plastic wake is indicated by the slightly compressively stressed zone behind the crack tip, which is absent in the modeled σ_{yy} distributions (Fig. S14c).

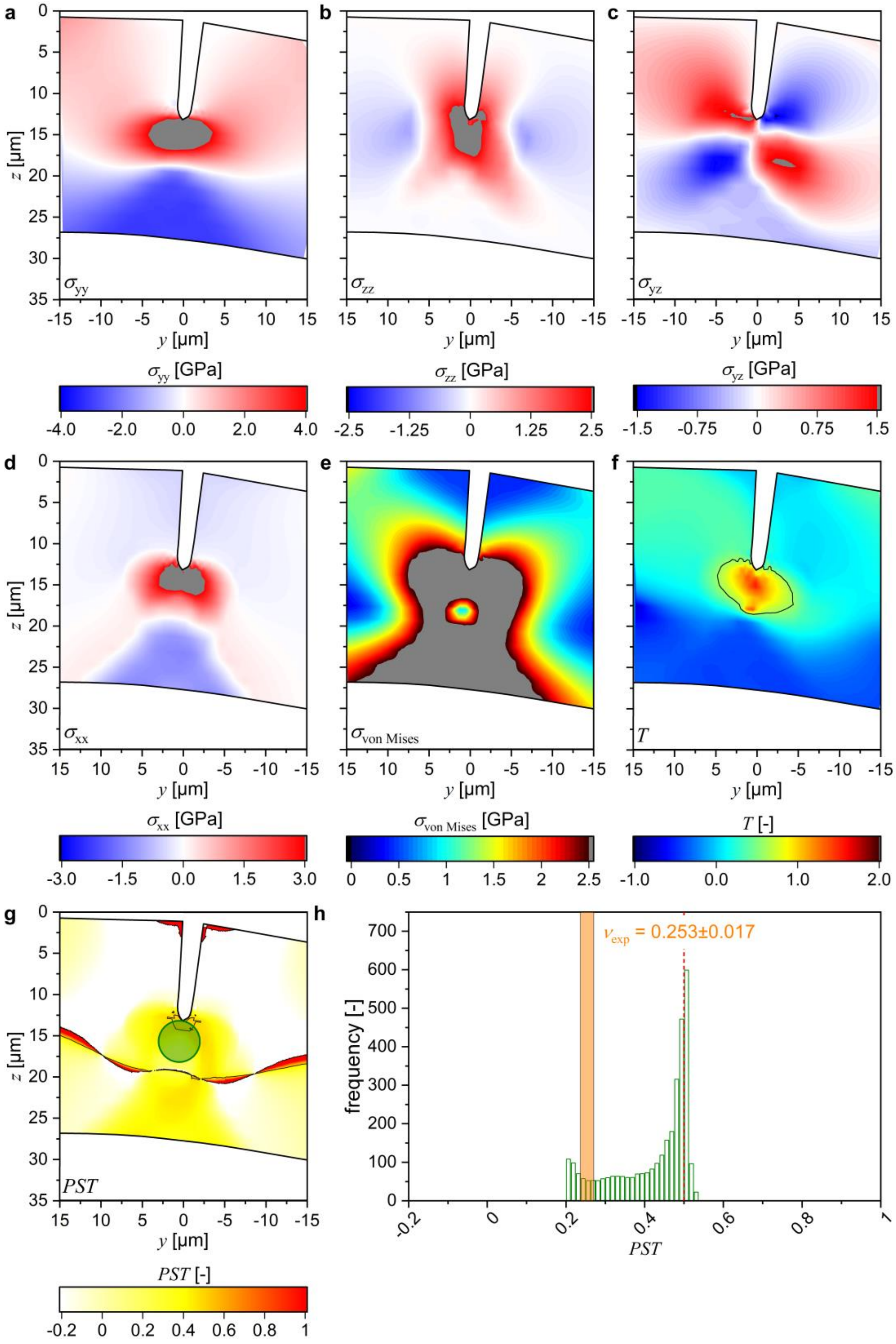

Figure S16. FE data obtained for LS3.

In (a), (b), (c) and (d) the individual stress components σ_{yy}, σ_{zz}, σ_{yz} and σ_{xx} are presented, respectively, while in (e) the von Mises stress calculated from the former is shown. In (f) the triaxiality ratio, being the relation between the hydrostatic portion of the stress tensor and the von-Mises stress representing the strain energy of distortion (Eq. 3), is shown. The black contour line in (f) corresponds to $T =$

$1/\sqrt{3} \approx 0.577$, representing a plane strain state. The PST value introduced in Eq. 2 to verify the evaluated crack tip stresses is presented in (g), where the black contour line marks PST = 0.5, which can be attributed to full plastic deformation. Finally, a circle with a diameter of 5 µm in front of the crack tip represents the area from which values were taken for the statistical analysis shown in (h). Additionally, Poisson's ratio υ_{exp} =0.253±0.01724 for this HEA is indicated in (h).

Supplementary Note 6: Details about the cantilever fabrication

An Auriga laser system (Carl Zeiss AG, Oberkochen, Germany) combining a FIB and a field emission SEM was used to fabricate the freestanding cantilevers. This system further includes an Origami 10 XP femtosecond laser (Onefive GmbH, Regensdorf, Switzerland) with a pulse duration of 500 fs, a wavelength of 515 nm and a minimal focal diameter at the sample surface of approximately 20 µm for the sample processing. Details on this specific upgrade of the system can be found elsewhere[44]. The cantilevers were prepared by consecutive steps of femto-second laser ablation and focused ion beam milling. First the rough shape of the cantilever was obtained by femto-second laser ablation. Afterwards, coarse polishing of the cantilever was achieved by FIB milling with 20 nA milling current for defining the geometry. After the coarse polishing (*cf.* Methods), the top surface at the beams' support was polished in a 45 µm wide area with 1 nA polishing current. In the centre of this area, a ~9 µm deep notch was introduced by employing a current of 500 pA in following steps: Overlaying rectangles of the cantilevers thickness and decreasing widths of 2.75, 2.25, 1.75, 1.25 and 0.75 µm were milled in consecutive steps and finally, a line element was milled in the centre of the notch. Last, the remaining surfaces of the area of interest were milled with a polishing current of 1 nA. resulting in a final geometry of the cantilevers of $W \times B$ =26.2-26.5 µm×28-29 µm, while the bending length L was chosen in such way, that a similar nominal stress intensity is applied at the notch tip for both cantilevers. The exact geometry of the cantilevers is presented in Fig. S17 and summarized in Table S1. The point patterns for feature tracking in SEM were milled with a current of 50 pA on the polished cantilevers surface by using a dotted bitmap mask in conjunction with the built in feature-milling software of the FIB[6].

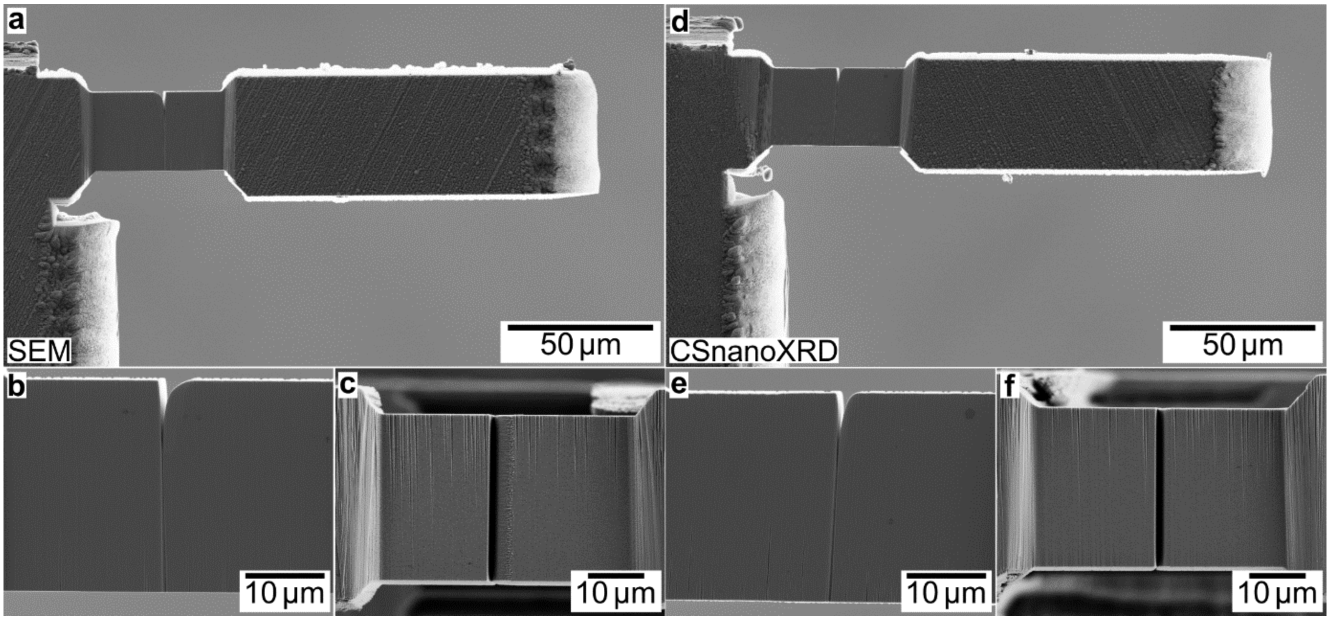

Figure S17. SEM images of FIB-prepared cantilevers
SEM images of FIB-prepared cantilevers deformed *in situ* in the SEM (a-c) and during the synchrotron CSnanoXRD experiment (d-f), respectively. The actual dimensions are presented in Table S1.

Table S1. Geometries of the cantilevers deformed *in situ* in the SEM and during the synchrotron CSnanoXRD experiment.

Here, L, B and W are the bending length, thickness and width of the cantilevers, respectively, while a is the initial crack length. Please note that the bending length L was adjusted such that a similar stress intensity was applied at the notches of both cantilevers tested in SEM and at the synchrotron, respectively.

	B [µm]	L [µm]	W [µm]	a [µm]
Cantilever 1 – SEM	27.9±0.2	123.8±0.6	26.2±0.1	9.2±0.1
Cantilever 2 – CSnanoXRD	29.0±0.2	129.0±0.6	26.5±0.1	9.2±0.1

Supplementary Note 7: Evaluation of the iterative *J*-integral (J_{iter})

The iterative *J*-integral data was evaluated at each unloading position *n* in analogy to macroscopic standards (ASTM1820) as [45]:

$$J_n = J_n^{el} + J_n^{pl} \tag{S7.1}$$

with the elastic and plastic parts:

$$J_n^{el} = \frac{K_{q,n}^2(1-\nu^2)}{E} \tag{S7.2}$$

$$J_n^{pl} = \left(J_{n-1}^{pl} + \frac{\eta}{W-a_n}\frac{A_n^{pl}-A_{n-1}^{pl}}{B}\right)\left(1-\gamma\,\frac{a_n-a_{n-1}}{W-a_n}\right) \tag{S7.3}$$

where K_q is the conditional linear elastic stress intensity, *E*=205 GPa is the Young's modulus, ν=0.253 is Poisson's ration[6], η=1.9, γ=0.9 are prefactors as per ASTM1820, *a*, *B* and *W* are geometric parameters as depicted in Fig.1 and A^{pl} is the plastic area under the load-displacement curve up to position *n*. The stress intensity and plastic area were calculated as:

$$K_{\mathrm{geom},n=0} = K_{q,n} = \frac{6\cdot P_n\cdot L}{W^2\cdot B}\sqrt{\pi\cdot a_n}\cdot Y\left(\frac{a_n}{W}\right), \tag{S7.4}$$

$$A_n^{pl} = A_n - A_n^{el} = \int_0^{u_n} P\,du - \frac{P_n^2}{2k_n} \tag{S7.5}$$

where *P* is the applied load, L, W, B and a are the geometry of the cantilever (Fig.1), *u* is the load line displacement, *k* is the cantilever stiffness measured from unloading segments and $Y\left(\frac{a_0}{W}\right)$ is a dimensionless geometry parameter taken from[46] as:

$$Y\left(\frac{a_0}{W}\right) = \sqrt{\frac{2W}{\pi a}\tan\left(\frac{\pi a_0}{2W}\right)}\,\frac{0.923+0.199\left(1-\sin\left(\frac{\pi a_0}{2W}\right)\right)^4}{\cos\left(\frac{\pi a_0}{2W}\right)}. \tag{S7.6}$$

The crack length for each individual unloading segment was determined based on the stiffness values *k* (linear slope in the *P*-*u* curve) from the numerically integrated equality[47]:

$$\int_0^{a_n}\frac{a}{W}\,Y\left(\frac{a}{W}\right)\,da = \frac{(k_0/k-1)}{18\pi(1-\nu^2)}\,\frac{(L+d)^3}{L^2} \tag{S7.7}$$

where k_0 is the stiffness of the virtually unnotched cantilever, back-calculated from the first elastic unloading slope as described in[48] and *d* = 25µm is the length from the crack tip to the base.

Finally, the iterative *J*-integral J_{iter} can be transformed into a *K*-value using the following equation

$$J_{\mathrm{iter}} = \frac{1-\nu^2}{E}K_{\mathrm{iter}}^2, \tag{S7.8}$$

Additionally, for LS1, the purely linear elastic stress intensity factor K_{geom} was evaluated using the geometry of the cantilevers, the initial crack length a_0 and the applied load using equation (S7.4).

Furthermore, the radius of the plastic zone in plane strain condition can be calculated from the individual *K*-values as follows [31]:

$$r_p = \frac{1}{6\pi}(K/\sigma_y)^2, \tag{S7.9}$$

where σ_y is the yield strength of the nanocrystalline HEA determined as 2355±66 MPa[6].

Supplementary Note 8: SEM - digital image correlation (SEM-DIC) strain analysis

The strain evaluation is based on a mesh of quadrilateral elements, analogous to FEM based approaches[6]. These previous experiments were conducted on tensile specimens, exhibiting a uniaxial loading and a Gaussian smoothing algorithm was found to yield good results with regards to reduction of stochasticity. However, using the determined parameters for the smoothing algorithm in the present experiments leads to artifacts at the free surfaces as well as removal of detail at the crack tip, due to 'blurring' of the strain field (Fig. S18 a,b). To reduce the strong stochasticity, resulting from slight sub-pixel deviations during feature tracking another non-linear smoothing scheme needs to be applied.

To adapt for more complex strain fields a smoothing algorithm based on total variational (TV) regularization[49] was employed for each displacement component: horizontal u(y,z) and vertical v(y,z), respectively. For simplicity the algorithm will be described for one component only, while the second one is treated analogous. Two quantities are calculated during each step of the iteration: the mean-squared error between the original and unsmoothed displacements $\boldsymbol{E}_{new}$, as:

$$\boldsymbol{E}_{new} = \frac{1}{n}\sum_{i=1}^{n}\left(u_{new}(y_i, z_i) - u(y_i, z_i)\right)^2, \tag{S8.1}$$

where i denotes and individual point, n is the total number of points. The second quantity is the variation $\boldsymbol{V}_{\mathbf{new}}$ of each displacement component with respect to the displacements in the direct vicinity (eight points around), as:

$$\boldsymbol{V}_{new} = \frac{1}{n}\sum_{i=1}^{n}\sum_{j=1}^{8}\left|u(y_i, z_i) - u(y_j, z_j)\right|, \tag{S8.2}$$

where j denotes the surrounding points for each point i. The goal is then to minimize the functional:

$$\min(\boldsymbol{E} + \lambda\boldsymbol{V}), \tag{S8.3}$$

where λ is a regularization weight. This essentially means finding a balance between the individual slight errors in displacement variation ($\boldsymbol{V}$), while still remaining as close as reasonable to the initially measured displacements ($\boldsymbol{E}$). The minimization is conducted using a gradient descend approach in n-dimensional space (922-dimensions in the present case). To ensure stability no gradient scaling, but constant steps of 0.001 px are used for each individual iteration. While the use of more sophisticated algorithms, such as a split-Bregmann approach[50] could be used to accelerate the minimization, it does not seem necessary, given the already short times of 10-20 s for the present work using only 3 cores of a 2.7 GHz processor.

In comparison to the previous Gaussian smoothing algorithm, where the maximum change in point position was as large as 1.3 px[6], the TV approach only changed the points marginally with a maximum position change of 0.65 px. Using the same data for a comparative sanity check as in the previous work on uniaxial tension experiments[6], where a Gaussian smoothing size of 30 px lead to a good agreement of the tension modulus (E = 205 GPa), a regularization parameter of λ = 0.01 lead again to an acceptable modulus value of E = 221 GPa, while a regularization parameter of λ = 0.02, overestimated the modulus already significantly at E = 244 GPa. Furthermore, it is evident that such high regularization parameters already obscure or completely remove detail in the strain maps as evident in comparison between Figs. S19 (raw data), S3 (λ = 0.01) and S20 (λ = 0.02).

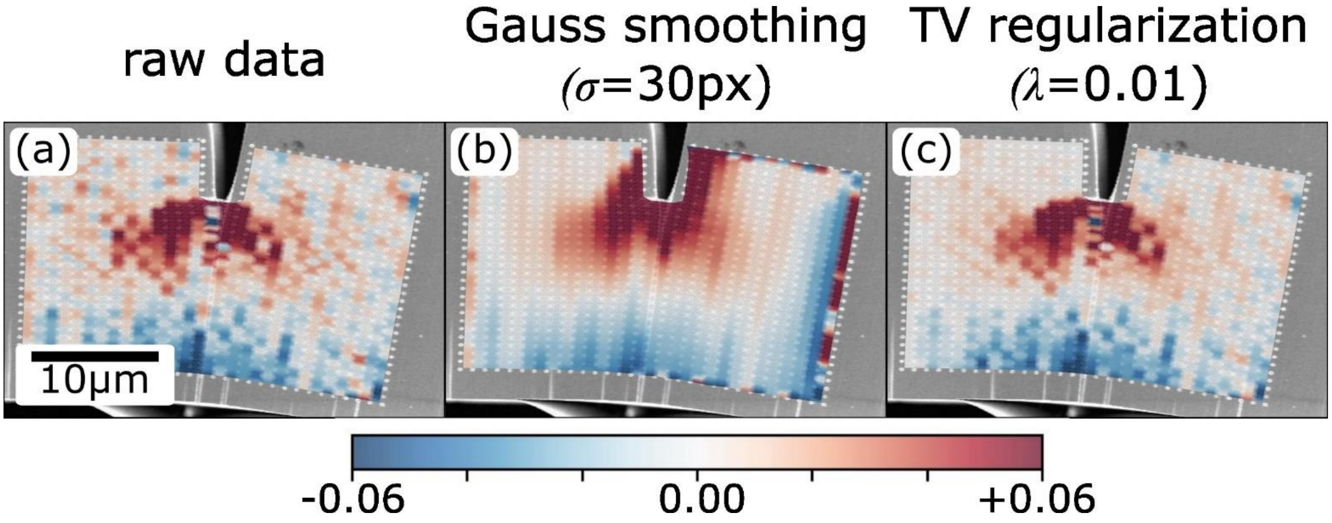

Figure S18. Comparison of different smoothing algorithms

(a) Raw normal strain data of LS3. (b) The same data using gaussian smoothing with a 30px radius as previously determined and (c) the novel TV regularization algorithm with a regularization parameter of λ=0.01, as used in the present work. The micron bar is applicable for all subfigures.

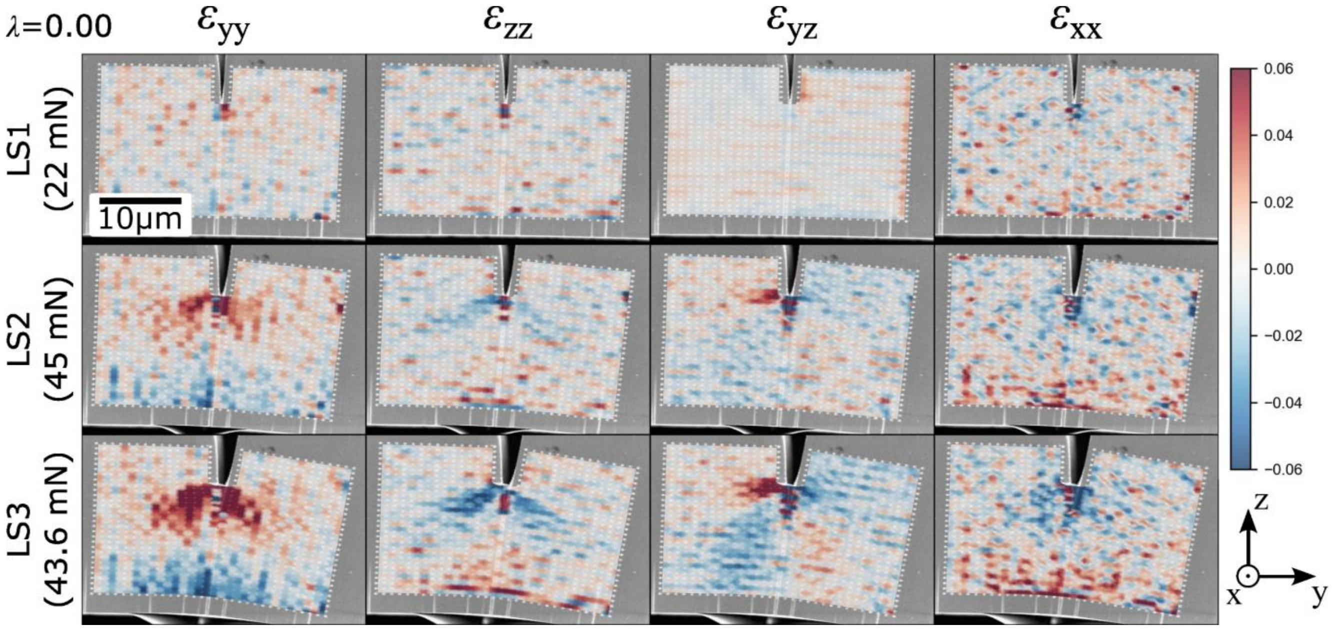

Figure S19. Raw strain data

Raw strain data (normal strains ε_{yy}, ε_{zz}, ε_{xx} and shear strains ε_{yz}, respectively) for all loadsteps without any total variation regularization, λ=0. The micron bar is applicable for all subfigures.

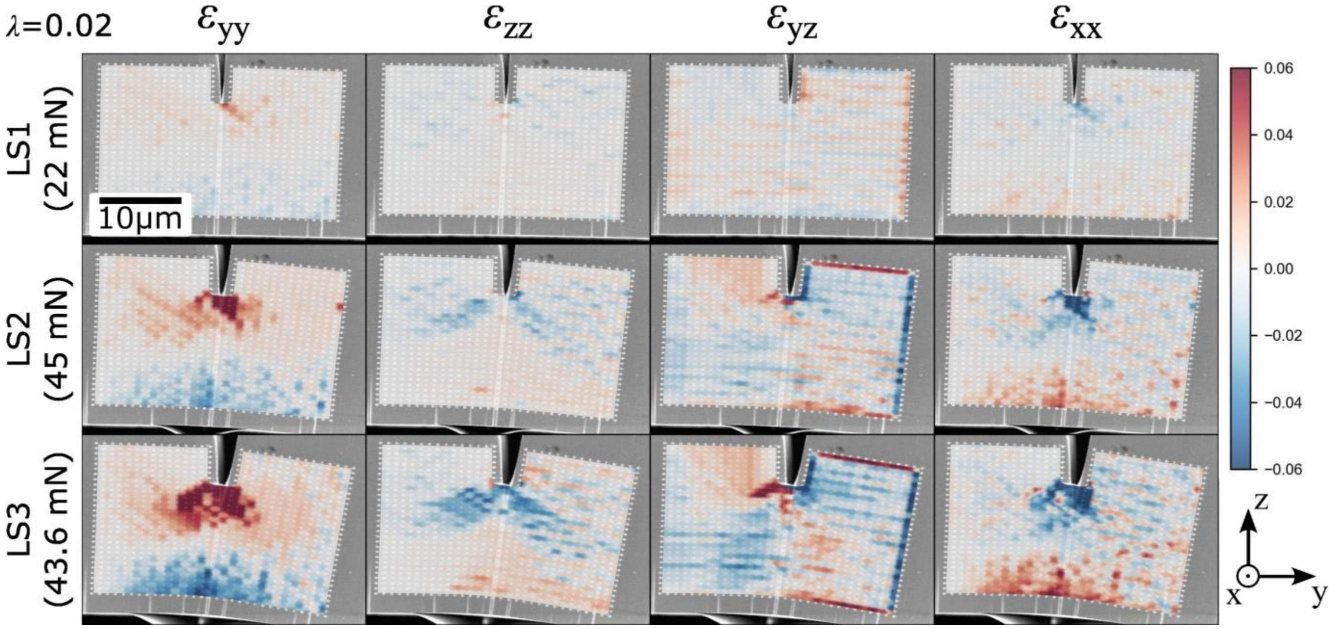

Figure S20. Strain data with a total variation regularization parameter of λ=0.02

Strain data (normal strains ε_{yy}, ε_{zz}, ε_{xx} and shear strains ε_{yz}, respectively) for all loadsteps with a total variation regularization parameter λ=0.02. The micron bar is applicable for all subfigures.

Supplementary Note 9: CSnanoXRD experiment

CSnanoXRD sample alignment

Prior to the CSnanoXRD experiment[51,52], at first the top and bottom surfaces of the HEA cantilever were aligned parallel to the incident X-ray beam direction at two sample *z* positions by performing a set of absorption line-scans along the *z*-axis in various sample orientations φ around the y-axis (Fig. 1) using a point X-ray detector[53,54]. The optimal sample orientation was determined by maximizing the X-ray absorption contrast between HEA and air at both surfaces. Subsequently, two-dimensional (2D) absorption scans (not shown) were performed to locate the notch tip in the centre of the CSnanoXRD mesh scans. The exact detector geometry with respect to the sample was calibrated using a National Institute of Standards and Technology (NIST) corundum powder, yielding a sample-to-detector distance of 132.47 mm. The evaluation of the 2D patterns containing {111} and {200} fcc HEA Debye-Scherrer (DS) rings (Fig. 1) was performed using the pyFAI software package[55,56].

CSnanoXRD data evaluation

The diffuse scattering at relatively small diffraction angles, *i.e.* small-angle X-ray scattering (SAXS), around the beam stop (Fig. 1) originates primarily from electron density variations, such as alternation of materials, presence of grain boundaries, interfaces, cracks, precipitates and pores with sizes of $\sim\lambda/_{\theta}$ where λ represents the X-ray wavelength and θ is the Bragg angle[24,25]. In the present case, the signal scattered onto the 2D detector at the diffraction angles of ~0.05 to ~0.5 deg was integrated radially (θ) and azimuthally (δ) in order to obtain qualitative information primarily on the occurrence of the growing crack within the cantilever.

In order to evaluate $\mathrm{FWHM}(y,z,\Delta\delta_g)$ and stress tensor distributions $\sigma_{ij}(y,z)$ with $i,j = x,y,z$ an integration of the diffraction patterns was performed over the azimuthal angle δ in $\Delta\delta_g$ segments (termed *cakes*) of 10 deg. Thus, 36 radial intensity distributions $I(\theta,\Delta\delta_g)$ were obtained for each exposure in each cake g. The positions of HEA 111 and 200 diffraction peaks $\theta^{hkl}(\Delta\delta_g), g = [1,36]$ and the $\mathrm{FWHM}(y,z,\Delta\delta_g)$ were determined by fitting the X-ray diffraction patterns using a Pseudo-Voigt function for each exposure in every cake g.

Generally, the FWHM evaluated from DS rings is sensitive to the size of coherently diffracting domains, to the presence of structural defects like dislocations, vacancies, and other types of micro- and nanoscopic crystal lattice distortions, which can be denoted as microstrains of 2nd and 3rd order, as well as gradients of strains of 1st order within the gauge volume. Thus, the averaged $\mathrm{FWHM}(y,z) = \frac{1}{36}\sum_{g=1}^{36}\mathrm{FWHM}(y,z,\Delta\delta_g)$ is a vital qualitative parameter to determine the defect accumulation around the crack.

The orientation-dependent lattice plane spacings $d_{\delta\theta}^{\mathrm{hkl}}(y,z)$ of the HEA 111 and 200 peaks are determined by the Bragg equation $d_{\delta\theta}^{\mathrm{hkl}}(y,z) = \frac{n\cdot\lambda}{2\sin[\theta^{\mathrm{hkl}}(\Delta\delta_g)]}$, where λ is the X-ray wavelength and $n = 1$ for the first diffraction order. Subsequently, the orientation-dependent lattice strain $\varepsilon_{\delta\theta}^{\mathrm{hkl}}(y,z)$ for each DS ring *hkl* was determined as follows

$$\varepsilon_{\delta\theta}^{\mathrm{hkl}}(y,z) = \frac{d_{\delta\theta}^{\mathrm{hkl}}(y,z) - d_0^{\mathrm{hkl}}}{d_0^{\mathrm{hkl}}}, \quad \text{(S9.1)}$$

where θ is the diffraction angle, δ the azimuthal orientation on the detector (Fig. 1), $d_{\delta\theta}^{\mathrm{hkl}}(y,z)$ is the lattice plane spacing obtained at the respective diffraction angle θ and azimuthal orientation δ and $d_0^{\mathrm{hkl}} = a_0/(h^2 + k^2 + l^2)$ is the strain-free lattice plane spacing for a particular *hkl* reflection.

The unstressed lattice constant of HEA was determined from diffraction data near the surface of the cantilever before loading (LS0), considering the stress-free out-of-plane (*z*) orientation[57] and found to be $a_0 = 0.35928$ nm. Additionally, the equation system was conditioned to keep the beam (*x*) direction stress-free before loading. The measured orientation-dependent lattice strain $\varepsilon_{\delta\theta}^{\mathrm{hkl}}(y,z)$ is a function of

unknown dependent strain components $\varepsilon_{ij}^{\mathrm{hkl}}$ with $i,j = x,y,z$ defined in the sample coordinate system as follows

$$\varepsilon_{\delta\theta}^{\mathrm{hkl}}(y,z) = \sin^2\theta\varepsilon_{\mathrm{xx}}^{\mathrm{hkl}}(y,z) + \cos^2\theta\sin^2\delta\varepsilon_{\mathrm{yy}}^{\mathrm{hkl}}(y,z) + \cos^2\theta\cos^2\delta\varepsilon_{\mathrm{zz}}^{\mathrm{hkl}}(y,z) - \sin 2\theta\cos\delta\,\varepsilon_{\mathrm{xz}}^{\mathrm{hkl}}(y,z) + \cos^2\theta\sin 2\delta\,\varepsilon_{\mathrm{yz}}^{\mathrm{hkl}}(y,z) - \sin 2\theta\sin\delta\,\varepsilon_{\mathrm{xy}}^{\mathrm{hkl}}(y,z). \quad \text{(S9.2)}$$

Using X-ray elastic constants S_1^{hkl} and $\frac{1}{2}S_2^{\mathrm{hkl}}$ the strain components $\varepsilon_{ij}^{\mathrm{hkl}}$ of Eq. S4 can be replaced by the components of the stress tensor $\sigma_{ij}(y,z)$ defined in the sample coordinate system as follows

$$\varepsilon_{\delta\theta}^{\mathrm{hkl}}(y,z) = S_1^{\mathrm{hkl}}\left[\sigma_{\mathrm{xx}}^{\mathrm{ev}}(y,z) + \sigma_{\mathrm{yy}}(y,z) + \sigma_{\mathrm{zz}}(y,z)\right] + \frac{1}{2}S_2^{\mathrm{hkl}}\left[\sin^2\theta\,\sigma_{\mathrm{xx}}^{\mathrm{ev}}(y,z) + \cos^2\theta\sin^2\delta\,\sigma_{\mathrm{yy}}(y,z) + \cos^2\theta\cos^2\delta\,\sigma_{\mathrm{zz}}(y,z) - \sin 2\theta\cos\delta\,\sigma_{\mathrm{xz}}(y,z) + \cos^2\theta\sin 2\delta\,\sigma_{\mathrm{yz}}(y,z) - \sin 2\theta\sin\delta\,\sigma_{\mathrm{xy}}(y,z)\right]. \quad \text{(S9.3)}$$

X-ray elastic constants of $S_1^{111} = -4.21\times 10^{-4}\mathrm{GPa}^{-1}$, $\frac{1}{2}S_2^{111} = 3.847\times 10^{-3}\mathrm{GPa}^{-1}$, $S_1^{200} = -2.109\times 10^{-3}\mathrm{GPa}^{-1}$ and $\frac{1}{2}S_2^{200} = 8.910\times 10^{-3}\mathrm{GPa}^{-1}$ were adopted from literature[58]. The evaluation of the 3D stress tensor was supported by using the diffracted intensities from the 111 and 200 DS rings have highly different X-ray elastic constants, as a consequence of the high crystallographic anisotropy represented by a Zener ratio of $Z = 4.2$[59,60]. Therefore, the individual ring's changes $2\theta(\delta)$ pursuant to the stress tensor are highly different (Fig. S21), which in turn allows to (at least semi-quantitative) retrieve σ_{xx}.

Across the region of interests of the cantilever, the stress state was determined by least-squares fitting of an overdetermined system of 36 linear equations based on the measured orientation-dependent lattice strain $\varepsilon_{\delta\theta}^{\mathrm{hkl}}(y,z)$ evaluated for each cake g.

The equivalent von-Mises stress, was calculated as a common yield criterion according to[32]:

$$\sigma_{\mathrm{von\ Mises}}(y,z) = \sqrt{\frac{1}{2}\left[\left(\sigma_{\mathrm{xx}}(y,z) - \sigma_{\mathrm{yy}}(y,z)\right)^2 + \left(\sigma_{\mathrm{xx}}(y,z) - \sigma_{\mathrm{zz}}(y,z)\right)^2 + \left(\sigma_{\mathrm{yy}}(y,z) - \sigma_{\mathrm{zz}}(y,z)\right)^2\right] + 3\left(\sigma_{\mathrm{xy}}^2 + \sigma_{\mathrm{xz}}^2 + \sigma_{\mathrm{yz}}^2\right)}. \quad \text{(S9.4)}$$

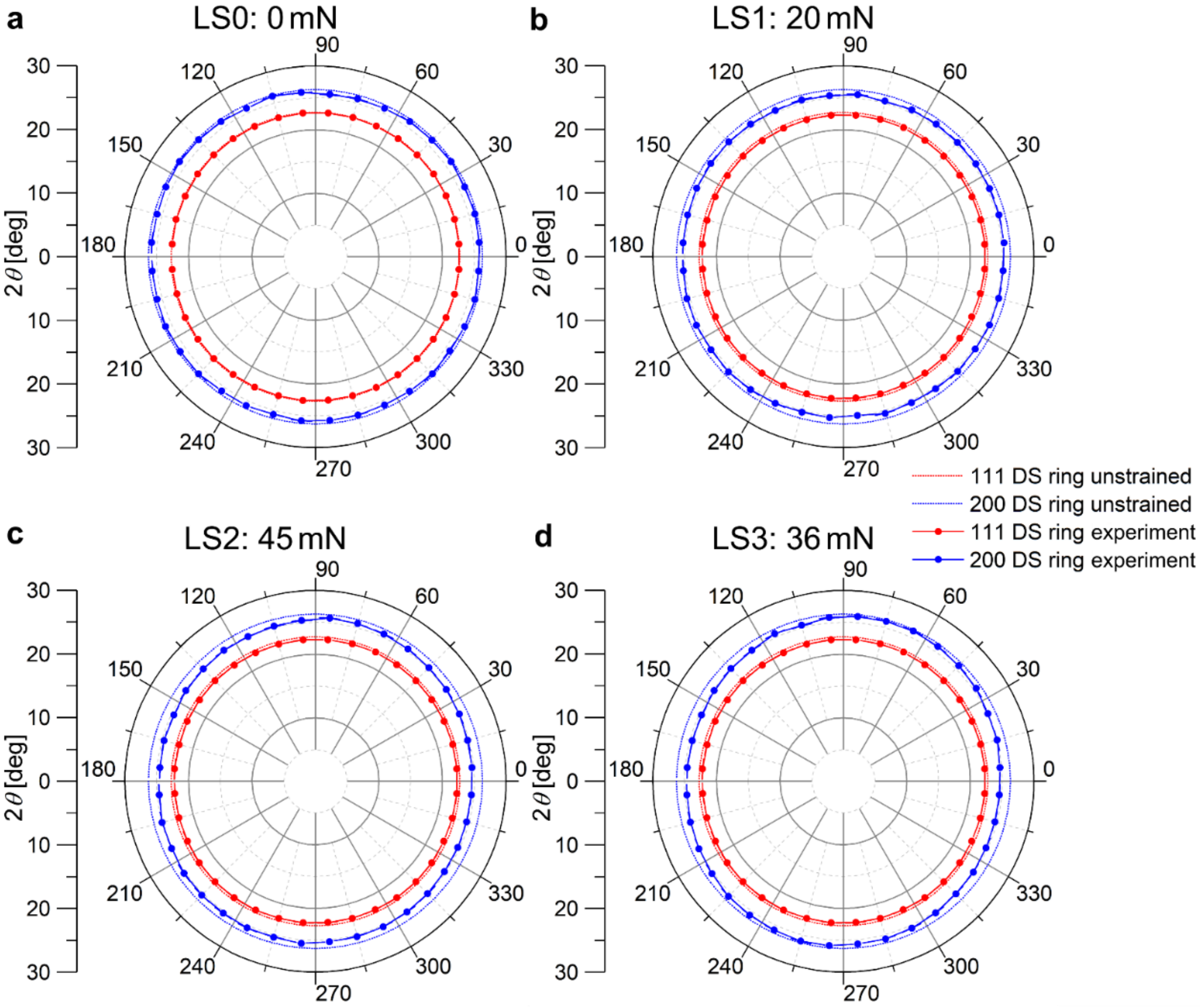

Figure S21. 2θ-δ plots in polar coordinates.

2θ-δ plots in polar coordinates are presented in (a), (b), (c) and (d) for the DS rings recorded directly in front of the crack tip for LS0, LS1, LS2 and LS3, respectively. The hydrostatic tension in front of the crack tip at LS1 can be seen by the δ-independent reduction of 2θ. Please note that the actual differences between the unstrained DS ring and the strained DS rings were magnified by a factor of 5.

Supplementary Note 10: Detailed FE-analysis

FE model setup

A 3D elasto-plastic Finite Element (FE) model was employed to simulate the indentation process of the earlier-mentioned pre-notched cantilever; using the commercial software COMSOL Multiphysics® was utilised. The material constitutive law was modelled using the following Ramberg-Osgood hardening law (Eq. S1.1) as discussed in Supplementary Note 1, with the elastic, yield stress and hardening exponent n equal to 205 GPa, 2355 MPa and 50, respectively. The adopted plasticity formulation accounts for small plastic strains, with the yield function determined by the Von Mises stress criterion.

Thanks to the symmetric nature of the studied model and its loading conditions, only half of the tested component was modelled. The mechanical constraints applied to the numerical model are shown in Figure S22.

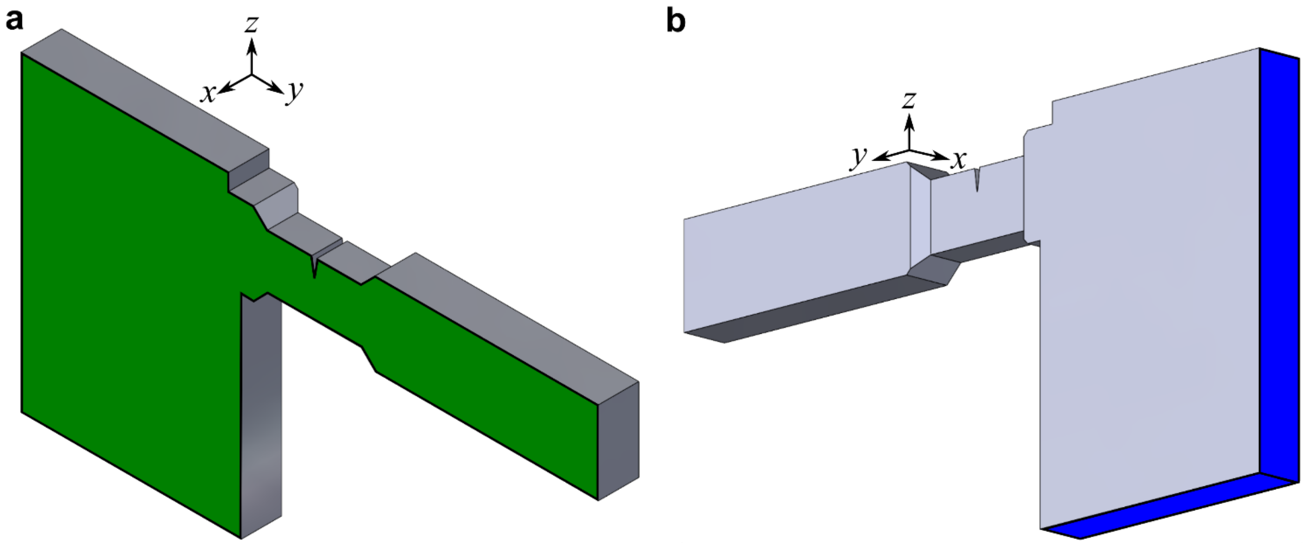

Figure S22. Representation of the 3D model employed for the FE simulation.

In (a) the model is shown, with the symmetry condition highlighted in green, while in (b) the surfaces where far-field boundary conditions were applied are highlighted in blue. For the highlighted surfaces in (b) all degrees of freedom are constrained.

Concerning the local displacement introduced by the nano-indenter as a function of a pseudo-time, u(t*), such a boundary condition was implemented over a line, as illustrated in Figure S23. Given the large deformation expected by this test, geometry non-linearities were accounted. The mechanical model exploited second-order serendipity hexahedral elements with 20 nodes, having quadratic shape functions. To ensure a high accuracy of the solution, the model was discretised using a structured mesh approach, with mesh refinement at the crack tip location and neighbouring regions, as illustrated in Figure 4. A systematic mesh convergence test was conducted to assess the effectiveness of the mesh in capturing stress gradients; a mesh consisting of 60216 elements was selected for the simulations. The time-dependent solver utilizes the generalized-α method, implementing an implicit scheme that ensures numerical stability and accuracy. Additionally, it incorporates an intermediate time-stepping strategy, which imposes an extra temporal constraint on the solver, beyond the selected time step, to enhance solution robustness.

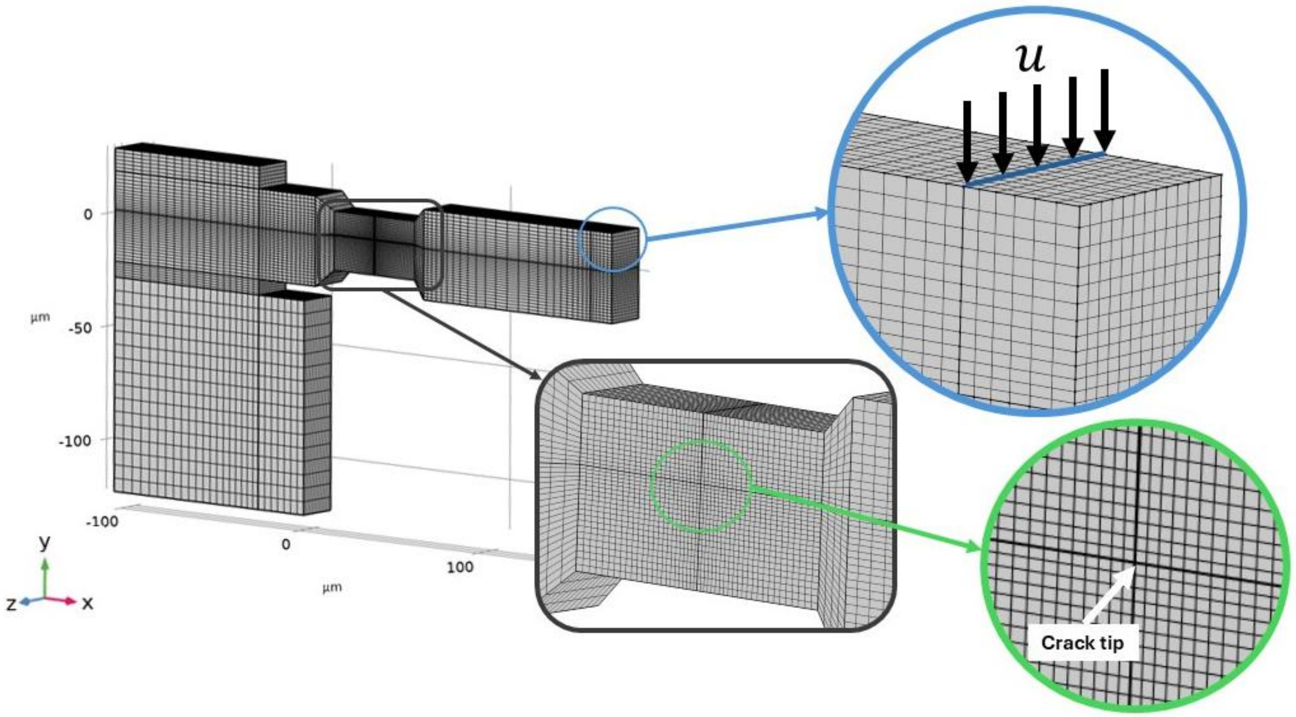

Figure S23. Overview of the structured mesh employed in the FE model.
In the insets, details of the position of the imposed line displacement (blue), the area covered by the *in situ* experiments (black) and the immediate surroundings of the crack tip (green) are shown.

To simulate crack propagation at the three reference conditions (i.e., LS1, LS2, and LS3), a Cohesive Zone Model (CZM) was employed in COMSOL Multiphysics®. Therefore, cohesive elements were modelled along the crack bisector. The CZM utilizes a traction-separation law to describe the relationship between the cohesive traction T and the corresponding displacement jump δ across the cohesive interface. The traction-separation laws adopted in this study assumed a bilinear form, characterized by an initial linear elastic region followed by a softening phase that governs the fracture process. The key parameters of the CZM, including the maximum cohesive traction T_{max} and the energy release rate G_c, which were iteratively determined by matching the force-displacement curve obtained from simulations with experimental data until a good correlation was achieved.

The CZM provided values for crack propagation observed during the experiments, enabling a detailed and independent modelling of the points LS1, LS2, and LS3 using reasonable crack propagation values. This approach ensured a robust simulation of crack initiation and growth under the complex loading conditions imposed during the indentation test. The contour plots displayed in Figure S24 show the crack propagation

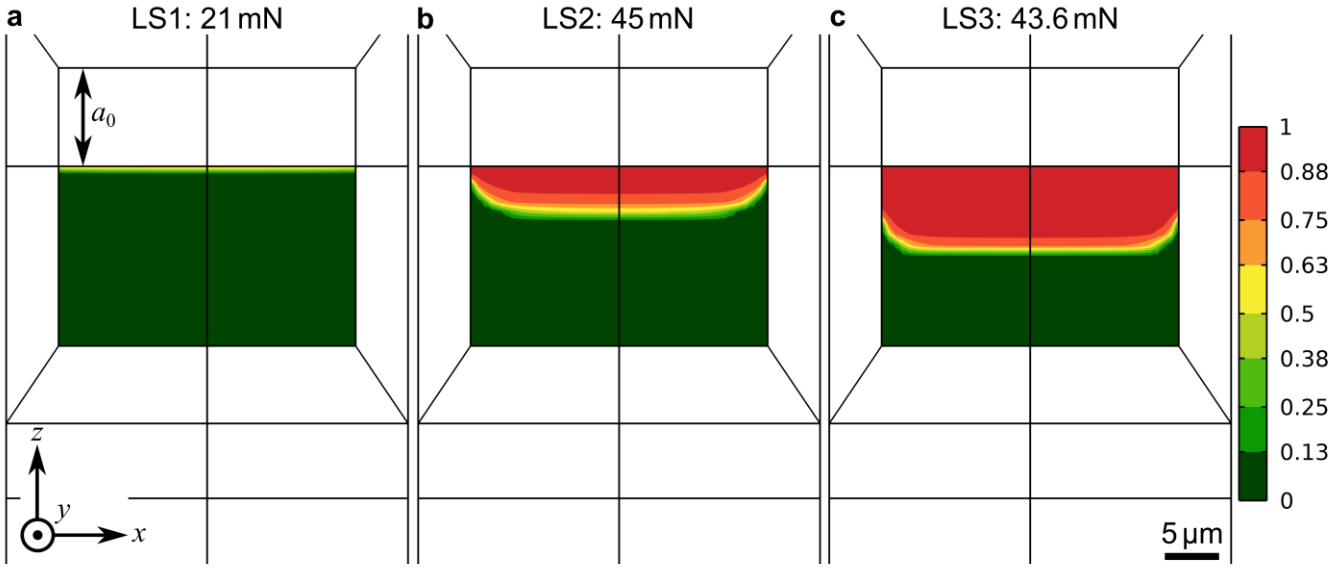

Figure S24. Crack bisector planes and crack growth obtained by the cohesive zone model.
In (a-c), the red shading highlights the separation, indicating crack growth, while green shaded parts are still adherent. The scale bar in (c) is applicable for all images.

FE post-processing

Upon solving the FE problem, several results could be extracted. Firstly, the integral value of the reaction force in the *y*-direction along the imposed displacement line, as illustrated in Figure 2, provided the basis for evaluating the force vs. displacement curve that is later compared with the experimental measurements. Following, to compare the stress field at the crack tip with that obtained experimentally, through-thickness averaged values were extracted to account for the exact gauge volume employed during the CSnanoXRD experiment. This allowed the determination of K_I, and K_{II} for the points of interest LS1, LS2, and LS3, respectively. The crack opening stresses (COS) and the along the crack tip bisector were extracted from the averaged stress field this, again, enabled a fair comparison with the experimentally retrieved values.

The values of J_{avg}, were obtained using the dedicated algorithm available in COMSOL Multiphysics. This function computes the 2D *J*-integral at multiple cross-sections along the specimen's thickness, generating a continuous profile of the *J*-integral as a function of thickness. By averaging this profile across the sample's thickness, it is then possible to obtain J_{avg}.

Supplementary Note 11: J_{comb} integration parameter analysis

The numerical integration for J_{comb} is conducted with equal angular spacing along each contour, *i.e.* the same number of integration points along the contour, independent of the size or shape. While this means that for longer contours, the individual integration segments are larger than for shorter contours. However, as the strain field is linearised between individual features with roughly 900 nm spacing, using equally spaced integration distances considerably lower than that will not alter the final integrated value to an extend that outweighs unaccountable experimental errors. To quantify this, the J_{comb} analysis was conducted on various integration paths using data from LS3 with changing angular integration steps from 0.1 to 10 deg and summarized in Fig. S25. There, it is evident that on average, even using integration steps of 1° do not alter the J_{comb} result by more than 1%, while larger integration steps can lead to average deviations of up to ~5%. The chosen integration step size for the data presented in the manuscript was 0.5 deg, leading to a maximum integration step size of ~100 nm for the largest contour sizes (24 μm). This suggests validity of the data, given the experimental constraint.

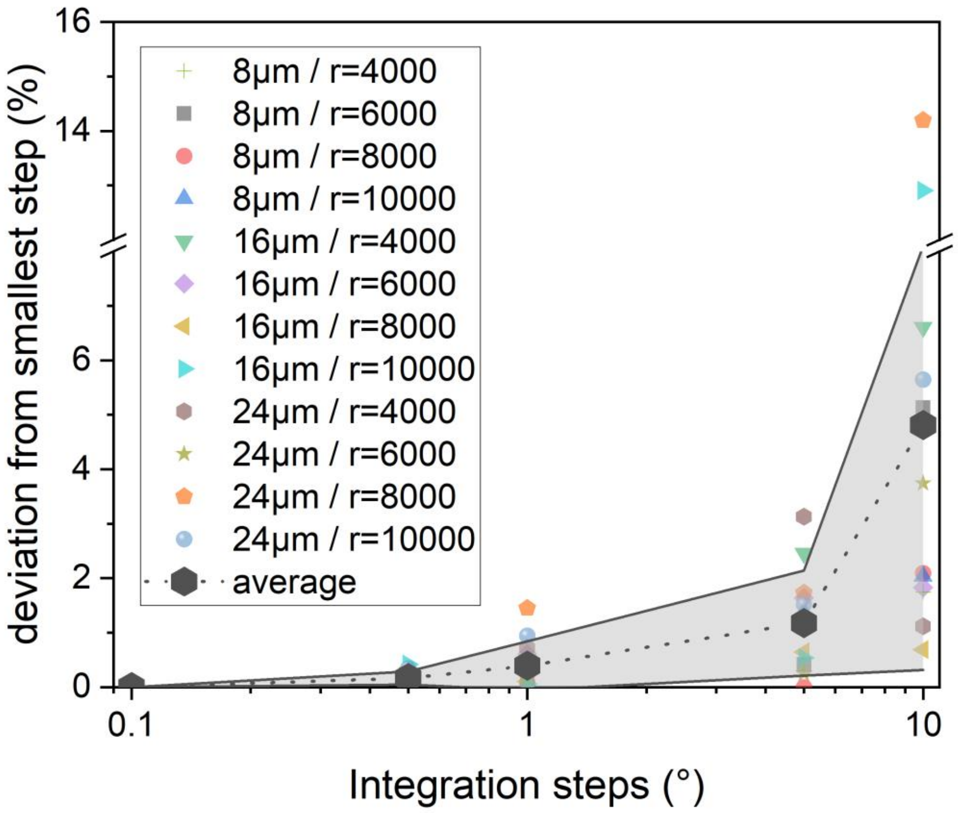

Figure S25. Integration step size analysis.

The percentage deviation from a smallest integration increment of 0.1 deg is shown for various contours (narrow:8 μm, medium:16 μm, wide:24 μm) with respect to increasing angular integration spacings up to 10 deg. The average of all data is shown in black hexagons with the shaded area representing a bound of one standard deviation.

Supplementary References

1. Schuh, B. *et al.* Mechanical properties, microstructure and thermal stability of a nanocrystalline CoCrFeMnNi high-entropy alloy after severe plastic deformation. *Acta Mater.* **96**, 258–268 (2015).

2. Pillmeier, S. *et al.* On the fatigue crack growth behavior of nanocrystalline CrMnFeCoNi. *Int. J. Fatigue* **188**, 108530 (2024).

3. Maier-Kiener, V., Schuh, B., George, E. P., Clemens, H. & Hohenwarter, A. Insights into the deformation behavior of the CrMnFeCoNi high-entropy alloy revealed by elevated temperature nanoindentation. *J. Mater. Res.* **32**, 2658–2667 (2017).

4. Maier-Kiener, V., Schuh, B., George, E. P., Clemens, H. & Hohenwarter, A. Nanoindentation testing as a powerful screening tool for assessing phase stability of nanocrystalline high-entropy alloys. *Mater. Des.* **115**, 479–485 (2017).

5. Schuh, B. *et al.* Deformation Induced Structure and Property Changes in a Nanostructured Multiphase CrMnFeCoNi High-Entropy Alloy. *Nanomaterials* **13**, 1–12 (2023).

6. Alfreider, M., Meindlhumer, M., Maier-Kiener, V., Hohenwarter, A. & Kiener, D. Extracting information from noisy data: strain mapping during dynamic in situ SEM experiments. *J. Mater. Res.* **36**, 2291–2304 (2021).

7. Huang, X., Hansen, N. & Tsuji, N. Hardening by annealing and softening by deformation in nanostructured metals. *Science (80-.).* **312**, 249–251 (2006).

8. Leitner, A., Maier-kiener, V. & Kiener, D. Essential refinements of spherical nanoindentation protocols for the reliable determination of mechanical flow curves. *Mater. Des.* **146**, 69–80 (2018).

9. Tang, F. & Schoenung, J. M. Strain softening in nanocrystalline or ultrafine-grained metals: A mechanistic explanation. *Mater. Sci. Eng. A* **493**, 101–103 (2008).

10. He, X., Zhu, L., Liu, J. & An, L. Grain growth-induced strain softening in nanocrystalline magnesium: Experiments and modelling. *Mater. Res. Express* **6**, (2019).

11. Meindlhumer, M. *et al.* Evolution of stress fields during crack growth and arrest in a brittle-ductile CrN-Cr clamped-cantilever analysed by X-ray nanodiffraction and modelling. *Mater. Des.* **198**, 109365 (2021).

12. Ecker, W. *et al.* Nanoscale evolution of stress concentrations and crack morphology in multilayered CrN coating during indentation : Experiment and simulation. *Mater. Des.* **188**, 108478 (2020).

13. Zeilinger, A. *et al.* In-situ Observation of Cross-Sectional Microstructural Changes and Stress Distributions in Fracturing TiN Thin Film during Nanoindentation. *Sci. Rep.* **6**, 22670 (2016).

14. Todt, J. *et al.* Indentation response of a superlattice thin film revealed by in-situ scanning X-ray nanodiffraction. *Acta Mater.* **195**, 425–432 (2020).

15. Lotze, G., Iyer, A. H. S., Bäcke, O., Kalbfleisch, S. & Colliander, M. H. In situ characterization of stresses , deformation and fracture of thin films using transmission X-ray nanodiffraction microscopy. *J. Synchrotron Radiat.* **31**, 1–13 (2024).

16. MacKenzie, P., McKelvie, J., McDonach, A. & Walker, C. A. Measurement of Poisson's ratio through the elastic-plastic transition. *Strain* **22**, 13–19 (1986).

17. Narasimhan, R. & Rosakis, A. J. Three-dimensional effects near a crack tip in a ductile three-point bend specimen: Part I-a numerical investigation. *J. Appl. Mech. Trans. ASME* **57**, 607 (1990).

18. Lopez-Crespo, P. *et al.* Characterisation of overloads in fatigue by 2D strain mapping at the surface and in the bulk. *Fatigue Fract. Eng. Mater. Struct.* **39**, 1040–1048 (2016).

19. Lopez-Crespo, P., Peralta, J. V., Kelleher, J. F. & Withers, P. J. In situ through-thickness analysis of crack tip fields with synchrotron X-ray diffraction. *Int. J. Fatigue* **127**, 500–508 (2019).

20. Robertson, S. W., Mehta, A., Pelton, A. R. & Ritchie, R. O. Evolution of crack-tip transformation zones in superelastic Nitinol subjected to in situ fatigue: A fracture mechanics and synchrotron X-ray microdiffraction analysis. *Acta Mater.* **55**, 6198–6207 (2007).

21. Jing, P., Khraishi, T. & Gorbatikh, L. Closed-form solutions for the Mode II crack tip plastic zone shape. *Int. J. Fract.* **122**, 137–142 (2003).

22. Skrotzki, W. *et al.* Microstructure and texture evolution during severe plastic deformation of CrMnFeCoNi high-entropy alloy. *IOP Conf. Ser. Mater. Sci. Eng.* **194**, (2017).

23. Skrotzki, W. *et al.* Microstructure, texture, and strength development during high-pressure torsion of crmnfeconi high-entropy alloy. *Crystals* **10**, (2020).

24. Feigin, L. A. & Svergun, D. I. *Structure Analysis by Small-Angle X-Ray and Neutron Scattering*. (Springer New York, N, 1987). doi:https://doi.org/10.1007/978-1-4757-6624-0.

25. Meindlhumer, M. *et al.* Nanoscale residual stress and microstructure gradients across the cutting edge area of a TiN coating on WC-Co. *Scr. Mater.* **182**, 11–15 (2020).

26. Meindlhumer, M. *et al.* Nanoscale stress distributions and microstructural changes at scratch track cross-sections of a deformed brittle-ductile CrN-Cr bilayer. *Mater. Des.* **195**, 109023 (2020).

27. Schmidt, S. *et al.* Watching the Growth of Bulk Grains During Recrystallization of. *Science (80-.).* **305**, 229–232 (2004).

28. Zhang, Y. B., Godfrey, A., Liu, Q., Liu, W. & Jensen, D. J. Analysis of the growth of individual grains during recrystallization in pure nickel. *Acta Mater.* **57**, 2631–2639 (2009).

29. Kapp, M. W., Kremmer, T., Motz, C., Yang, B. & Pippan, R. Structural instabilities during cyclic loading of ultrafine-grained copper studied with micro bending experiments. *Acta Mater.* **125**, 351–358 (2017).

30. ISO/IEC 98-3:2008 Uncertainty of measurement - Part 3: Guide to the expression of uncertainty in measurement (GUM:1995), (ISO, 2008).

31. Anderson, T. L. L. *Fracture mechanics, fundamentals and applications*. *CRC Press* (CRC Press, 2017). doi:/10.1201/9781315370293.

32. S.Timoshenko. *Strength Of Materials part I and II.* (D. Van Nostrand Company, Inc., 1940).

33. Laplanche, G. *et al.* Temperature dependencies of the elastic moduli and thermal expansion coefficient of an equiatomic , single-phase CoCrFeMnNi high-entropy alloy. *J. Alloys Compd.* **623**, 348–353 (2015).

34. Hancock, J. W. & Mackenzie, A. C. On the mechanisms of ductile failure in high-strength steels subjected to multi-axial stress-states. *J. Mech. Phys. Solids* **24**, 147–160 (1976).

35. Renk, O. *et al.* Increasing the strength of nanocrystalline steels by annealing: Is segregation necessary? *Scr. Mater.* **95**, 27–30 (2015).

36. Alfreider, M., Issa, I., Renk, O. & Kiener, D. Probing defect relaxation in ultra-fine grained Ta using micromechanical spectroscopy. *Acta Mater.* **185**, 309–319 (2020).

37. Zhang, X. X. *et al.* Quantifying internal strains, stresses, and dislocation density in additively manufactured AlSi10Mg during loading-unloading-reloading deformation. *Mater. Des.* **198**, 1–9 (2021).

38. Kapp, M. W. *et al.* Plastic strain triggers structural instabilities upon cyclic loading in ultrafine-grained nickel. *Acta Mater.* **200**, 136–147 (2020).

39. Greaves, G. N., Greer, A. L., Lakes, R. S. & Rouxel, T. Poisson's ratio and modern materials. *Nat. Mater.* **10**, 823–837 (2011).

40. Priester, L. 'Dislocation-interface' Interaction - Stress accommodation processes at interfaces. *Mater. Sci. Eng. A* **309**–**310**, 430–439 (2001).

41. Varin, R. A. Spreading of extrinsic grain boundary dislocations in austenitic steel. *Phys. Status Solidi* **52**, 347–356 (1979).

42. Wilde, G. & Divinski, S. Grain boundaries and diffusion phenomena in severely deformed materials. *Mater. Trans.* **60**, 1302–1315 (2019).

43. Zaher, G. *et al.* Influence of strain rate and Sn in solid solution on the grain refinement and crystalline defect density in severely deformed Cu. *Mater. Today Commun.* **26**, 101746 (2021).

44. Pfeifenberger, M. J. *et al.* The use of femtosecond laser ablation as a novel tool for rapid micro-mechanical sample preparation. *Mater. Des.* **121**, 109–118 (2017).

45. Alfreider, M., Kozic, D., Kolednik, O. & Kiener, D. In-situ elastic-plastic fracture mechanics on the microscale by means of continuous dynamical testing. *Mater. Des.* **148**, 177–187 (2018).

46. Tada, H., Paris, P. C. & Irwin, G. R. *The Stress Analysis of Cracks Handbook.* (ASME Press, 2000).

47. Alfreider, M., Kolitsch, S., Wurster, S. & Kiener, D. An analytical solution for the correct determination of crack lengths via cantilever stiffness. *Mater. Des.* **194**, 108914 (2020).

48. Alfreider, M., Zechner, J. & Kiener, D. Addressing Fracture Properties of Individual Constituents Within a Cu-WTi-SiOx-Si Multilayer. *JOM* **72**, 4551–4558 (2020).

49. Rudin, L. I., Osher, S. & Fatemi, E. Nonlinear total variation based noise removal algorithm. *Phys. D* **60**, 259–268 (1992).

50. Goldstein, T. & Osher, S. The Split Bregman Method for L1-Regularized Problems. *SIAM J. Imaging Sci.* **2**, 323–343 (2009).

51. Keckes, J., Todt, J., Meindlhumer, M. & Ziegelwanger, T. In situ mapping of crack progression in nanocrystalline FeCr: nanoscale stress-strain evolution in nanocrystalline microstructure [Data set]. European Synchrotron Radiation Facility. (2024) doi:doi.org/10.15151/ESRF-ES-514138045.

52. Rettenwander, D. *et al.* Scanning and In-situ MLL Development for X-ray Nanodiffraction on Thin Film, Space and Additively Manufactured Applications [Data set]. European Synchrotron Radiation Facility. (2025) doi:10.15151/ESRF-ES-644167190.

53. Stefenelli, M. *et al.* X-ray nanodiffraction reveals stress distribution across an indented multilayered CrN–Cr thin film. *Acta Mater.* **85**, 24–31 (2015).

54. Keckes, J. *et al.* 30 nm X-ray focusing correlates oscillatory stress, texture and structural defect gradients across multilayered TiN-SiOx thin film. *Acta Mater.* **144**, 862–873 (2018).

55. Kieffer, J. & Karkoulis, D. PyFAI, a versatile library for azimuthal regrouping. *J. Phys. Conf. Ser.* **425**, 8–13 (2013).

56. Ashiotis, G. *et al.* The fast azimuthal integration Python library: PyFAI. *J. Appl. Crystallogr.* **48**, 510–519 (2015).

57. Bartosik, M. *et al.* Cross-sectional X-ray nanobeam diffraction analysis of a compositionally graded CrNx thin film. *Thin Solid Films* vol. 542 1–4 (2013).

58. Tian, F., Varga, L. K., Chen, N., Delczeg, L. & Vitos, L. Ab initio investigation of high-entropy alloys of 3d elements. *Phys. Rev. B - Condens. Matter Mater. Phys.* **87**, 1–8 (2013).

59. Tian, F., Károly, L., Shen, J. & Vitos, L. Calculating elastic constants in high-entropy alloys using the coherent potential approximation : Current issues and errors. *Comput. Mater. Sci.* **111**, 350–358 (2016).

60. Clarene Zener. Theory of the Elasticity of Polycrystals with Viscous Grain Boundaries. *Phys. Rev.* **60**, 906–908 (1941).

Movie S1.
This movie shows the progression of the raw ε_{yy} strain component during the *in situ* SEM experiment.

Movie S2.
This movie shows the progression of the raw ε_{yz} strain component during the *in situ* SEM experiment.

Movie S3.
This movie shows the progression of the raw ε_{zz} strain component during the *in situ* SEM experiment.

Movie S4.
This movie shows the progression of the raw ε_{xx} strain component during the *in situ* SEM experiment.
