# Supplementary material for: Resolving the fundamentals of the J-integral concept by multi-method in situ nanoscale stress-strain mapping
Source: Commun Mater. 2025 Feb 22;6(1):35. doi: 10.1038/s43246-025-00752-z (PMC11846709; doi:10.1038/s43246-025-00752-z)
Supplement: Supplementary file 2 — Description of Additional Supplementary Files [file 43246_2025_752_MOESM2_ESM.pdf]

Description of Additional Supplementary Files

File name: Movie S1.

File description: This movie shows the progression of the raw ε_{yy} strain component during the in situ SEM experiment.

File name: Movie S2.

File description: This movie shows the progression of the raw ε_{yz} strain component during the in situ SEM experiment.

File name: Movie S3.

File description: This movie shows the progression of the raw ε_{zz} strain component during the in situ SEM experiment.

File name: Movie S4.

File description: This movie shows the progression of the raw ε_{xx} strain component during the in situ SEM experiment.
